# Photoactivatable V‐Shaped Bifunctional Quinone Methide Precursors as a New Class of Selective G‐quadruplex Alkylating Agents

**DOI:** 10.1002/chem.202200734

**Published:** 2022-05-12

**Authors:** Alberto Lena, Alessandra Benassi, Michele Stasi, Christine Saint‐Pierre, Mauro Freccero, Didier Gasparutto, Sophie Bombard, Filippo Doria, Daniela Verga

**Affiliations:** ^1^ Department of Chemistry University of Pavia Viale Taramelli 10 27100 Pavia Italy; ^2^ CNRS UMR9187 INSERM U1196 Institut Curie PSL Research University 91405 Orsay France; ^3^ CNRS UMR9187 INSERM U1196 Université Paris-Saclay 91405 Orsay France; ^4^ University Grenoble Alpes CEA CNRS IRIG SyMMES-UMR5819 38054 Grenoble France; ^5^ Present Address: Department of Chemistry Technical University of Munich Lichtenbergstraße 4 85748 Garching Germany

**Keywords:** cross-linking agents, G-quadruplexes, G4 ligands, photochemistry, quinone methides

## Abstract

Combining the selectivity of G‐quadruplex (G4) ligands with the spatial and temporal control of photochemistry is an emerging strategy to elucidate the biological relevance of these structures. In this work, we developed six novel V‐shaped G4 ligands that can, upon irradiation, form stable covalent adducts with G4 structures via the reactive intermediate, quinone methide (QM). We thoroughly investigated the photochemical properties of the ligands and their ability to generate QMs. Subsequently, we analyzed their specificity for various topologies of G4 and discovered a preferential binding towards the human telomeric sequence. Finally, we tested the ligand ability to act as photochemical alkylating agents, identifying the covalent adducts with G4 structures. This work introduces a novel molecular tool in the chemical biology toolkit for G4s.

## Graphical abstract

**G4 ligand Quinone** Methide precursors. A new family of G‐quadruplex alkylating compounds able to generate quinone methides (QM) through photochemical activation has been designed and synthesized. In the presence of the human telomeric sequence, ammonium salt precursors showed the ability to generate mono‐ and crosslinking products identified both by gel and MALDI‐ToF mass spectrometry analysis.

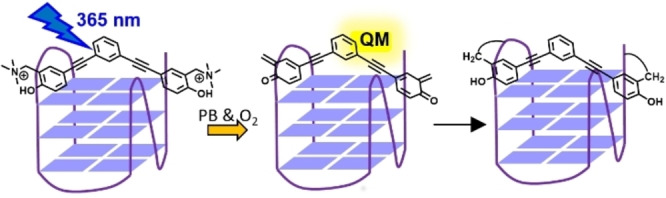

## Introduction

Repetitive guanine‐rich DNA and RNA sequences can fold into non‐canonical four‐stranded secondary structures known as G‐quadruplexes (G4s). The latter are generated by the stacking of several guanine quartets that are held together by Hoogsteen hydrogen bonds and coordination to central monovalent cations.[^1^, ^2^] These secondary structures are believed to play crucial roles as regulatory elements during DNA and RNA transactions, such as replication, transcription, translation, splicing, repair, and recombination.[^3^, ^4^, ^5^, ^6^] Despite corroborating evidence supports the *in vitro* existence of G4 structures, their *in vivo* occurrence as well as the consensus sequence is still a matter of debate. Bioinformatics studies predict the existence of a large number of G4 structures within the genome, in particular in promoters, coding regions, introns, and untranslated (UTRs) regions of genes and intergenic regions.[^7^, ^8^, ^9^] Moreover, recently, next‐generation sequencing (NGS) experiments, performed in the presence of G4‐specific antibodies alone or in combination with G4 selective binding molecules (G4 ligands),[^10^, ^11^, ^12^] have helped to gain insights into G4 structures frequency and functional relevance in both human genome and transcriptome.[^13^, ^14^, ^15^, ^16^, ^17^] High‐throughput *in vitro* polymerase stop assay (G4‐Seq) led to the identification of over 700,000 potential DNA quadruplex‐forming sequences in the human genome.^[13]^ Differently, by means of G4 ChiP‐Seq experiments, only 10,000 G4 sites specifically enriched in nucleosome‐depleted regions were identified, suggesting that chromatin state plays a fundamental role in G4 formation.^[14]^ Moreover, through a reverse transcriptase stalling assay (rG4‐Seq), it has been found that RNA G4 landscape in the human transcriptome comprises more than 13,400 quadruplex‐forming sequences.^[16]^ This large body of data is compelling of G4 existence and potential druggability in cells and without a doubt suggests that G4 ligands are highly valuable chemical tools necessary to disclose G4 DNA and RNA biological functions. Up to date, several experiments have highlighted the ability of small synthetic molecules to bind and stabilize G4s *in vitro* and to interfere with biological processes involving genes containing those structures.[^18^, ^19^, ^20^, ^21^]

**Scheme 1:**
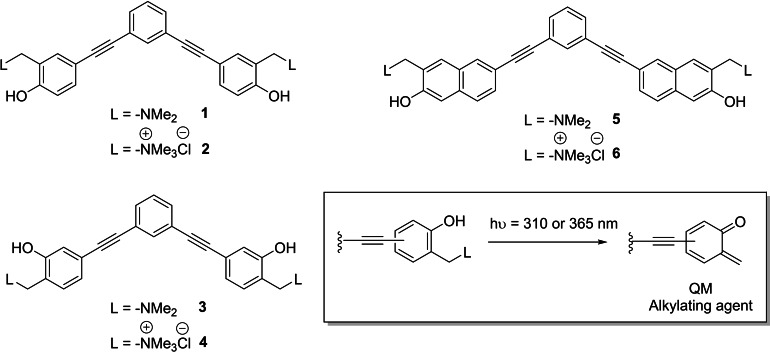
Structures of the novel water‐soluble extended aromatic G4 ligands characterized by a planar V‐shaped rigid scaffold functionalized with photoactivatable QM precursors. Inset: general photogeneration of *o*‐QM intermediate.

Together with reversible G4 ligands, reactive compounds capable of forming covalent bonds with G4 structures upon thermal,[^22^, ^23^] photochemical,[^24^, ^25^] target proximity[^26^, ^27^] and singlet oxygen^[28]^ activation have been developed. The photochemical activation strategies, which include singlet oxygen activation, exploit a new class of promising G4 ligands with the potential to be harnessed for identification and isolation of G4s. Differently from thermal activation, photoaffinity labelling of G4 ligands encloses the potential to obtain spatial and temporal control over the alkylation event, providing an added value to diagnostic and therapeutic strategies. However, to date, only a few examples of photo‐alkylating G4 ligands have been described: compounds based on arylazide, benzophenone^[24]^ and diazirine[^29^, ^30^] excited state chemistry while other ligands exploit the generation of phenoxyl radical via photo‐induced electron transfer.^[25]^ Quinone methides (QMs) are interesting reactive carbon electrophile intermediates employed to target biological macromolecules such as proteins[^31^, ^32^] and nucleic acids.[^22^, ^33^, ^34^, ^35^, ^36^] Consequently, together with other activation strategies, biocompatible photochemical generation of QMs has been thoroughly investigated.[^37^, ^38^, ^39^, ^40^] Experimental evidence confirms that the generation of QMs from Mannich base precursors is a highly efficient process under physiological conditions,^[41]^ suggesting their potential applications as alkylating and cross‐linking agents. In this regard, Freccero's group reported photochemical generation of QMs from naphthol,^[34]^ 1,1‐bi‐2‐naphthol (BINOL),[^34^, ^42^, ^43^] and bis‐pirydyl derivatives.^[35]^ A crucial requirement for biocompatibility and application in live cell study is the wavelength of activation. Successful candidates should ideally react by visible light activation to minimise the risk of unwanted and unspecific photo‐induced damages such as thymine dimerization in double stranded DNA.^[44]^ To fulfil this requirement, Doria *et al*. successfully employed electronic conjugation as a valuable strategy to redshift the absorption spectra of the QM precursors.^[45]^ Based on these results, we aimed to expand the toolbox of photoactivatable covalent G‐quadruplex ligands. In this study, we present three novel classes of water‐soluble extended aromatic G4 ligands characterized by a planar V‐shaped rigid scaffold, bearing two QM precursors (QMPs **1**–**6** in Scheme 1), activatable at 310 and 365 nm. The simultaneous presence of two photoreactive moieties should ensure enhanced reactivity due to the higher cross‐section, without affecting the chromophore quantum yield, and limit off‐target migration thanks to the formation of two covalent bonds.^[46]^

Herein, we describe the synthesis of the new family of G4 ligands, report the investigation of the photoreactivity in aqueous solution, the systematic study of their biophysical G4 binding properties, and the assessment of their in vitro alkylating properties towards G4 structures by biochemical assays and MALDI‐ToF mass spectrometry analysis.

## Results

### Synthesis of the new family of QM precursors

**Scheme 2:**
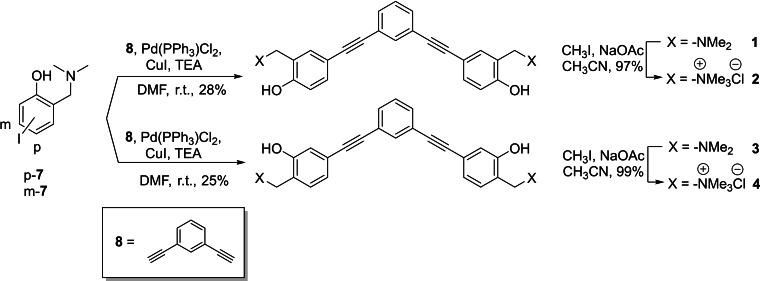
Synthesis of V‐shaped bifunctional *o*‐QMPs **1**–**4**.

The synthesis of V‐shaped bifunctional *ortho* and *meta* QMPs **1**–**4** followed a protocol previously published^[45]^ based on a two‐step synthesis according to Scheme 2. Starting from iodo‐derivatives **p‐7** and **m‐7**, we performed a Sonogashira cross‐coupling in DMF with the commercially available 1,3‐diethynylbenzene (**8**) to afford the desired compounds **1** and **3**. High catalyst loading [up to 10 % of PdCl_2_(PPh_3_)_2_] was required and the low isolated yields reflect the low substrate reactivity and catalyst turnover number. At last, exhaustive methylation of the tertiary amines **1** and **3** was achieved with an excess of iodomethane in CH_3_CN, providing the quaternary ammonium salts **2** and **4**.

**Scheme 3:**
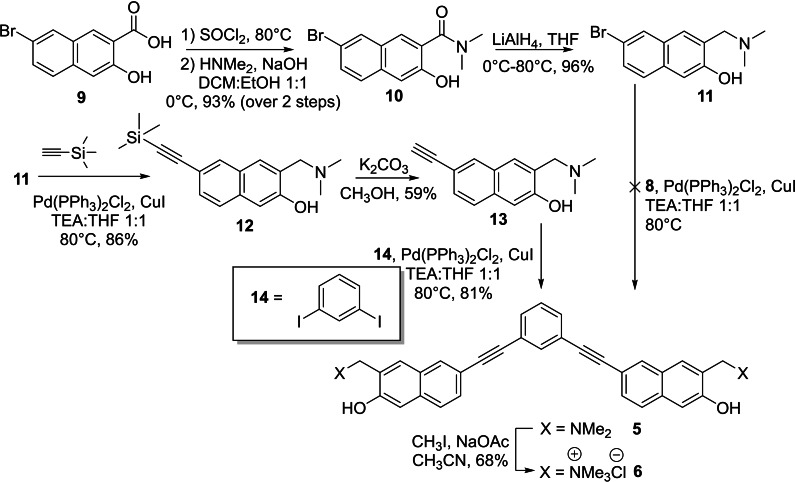
Synthesis of V‐shaped bifunctional QMPs **5** and **6** with a 1,3‐bis(naphthalen‐2‐ylethynyl)benzene structure.

The synthesis of the V‐shaped bifunctional naphthalene‐QMPs followed a different synthetic approach (Scheme 3). In fact, any attempt to obtain **5** through a direct cross‐coupling between the bromo‐derivative **11** and 1,3‐diethynylbenzene (**8**) was unsuccessful, yielding the starting materials. Substrate **11** was synthesized according to a five‐step synthesis starting from the naphthoic acid **9**,^[47]^ which was converted into the dimethylamide derivative **10** through the corresponding acyl chloride, followed by nucleophilic acyl substitution with dimethylamine solution. Subsequent reduction of compound **10** with lithium aluminium hydride afforded brominated Mannich base **11** in almost quantitative yield. Sonogashira cross‐coupling with ethynyltrimethylsilane, yielded arylethynyltrimethylsilane **12** in 59 % yield. Subsequently, trimethylsilyl cleavage by basic methanol and a second palladium‐mediated coupling with highly reactive 1,3‐diiodobenzene (**14**), afforded the desired product **5**, in excellent yield. Finally, the quaternary ammonium salt **6** was obtained by addition of an excess of iodomethane in CH_3_CN. Structures and purity of the synthesized compounds were confirmed by ^1^H and ^13^C NMR spectroscopy. In addition, the purity of compounds **1**–**6** was confirmed to be at least 95 % by high pressure liquid chromatography analysis (Supporting Information HPLC purity data).

### Absorption properties and photoreactivity

At first, we examined the spectroscopic properties of these ligands, which are affected by the alkyne moieties conjugation. Absorption profiles of compounds **1**–**6** were recorded both in water and in buffered solution at pH 7.2 (KCl 100 mM, Li Caco 10 mM) (Figure S1). 4‐ and 5‐arylethynyl derivatives **1**–**4**, characterized by the presence of phenyl moieties, showed a maximum peak centered at 290 and 299 nm in water and at 290 and 320 nm in buffer, which is in line with what has been observed for the prototype mono‐functional systems.^[45]^ Conversely, compounds **5** and **6**, equipped with naphthyl units, showed a red shift of the absorption maxima to *λ*_max_=325 nm, with a 100 nm tail (Figure S1 and Table S1). It should be highlighted that, at physiological pH, the absorption tails of derivatives **1**, **2**, **5**, and **6**, were located at longer wavelengths due to a partial deprotonation of the hydroxyl group. On the contrary, *m*‐phenylethynyl **3** and **4** absorption profiles were not significantly red‐shifted in buffer solution (Figure S1 and Table S1).

**Scheme 4:**
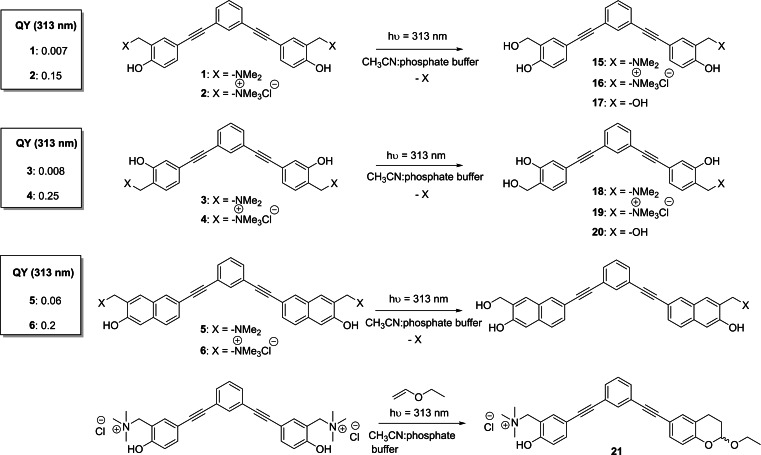
QMP photoreactivity and related quantum yields (QYs).

Subsequently, we thoroughly examined the photochemical behavior of ligands **1**–**6** under irradiation at 313 nm. As previous work demonstrated, excitation of these scaffolds results in the generation of QM intermediates.^[37]^ Due to their electrophilic nature, QMs can react with different types of nucleophiles such as amines, thiols, and water. Therefore, in order to investigate the efficiency of the photochemical reaction and to prove the formation of the expected QM, we studied the photoreactivity of compounds in water solution, by determining the quantum yields (*Φ*_R_) for each compound (Scheme 4). In detail, we analyzed substrate consumption, during the photo‐hydrolysis reaction, upon irradiation at 313 nm, in 1 : 1 CH_3_CN:buffered solution at pH=7.4. For all the arylethynyl derivatives, the experiments have been carried out with a high‐pressure mercury lamp previously calibrated by ferrioxalate actinometry.^[48]^ Irradiation of **1**, **3**, and **5** (10^−4^ M) resulted in poor quantum yields, indeed substrate consumptions remained low even after 30 min of irradiation. Conversely, in accordance with our expectations, quaternary ammonium salts **2**, **4** and **6** showed higher photoreactivity and resulted in complete substrate consumption (84 % of conversion) in 9 min. As previously reported, the presence of a good leaving group enhances the reactivity of these compounds,^[41]^ showing a significant increase in the photolysis quantum yields (Scheme 4).

Subsequently, to further confirm the results obtained in previous experiments, we isolated and fully characterized the products of photo‐hydration. In accordance with quantum yield measurements, irradiation at 313 nm of Mannich bases **1** and **3** resulted in low photoreactivity, even after long irradiation times (up to 90 min), and poor conversion. The desired mono‐hydration adducts **15** and **18** (Scheme 4), were obtained in traces and their isolation was not possible; nevertheless, their formation was detected by LC‐MS analysis (Supporting Information).

On the contrary, ammonium salts **2** and **4** showed the expected behavior with almost complete QMPs consumption (∼90 %) reached after only 30 min of irradiation. In this case, we isolated the mono‐hydration adducts **16** and **19** in 62 % and 43 % yields (Scheme 4), and the bis‐hydration products **17** and **20** in 8 % and 5 % yields, respectively (Scheme 4), confirming that both QMP **2** and **4** might also act as bifunctional photoactivated electrophiles. Naphthalene derivatives **5** and **6** displayed a different behavior: due to low quantum yield, product formation from precursor **5** was not detected even upon long irradiation times. On the contrary, irradiation of ammonium salt **6** produced fast degradation of the substrate and generation of a complex reaction mixture, not containing the expected photoproduct. To further validate QM generation, we have performed a trapping experiment on the most reactive QMP **2**, in the presence of ethyl vinyl ether (EVE) as dienophile. In detail, we have irradiated **2** with EVE in a 1 : 1 CH_3_CN:PBS solution, yielding the Hetero Diels‐Alder cycloaddition adduct in 60 % yield (Supporting Information).

### LFP studies

**Figure 1:**
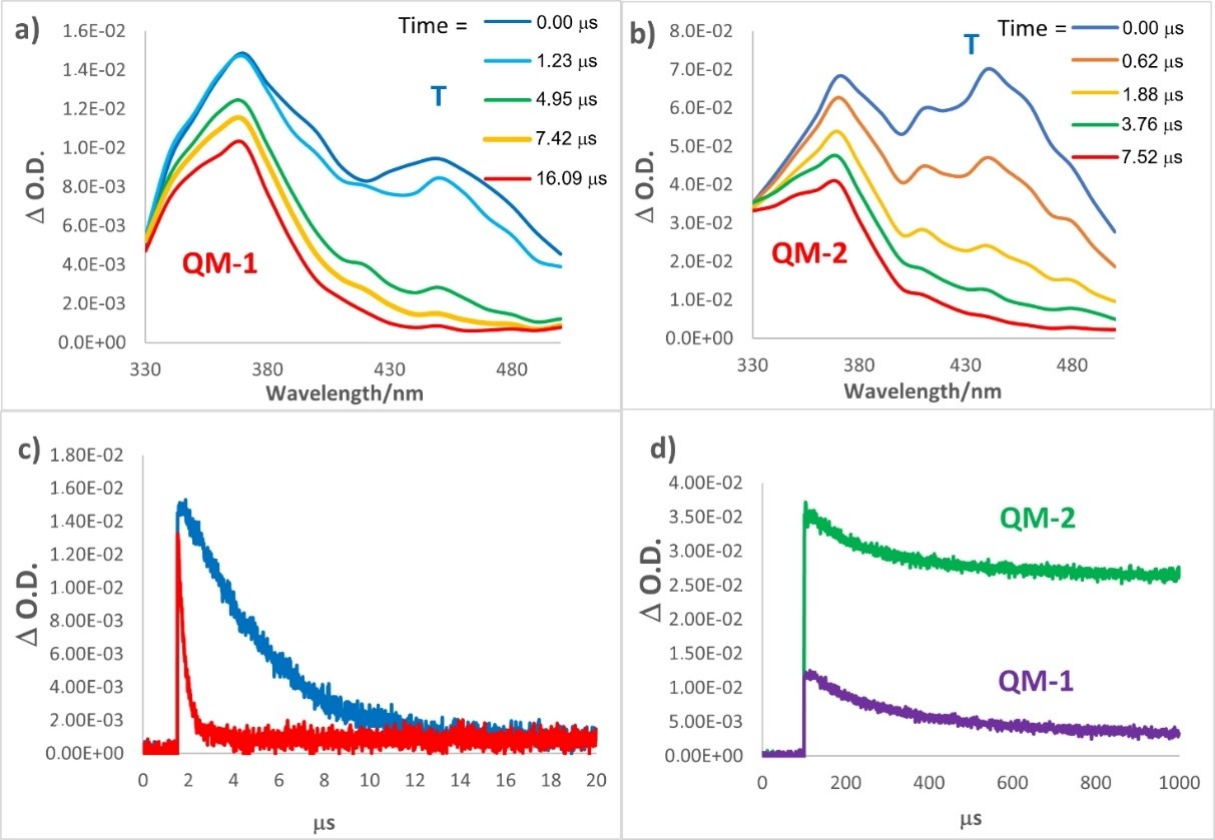
Transient absorption spectra of a) **1** and b) **2** 10^−4^ M CH_3_CN solutions, purged with Ar, irradiated at 266 nm by LFP. c) Decay traces monitored at 470 nm of CH_3_CN solutions of **1** in presence of Ar (blue line) or O_2_ (red line); d) decay traces of solutions of **1** (violet line) and **2** (green line) monitored at 370 nm, in the presence of O_2_.

In order to further corroborate the photogeneration of QMs as reactive intermediates, laser flash photolysis (FLP) studies were performed for QMPs **1**–**4**. Analyses were conducted in 1×10^−4^ M CH_3_CN and aqueous CH_3_CN (1 : 1) solutions of QMPs, irradiated with Nd:YAG laser at 266 nm. The analyses were performed in neat and aqueous CH_3_CN solutions, purged with argon or oxygen. In general, all analysed compounds presented similar behaviour, therefore, we focus our discussion on the Mannich base **1** and the corresponding ammonium salt **2**, chosen as models. In CH_3_CN solution purged with Ar, we observed a transient species with absorption maximum at 450 nm (Figure 1a and b, blue line), which was effectively quenched by O_2_ addition (Figures 1c and S2), for both compounds **1** and **2**. Similar observations have already been reported for phenol and naphthol derivatives and can be attributed to triplet‐triplet transient (T‐T) absorption.[^45^, ^49^] Besides the fast‐decaying triplets in the transient spectra in CH_3_CN solution purged with Ar, we observed the parallel generation of an additional transient species, for both **1** and **2**, becoming the only detectable transient after 10 μs (Figure 1a and b, red line). These species exhibit an absorption centred at 370 nm, and a lifetime longer that 1 ms (Figure 1d). Moreover, its lifetime was not affected by O_2_, a key evidence that allowed the assignment of this long‐living species to the expected quinone methide, coherently with what is reported in literature.^[45]^ As shown in Figure 1(b), the quaternary ammonium salt **2** generated a transient absorption maximum at 370 nm similar to what observed for Mannich base **1**. However, the decay traces of **QM‐2** monitored at 370 nm, in CH_3_CN solution with O_2_ (Figure 1d), showed a clear different intensity as compared to **QM‐1**. The intensity of the signal immediately after the laser pulse is at least 3‐times more intense than the signal derived from **QM‐2**, confirming that the ammonium salt **2** is a much more efficient QMP than its Mannich base **1**, as confirmed from steady state studies previously described.

The differences recorded for compounds **3** and **4** are related to the maximum absorption wavelengths and intensity of the QM species. Indeed, we observed a blue‐shift of the absorption band of the transient QM, compared to the homologue **1** and **2**, with a maximum centered to 330 nm (Figure S3). Moreover, coherently with previous experiments, the intensity of QM signal was higher for the ammonium salt but, in general, **3** and **4** were characterized by lower QM generation efficiency compared to **1** and **2** (Figure S3). Finally, we decided to run the very same LFP irradiation experiments in aqueous CH_3_CN (1 : 1). In fact, protic conditions should favour the selective photogeneration of the QM intermediate, depleting the population of the triplet excited state.^[45]^ As expected, water addition to CH_3_CN remarkably reduced the triplet (T) generation efficiency of both compounds **1** and **2** (absorption at 435 and 410–430 nm, respectively; Figure S4a and b), without significant effects on QM generation (330–370 nm).

### Biophysical studies

#### FRET melting assay

**Figure 2:**
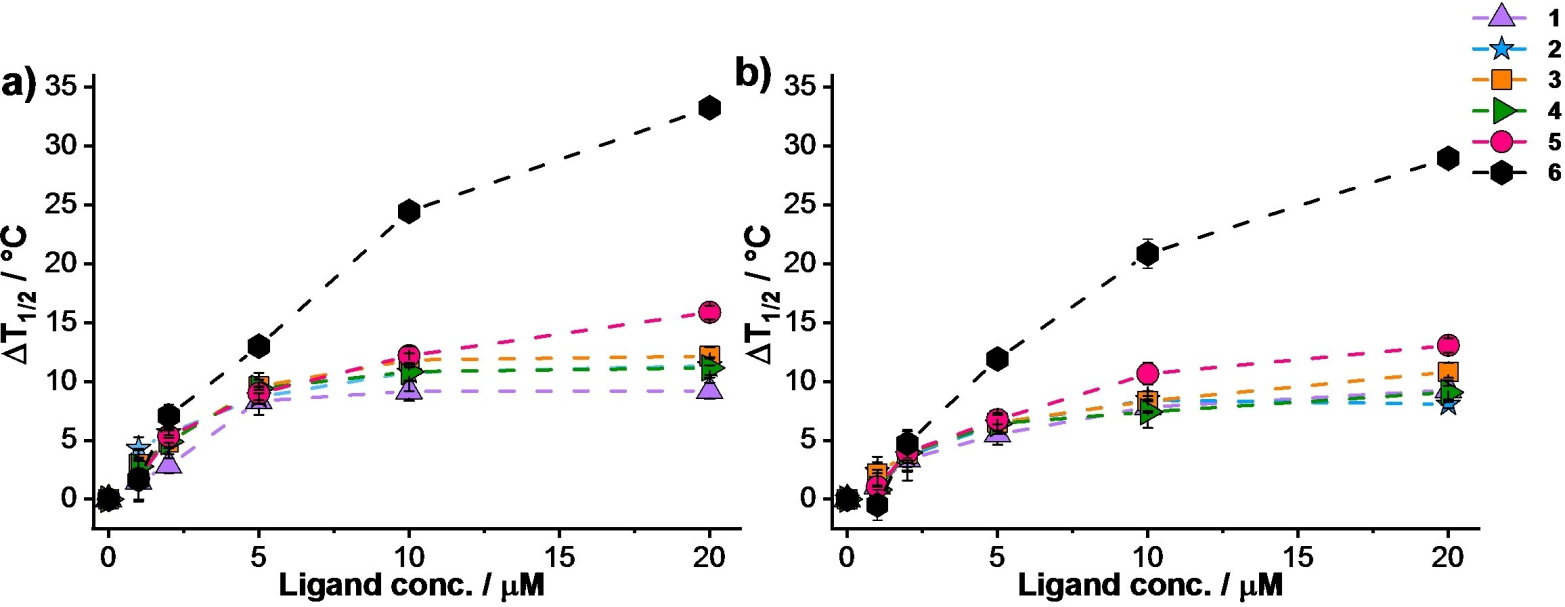
Quantitative Δ*T*_1/2_ dependence of a) F21T and b) F(Pu24T)T as a function of putative ligand concentrations. Conditions: a) DNA 0.2 μM, Li Caco 10 mM (pH 7.2), LiCl 90 mM and KCl 10 mM; b) DNA 0.2 μM, Li Caco 10 mM (pH 7.2), LiCl 99 mM and KCl 1 mM. Compounds **1**–**6** were employed at concentration ranging between 0 μM and 20 μM.

The ability of the synthesized molecules to bind and stabilize G‐quadruplex structures was investigated by FRET melting assay.^[50]^ The latter exploits doubly labelled single‐stranded DNAs chosen as prototype of G4 structures. Briefly, this assay allows the measure of the stabilization induced by a ligand on a G4 structure, analysing the difference in melting temperature in the presence and in the absence of ligand. At first, we confirmed compound **1**–**6** interaction with G‐quadruplex structures by performing FRET experiments in the presence of a polymorphic sequence, mimicking the human telomeric sequence F21T,[^51^, ^52^, ^53^] and a parallel forming sequence, mimicking the c‐myc proto‐oncogene promoter F(Pu24T)T,^[54]^ with ligand concentration up to 20 μM (100 mol equiv.). A clear dose‐response correlation emerges when the Δ*T*_1/2_ values are plotted as a function of ligand concentration (Figure 2). At 2 μM ligand concentration (10 mol equiv.), the derivatives did not show significant difference in Δ*T*_1/2_ values in the presence of the same G4 structure. When the ligand concentration was gradually increased, a distinct trend could be observed. Naphthyl‐derivatives **5** and **6** acted as better ligands compared to the benzo‐derivatives, and between the two, the ammonium salt **6** was the best performing at 20 μM, Δ*T*_1/2_=33.3 °C and 29.0 °C in the presence of F21T and F(Pu24T)T, respectively. Differently, bifunctional benzo‐derivatives **1**–**4** showed very similar stabilization properties; at 20 μM they displayed Δ*T*_1/2_ values ranging between 8.0 and 12.1 °C in the presence of both G4 structures.

**Figure 3:**
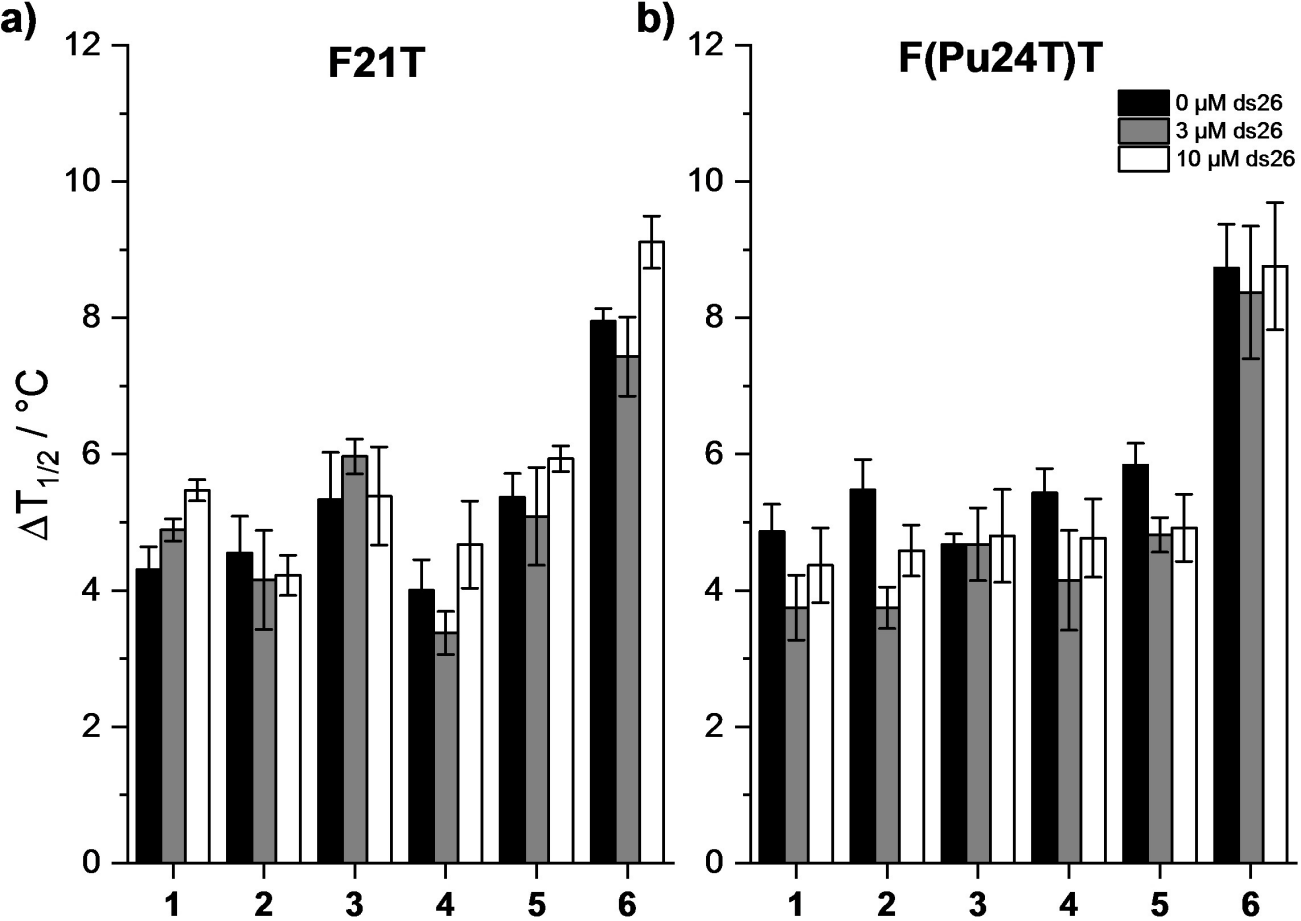
FRET‐melting competition assay with a) F21T and b) F(Pu24T)T in the absence and in the presence of double‐stranded DNA competitor (ds26). Conditions: F21T or F(Pu24T)T 0.2 μM, compounds 2 μM, ds26 0, 3, or 10 μM (black, dark gray and white bars, respectively) (a) Li Caco 10 mM (pH 7.2), LiCl 90 mM and KCl 10 mM and (b) Li Caco 10 mM (pH 7.2), LiCl 99 mM and KCl 1 mM. Analyzed compounds **1–6**.

After assessing the ability of all the newly synthesized ligands to bind the two aforementioned G4 structures, we challenged their selectivity over duplex DNA, the more abundant DNA structure present in a biological environment. FRET‐based competition assays were performed in the presence of F21T and F(Pu24T)T with increasing concentration of a non‐fluorescently labelled double‐stranded DNA (ds26) competitor. Due to the general low stabilization observed, competition experiments were conducted in the presence of 2 μM (10 mol equiv.) of G4 ligands. Notably, all the compounds employed in this study displayed high selectivity for the tested G4s, as Δ*T*_1/2_ was poorly affected by the addition of the DNA competitor (Figure 3).

**Figure 4:**
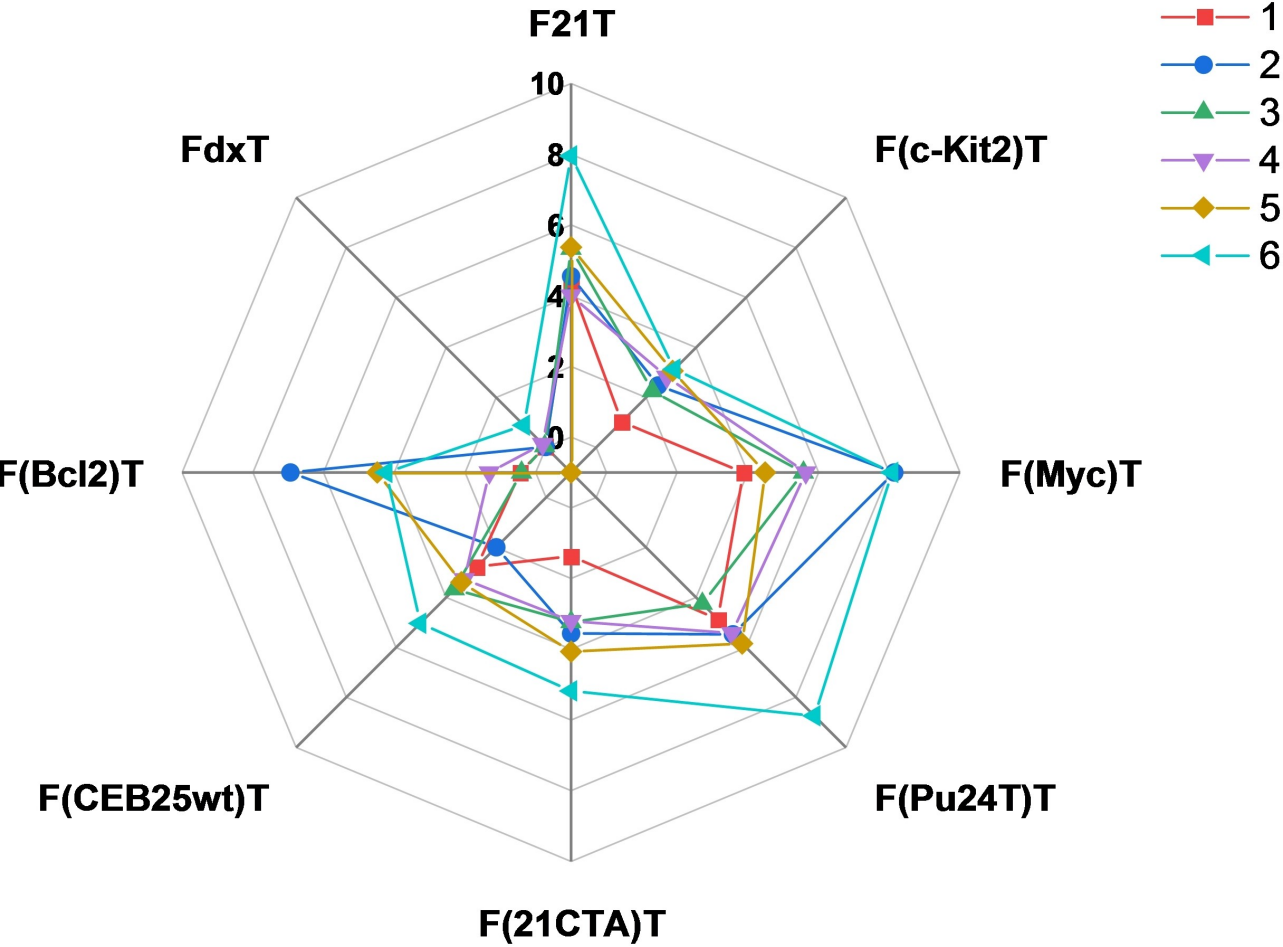
Quantitative analysis of FRET‐melting experiments in the presence of F21T, F(c‐kit2)T, F(Myc)T, F(Pu24T)T, F(21CTA)T, F(CEB25wt)T, F(Bcl2)T and FdxT (0.2 μM) represented with a radar graph. Stabilization in Li Caco 10 mM (pH 7.2), LiCl 99 mM and KCl 1 mM for F(c‐kit2)T, F(Myc)T, F(Pu24T)T, F(CEB25wt)T and F(Bcl2)T and in Li Caco 10 mM (pH 7.2), LiCl 90 mM and KCl 10 mM for F21T, F(21CTA)T and FdxT is indicated for analysed compounds **1**–**6** (2 μM).

Compounds **1**–**6** (2 μM) were tested in the presence of a panel of G4‐forming sequences (0.2 μM) able to form G4 structures with different topologies: the aforementioned human telomeric sequence (F21T);[^51^, ^52^, ^53^] parallel forming sequences differing in the central loop size such as c‐myc proto‐oncogene promoter (FMycT^[55]^ and F(Pu24T)T^[54]^), c‐kit2 proto‐oncogene promoter (Fkit2T),^[56]^ human minisatellite repeat native sequence (FCEB25wtT),^[57]^ Bcl2 proto‐oncogene promoter (FBcl2T),^[58]^ and an antiparallel forming sequence from the human telomeric sequence variant (F21CTAT).^[59]^ We also included as a negative control FdxT, a hairpin duplex with a hexaethyleneglycol (HEG) loop. These experiments identified compound **6** as the best G4 stabilizer. We observed F21T, F(Myc)T and F(Pu24T)T were found to be the sequences most stabilized by compound **6**, with Δ*T*_1/2_ values never exceeding 8.7 °C, whereas F(c‐Kit2)T, F(21CTA)T and F(CEB25wt)T showed the smallest stabilization of the selected G4s (Figure 4). Importantly, no stabilization was observed in the presence of the duplex control FdxT. Overall, only small differences in Δ*T*_1/2_ values were found among the six compounds, suggesting that the naphthyl moieties do not add relevant interactions between the ligand and the G4 target such as to improve the stabilization properties.

#### Circular dichroism (CD) spectroscopy

**Figure 5:**
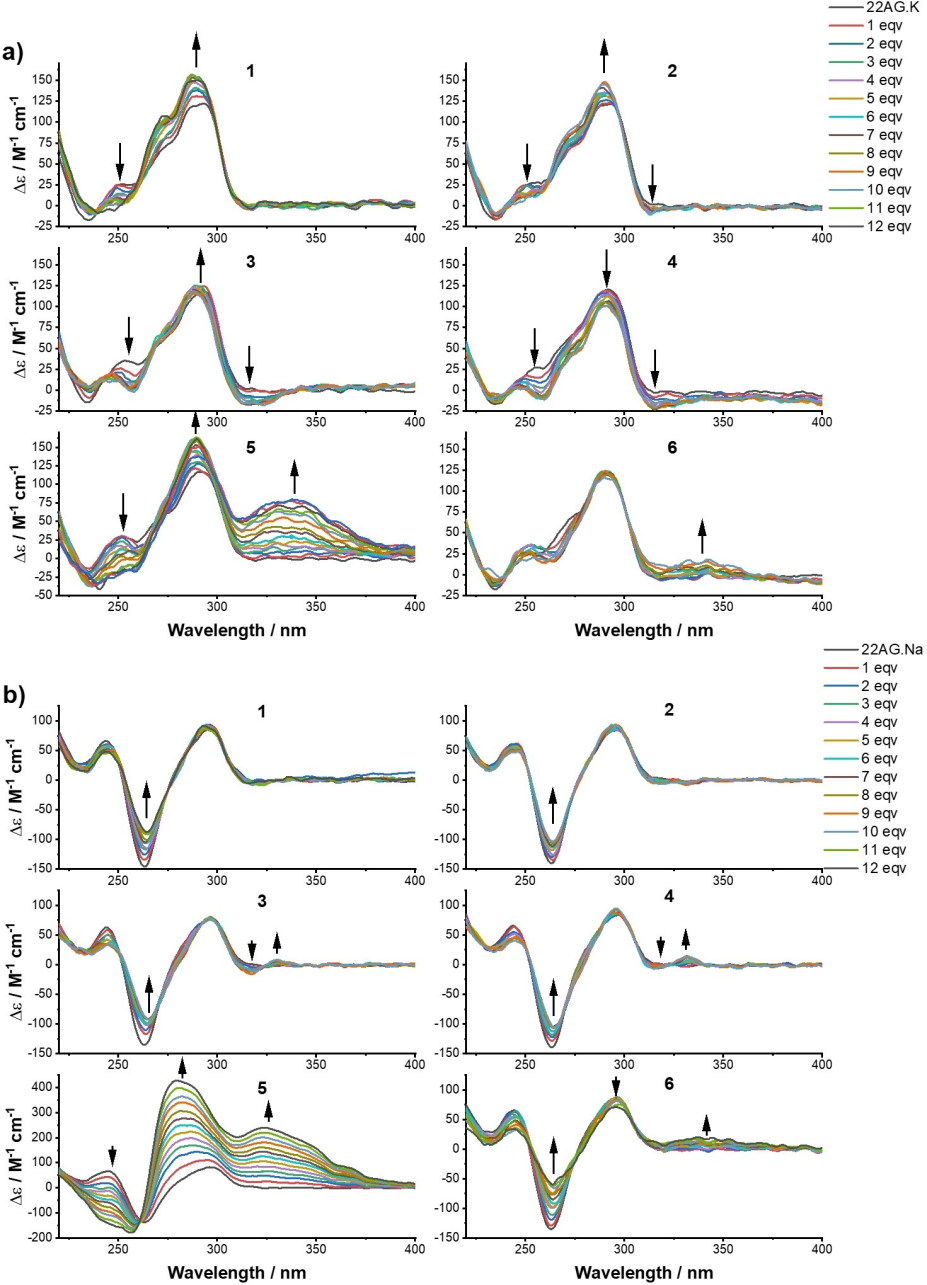
Change in the CD spectra of 22AG (3 μM) upon addition of increasing concentration of ligands **1**–**6**: a) in 10 mM Li Caco (pH 7.2), 100 mM KCl, and b) in 10 mM Li Caco (pH 7.2), 100 mM NaCl. Titration experiments for compounds **2**–**4** and **6** (only in K^+^‐rich buffer for the latter) were stopped after the addition of 10 eqv. of ligand.

In the attempt to gain insight into the interaction of the putative ligands with G‐quadruplex DNAs, we conducted CD titrations to determine affinity constants. The titrations were carried on until not further changes were observed in the CD spectra, suggesting DNA saturation point was reached. Analyzed compounds did not display any dichroic signal in their absorption region (Figure S5). The dichroic signature of the prefolded human telomeric sequence (22AG) in K‐rich buffer was consistent with data previously reported in literature for the same sequence, characterized by a peak at 293 nm, a shoulder around 265 nm, and a trough at 240 nm, corresponding to a hybrid [3+1] structure.[^60^, ^61^, ^62^] Upon addition of the ligands, changes in the G‐quadruplex structure conformation were observed as a result of the binding events. Compounds **1**–**3** and **5** (Figure 5a) showed a common trend: an increase of the peak at 293 nm combined to a hypsochromic shift of 3 nm together with the conversion of the positive shoulder to a negative trough at 265 nm. Differently, compound **4** showed a decrease ellipticity at both wavelengths and almost no variation was observed for compound **6**. The presence of neat isoelliptic points (Table S2) in the CD spectra of compounds **1**–**3** indicates a two‐state model, where a single equilibrium converts the free DNA structure into a bound species and no other bound topologies are involved. Conversely, derivatives **4**–**6** exhibited non‐neat isoelliptic points letting us infer about the coexistence of more than two bound structures. All compounds, except **1**, showed a change in ellipticity monitored between 320–400 nm, which is remarkable for **5**. This has to be assigned to induced CD (ICD) of the ligand caused by the sensing of the chiral environment (Figure 5a). The same experiments were performed in Na‐rich buffer, where 22AG presents the characteristic signature of an antiparallel topology: a peak at 295 nm, one at 245 nm and a trough at 260 nm.[^63^, ^64^] Upon addition of derivatives **1**–**4** and **6** an increase of the ellipticity at 260 nm was detected, together with a 2 nm bathochromic shift (Figure 5b). Several neat isoelliptic points (Table S2) were identified during the titration experiments suggesting also in this case, a two‐state equilibrium between the free form and the bound form. In these conditions, an ICD signal beyond 320 nm was observed for compounds **3–6**. An unexpected behaviour was observed for compound **5**: a constant increase of the ellipticity at both 293 nm – matched with a hypsochromic shift of 5 nm – and at 330 nm was detected and signal saturation was not reached even after addition of 12 mol equiv. of ligand. CD titration spectra of each compound were used to determine the dissociation constants, by employing a non‐linear regression based on Hill equation to obtain a global fitting. For all compounds, curve fitting highlighted a positive cooperative binding of these ligands towards the telomeric sequence (Figures S6–S11). Dissociation constants on the micromolar range were obtained for all the ligands either in K‐ and Na‐rich buffer (Table S3). Due to the unusual response of **5** in Na‐rich buffer, we were not able to determine the binding constant for this ligand in these conditions.

### Photochemical reactions on G‐quadruplex DNA

**Figure 6:**
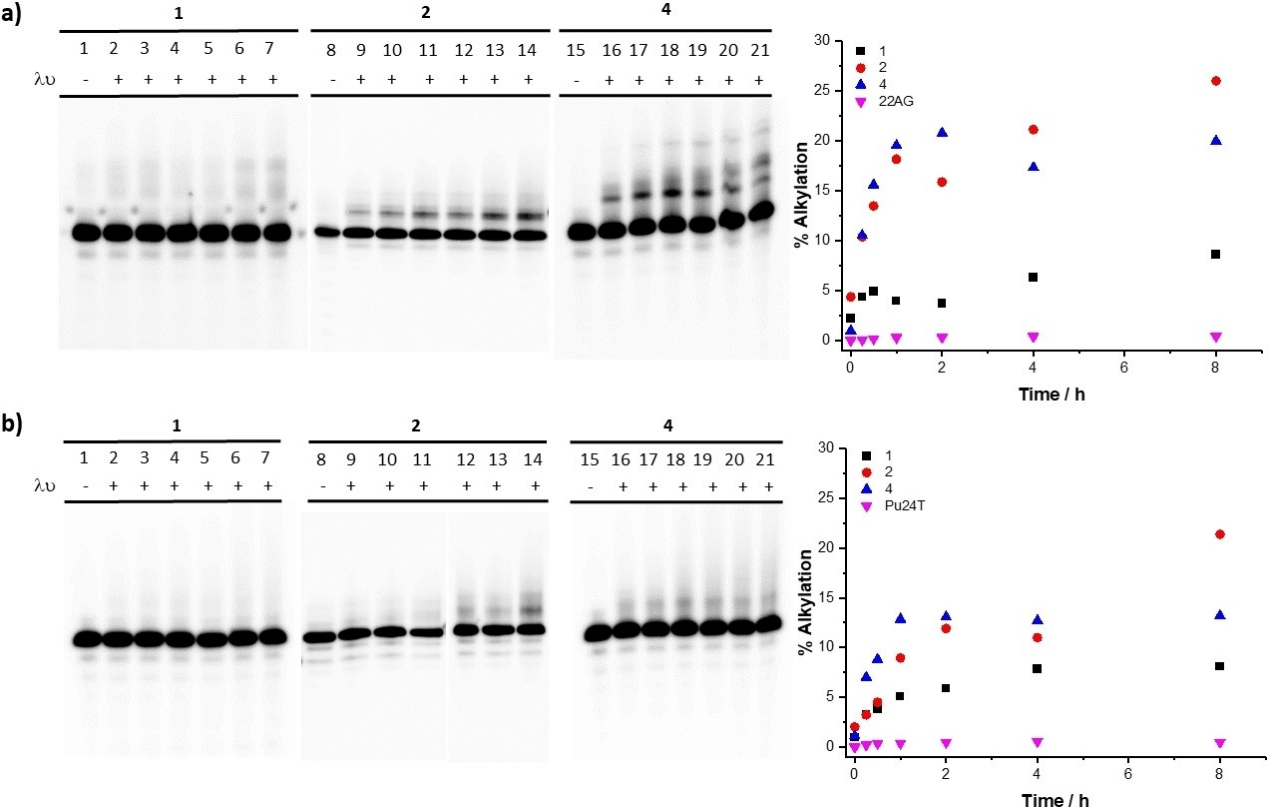
Denaturing gel electrophoresis (15 % acrylamide) of the alkylation products of a) 22AG and b) Pu24T (10 μM) in the presence of K^+^ buffer (10 mM Li Caco (pH 7.2), 100 mM KCl) with **1**, **2**, and **4** (20 μM) at increasing irradiation time (0, 0.25, 0.5, 1, 2, 4, and 8 h). Lanes 1, 8, and 15 are control experiments showing the effect in the absence of irradiation with a) 22AG and b) Pu24T. Lanes 2–7 show the results of irradiation of 22AG and Pu24T at 365 nm in the presence of 2 equiv. of **1**, lanes 9–14 show the results of irradiation of 22AG and Pu24T at 365 nm in the presence of 2 equiv. of **2**, and lanes 16–21 show the results of irradiation of 22AG and Pu24T at 365 nm in the presence of 2 equiv. of **4** during 0.25, 0.5, 1, 2, 4, and 8 h. On the right, quantitative analysis of the alkylation of a) the telomeric sequence 22AG and b) Pu24T oncogene (10 μM) at increasing irradiation time (0, 0.25, 0.5, 1, 2, 4, and 8 h) in the presence of **1**, **2**, and **4** (20 μM) or in the absence of ligand (22AG and Pu24T).

Next, we assessed the ability of QMPs **1**–**4** to form covalent adducts within G‐quadruplex DNA. Besides the absorption maxima located around 310 nm, **1**–**4** feature an absorption band extending up to 370 nm in buffer solution. Based on the absorption spectra, we decided to carry out the irradiation experiments with a collimated light beam centered at 365 (±15) nm, in the presence of two G‐quadruplex sequences chosen as prototype, 22AG and Pu24T. The selected wavelength of irradiation represents a compromise between biocompatibility and absorption properties of the compounds: indeed, despite the maximum absorbance is recorded among 290 and 325 nm, we decided to take advantage of their red‐shifted absorption tails (ranging from 327 to 410 nm) to minimize potential direct DNA damages. At first, to characterize compound reactivity as a function of the G‐quadruplex target, we conducted photochemical reactions by varying ligand concentration and irradiation time in the presence of 22AG and Pu24T. Analysis of the mixtures by denaturing gel electrophoresis showed the generation of retarded bands attributed to the alkylated G4 DNA, considering that the photoproducts provide additional positive charges and mass that slow down the migration. We observed that only **1**, **2**, and **4** were able to alkylate 22AG and Pu24T (Figure 6). In all experimental conditions we could remark the complete absence of alkylation of compound **3** (data not shown). Alkylation efficiency was assessed as a function of the irradiation time for both 22AG and Pu24T (Figures 6 and S12). For all compounds, the alkylation yields increased with the irradiation time before reaching saturation after 2 h. Under the same reaction conditions, the three ligands showed sluggish reactivity towards the snap‐back parallel Pu24T^[54]^ as compared to the hybrid 22AG.[^51^, ^52^, ^53^] Although different alkylation efficiencies were displayed by the ligands on the two G4 structures, a trend could be detected: **2** and **4** displayed the highest alkylation yields (26 %–19 % with 22AG, 21.4 %–13.2 % with Pu24T) and **1** showed lower reactivity (8.6 % with 22AG, 8.1 % with Pu24T) (Figure 6). These results are in accordance with the photochemical studies: higher alkylation efficiency was observed with derivatives **2** and **4**, which displayed not only higher quantum yields, but also formed the corresponding bis‐ and mono‐adducts with water in significant amount (Scheme 4). Conversely, the absence of alkylation observed with **3** can be attributed to both absence of absorption at the activation wavelength and low photolysis efficiency. These experimental data also highlight that the compound alkylation ability depends on the nature of the aromatic ring bearing the QMP. This suggests that the orientation of the reactive QM, determined by the binding of the derivate to the G4 target, strongly affects alkylation efficiency. Therefore, the spatial proximity between the arylethynyl benzo‐QM reactive sites and the nucleotide residues of the G4 structure guarantees the success of the alkylation. The low reactivity observed with Pu24T may reflect a less efficient QM trapping by proximal nucleobases due to high compactness of this structure, which displays three short propeller loops and one snap‐back loop on the 3’‐quartet, possibly making them more difficult to access.

**Figure 7:**
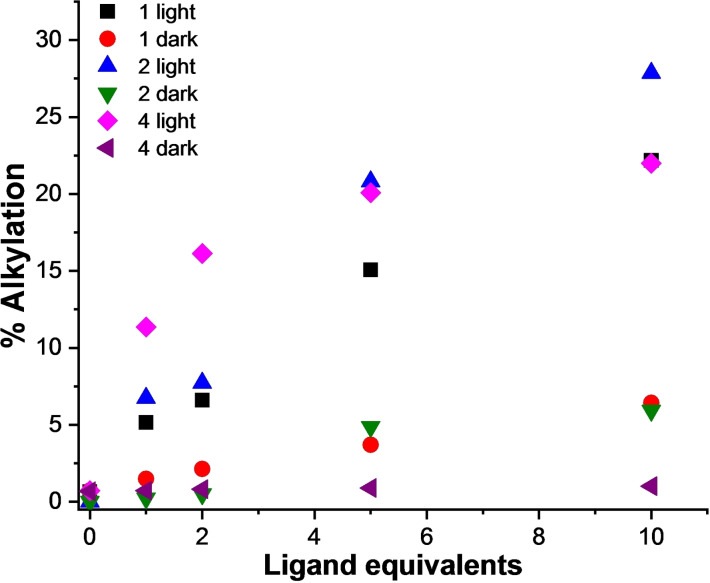
Quantitative analysis of the alkylation of the telomeric sequence 22AG (10 μM, 10 mM Li Caco (pH 7.2), 100 mM KCl) by **1**, **2**, and **4** (10, 20, 50, and 100 μM) in the presence and in the absence of irradiation.

Dose‐dependent experiments in the presence of 22AG in K‐rich buffer showed highest alkylation yields at higher ligand concentrations (from 1 to 10 mol equiv.) after 60 min of irradiation: Mannich base **1** (5.1 %–22.1 %, Figures 7 and S13), and ammonium salts **2** (6.7 %–27.9 %, Figures 7 and S14) and **4** (11.3 %–22.0 %, Figures 7 and S15). Quantification analysis highlighted a mild thermal reactivity of the two 4‐arylethynyl derivatives (**1** and **2**) (Figures 7, S13 and S14) at higher ligand concentrations (10 mol equiv. ∼5%). Ligand/G4 DNA 2 : 1 ratio gave good photochemical yields combined to clean reaction mixtures and negligible thermal reactivity; therefore, these conditions were applied throughout the study.

**Figure 8:**
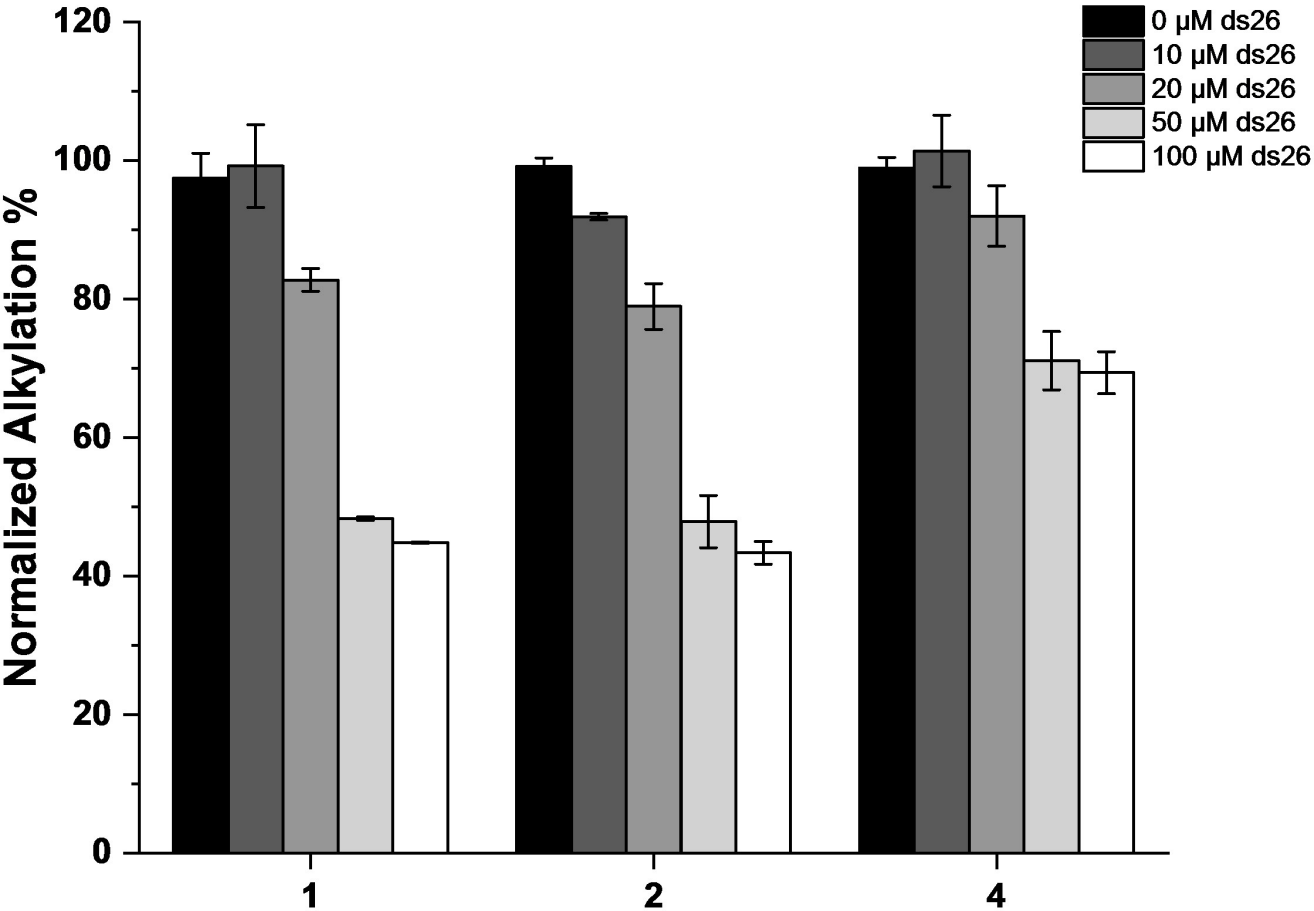
Quantitative analysis of the alkylation of the human telomeric sequence 22AG (10 μM, 10 mM Li Caco (pH 7.2), 100 mM KCl) by **1**, **2**, and **4** (20 μM) in the absence (black bars) or in the presence of ds26 competitor (10, 20, 50, and 100 μM) (from dark gray to white bars).

To assess whether the formation of adducts with 22AG and Pu24T was selective for the G4 conformations, we reproduced the experiment with a control duplex oligonucleotide, constituted by 22AG and its C‐rich sequence. Negligible reactivity was observed in the presence of the duplex structure (Figure S16). Subsequently, to evaluate whether the photochemical trapping of the G4 structures was a DNA structure‐selective process, we conducted competition experiments in the presence of a large excess of non‐labelled duplex DNA (ds26) (up to 10 mol equiv.) and 22AG, chosen as most efficiently trapped structure. Under these experimental conditions, 22AG alkylation was maintained with a moderate decrease in yield for **4** (0–30 %, Figures 8 and S17), whilst both **1** and **2** were more affected by the presence of the duplex DNA competitor (ca. 55 % decrease, Figures 8 and S17). These results indicate that the position of the alkylating moiety is highly important for selectivity, thereby suggesting the relevance of the regioisomer effect during generation of the covalent bond.

As a mean of identifying the alkylation sites, we isolated the alkylated adducts from the gel and subjected them to different sequencing protocols. None of the isolated alkylation products responded to classical alkaline treatment, which induces cleavage on the 5’ side of unstable alkylated nucleobases and would allow a direct determination of alkylation sites.^[24]^ The latter did not reveal any band generation that could be associated to a specific alkali‐labile alkylated base, regardless of the quinone methides precursor employed (data not shown). To overcome this problem, the alkylated oligonucleotides were digested with 3’‐exonuclease, which stops in proximity of alkylated sites.^[65]^ After digestion a strikingly different behavior could be observed for the three derivatives. Compound **2** showed partial thermic dealkylation, generating the starting DNA during the purification process (DNA that is consequently completely digested by the 3’‐exonuclease). Differently, the remaining alkylated product did not generate any digested fragments (Figure S18). This result suggests that **2** binds the 3’‐end of the 22AG structure impeding either the binding of the enzyme or the starting of the digestion process (Figure S18).^[66]^ Conversely, after 3’‐exonuclease treatment both compounds **1** and **4** generated several digested fragments, suggesting the presence of different alkylation products able to induce the enzymatic arrest (Figure S19). However, the presence of the ligand on the DNA fragments affected their migration on the gel preventing the identification of the exact alkylated base. Therefore, the alkylated fragments were then isolated from the gel and both thermal and photochemical dealkylation (data not shown) were applied to evaluate the possibility to revert the quinone methide generation and release the ligand from the 22AG fragments; however, all of the techniques employed failed. Sequencing methodologies missed to identify the alkylated sites on the human telomeric sequence, but allowed to locate compound **2** on the 3’‐end of 22AG.

### MALDI‐ToF mass spectrometry analysis

**Figure 9:**
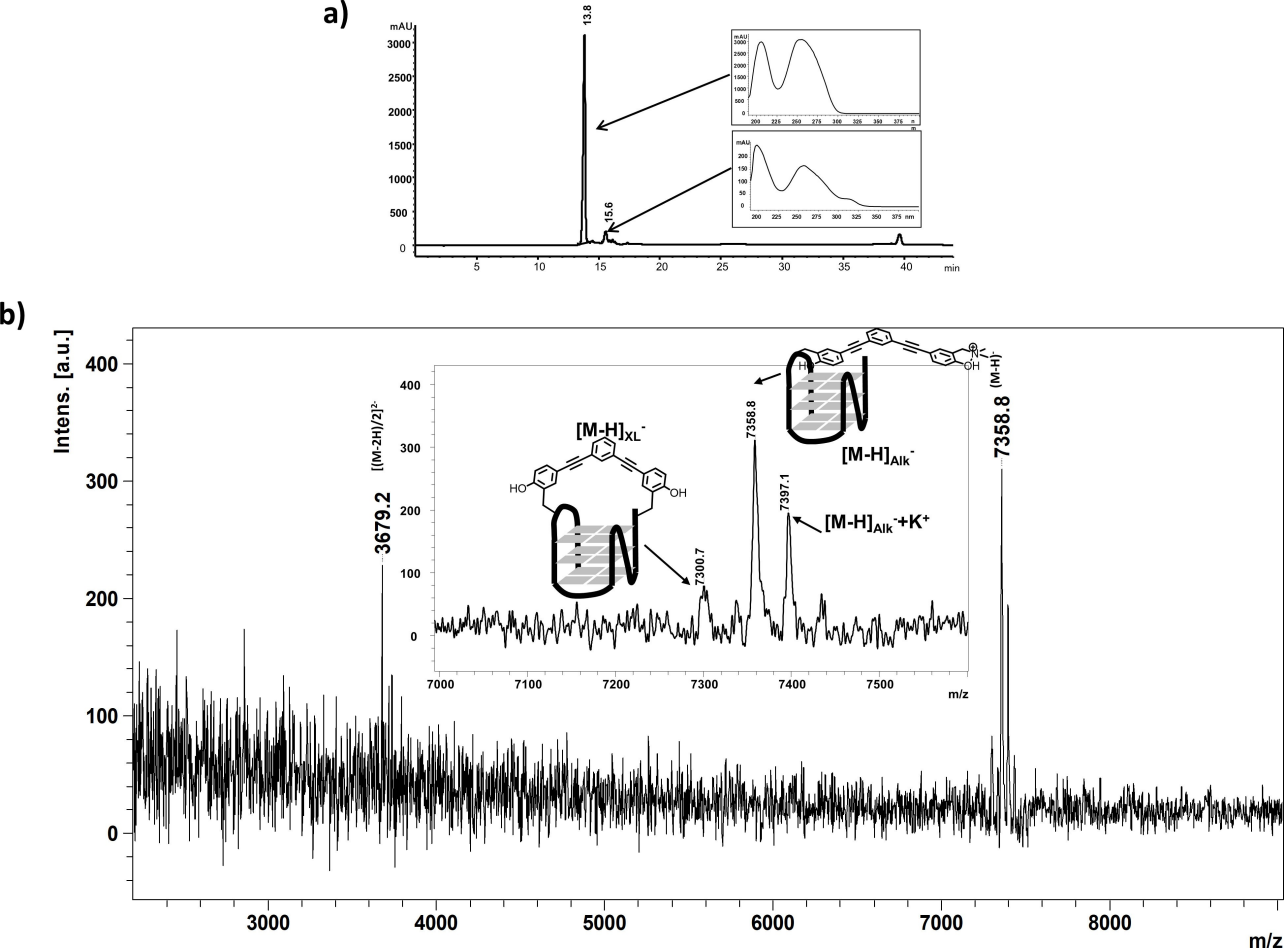
a) Chromatographic profile (recorded at 260 nm) of the crude reaction mixture obtained for compound **2** using a gradient from 0 % to 50 % CH_3_CN in 10 mM TEAA buffer at 50 °C. Insets: UV spectra extracted for each chromatographic peak; the adduct‐containing DNA shows a specific absorbance at 320 nm. b) MALDI‐ToF mass spectrum of the collected product eluted at 15.6 min and schematic representation of the covalent products produced by photoactivation of compound **2** in the presence of 22AG. (Figure 9 has been slightly modified and has been re‐submitted as a separated .png file. We simply removed the square around Figure 9a and improve the Y axes to make it more readeble)

Since PAGE failed to give insight on the exact alkylated sites generated by ligands **1**, **2**, and **4** on 22AG, we decided to analyse the reaction mixtures produced by irradiation of the three QMPs (20 μM) in the presence of 22AG (10 μM) by Reverse Phase‐HPLC and MALDI‐ToF mass spectrometry. The chromatogram obtained from elution of the reaction mixture generated by photolysis of compound **1** with 22AG showed only the peak corresponding to the unmodified G4 sequence. Differently, chromatograms of the crude reaction mixtures obtained with compounds **2** (Figures 9a and S20) and **4** (Figure S21) respectively, showed the presence of additional broad peaks with higher retention times, which could be clearly associated to the alkylated products.

We characterized the nature of the alkylated products generated by compounds **2** and **4** by MALDI‐ToF MS analysis. The mass spectrum of the reaction products resulting from photolysis of 22AG and compound **2** showed two mono‐charged product peaks: one main product at [M−H]_Alk_^−^=7358.8 Da corresponding to the mono‐alkylated 22AG, and the other one with molecular mass [M−H]_XL_^−^=7300.7 Da corresponding to the cross‐linked product that lost the two trimethylammonium moieties (−57 Da each that give rise to the formation of two methine functions), in accordance with preliminary photochemical studies (Figure 9b). The same analysis was conducted for compound **4**. Beside the mono‐alkylated and the cross‐linked products, a third mono‐charged product peak was detected at [M−H]_Hyd_^−^=7317.9 Da corresponding to the mono‐alkylated 22AG where the second QM of **4** reacted with water to generate the hydrolysis product (Figure S22). In order to investigate the alkylation site on the G4 sequence, we performed MALDI‐ToF MS analysis of the digestion products of alkylated adducts of compound **2** generated by snake venom phosphodiesterase I (digestion from 3’‐end) and calf spleen phosphodiesterase II (digestion from 5’‐end).[^67^, ^68^] We focused our attention on the alkylated products of compound **2** due to the less complex reaction mixture, composed mainly by the unreacted 22AG and by the mono‐alkylated adduct. As previously observed from gel experiments, phosphodiesterase I is not able to start the digestion of the alkylated fragment, not even after 30 min, which is the time required for the full digestion of the unmodified 22AG. After 6 h of digestion, only the first guanine located at the 3’‐end of the G4 sequence was partially digested (Figures S23 and S24). Longer digestion time did not modify the result. Treatment with phosphodiesterase II showed a partial digestion of the alkylated fragment starting from the 5’‐end, however, a much slower digestion kinetic was observed suggesting that the presence of the ligand arrests the enzyme 5’‐exonuclease activity. Combined together, these data corroborated the results obtained by denaturing PAGE, which located the ligand in proximity of the 3’‐end of the G4 structure. The alkylation on the 3’‐end of 22AG blocks completely the exonuclease activity of phosphodiesterase I and significantly slows down phosphodiesterase II. Most likely, the presence of the ligand may partially keep folded the G4 inhibiting the exonuclease activity of phosphodiesterase II. MALDI‐ToF Mass Spectrometry analysis gave very consistent results to those one obtained by sequencing experiments.

## Conclusion

In summary, a new family of photoactivatable V‐shaped bifunctional quinone methide precursors, able to bind and alkylate G‐quadruplex structures, were synthesized and characterized. Their binding and alkylation properties were systematically investigated by various photochemical, biophysical, and biochemical techniques. Photochemical studies clearly highlighted the higher QM generation efficiency of ammonium salts **2** and **4** and their ability to form both mono‐ and bis‐hydrated products as compared to corresponding Mannich bases **1** and **3**. FRET melting assays and CD titrations validated the ability of the synthesized compounds to bind G4 structures with *K*_d_ values lying in the μM range. Furthermore, photoalkylating properties were assessed *in vitro* in the presence of 22AG and Pu24T used as prototypes of G4 structures, by photolysis at 365 nm. Notably, these experiments emphasized on the ability of compounds **1**, **2**, and **4** to alkylate both structures with a marked preference towards 22AG. Moreover, competition experiments, performed in presence of duplex DNA, pointed out that compound **4** has significantly higher selectivity suggesting an important contribution of the regioisomer effect during generation of the covalent bond. Despite the inability to identify the exact alkylation site, sequencing and MALDI‐ToF mass spectrometry analyses provided the identification of mono‐alkylation and cross‐linking products derived from both **2** and **4** in the presence of the human telomeric sequence. Given the strong and increasing interest for the development of alkylating molecular tools and considering the unique spatio‐temporal control guaranteed by light for this purpose, extension of this photoactivation‐driven approach to the design of new families of alkylating G4 ligands, using alternative aromatic cores and reactive intermediates, is currently under development.

## Conflict of interest

The authors declare no conflict of interest.

1

## Supporting information

As a service to our authors and readers, this journal provides supporting information supplied by the authors. Such materials are peer reviewed and may be re‐organized for online delivery, but are not copy‐edited or typeset. Technical support issues arising from supporting information (other than missing files) should be addressed to the authors.

Supporting Information

## Data Availability

The data that support the findings of this study are available in the supplementary material of this article.

## References

[1] M. L. Bochman , K. Paeschke , V. A. Zakian , Nat. Rev. Genet. 2012, 13, 770. 23032257 10.1038/nrg3296 PMC3725559

[2] G. W. Collie , G. N. Parkinson , Chem. Soc. Rev. 2011, 40, 5867–5892. 21789296 10.1039/c1cs15067g

[3] H. J. Lipps , D. Rhodes , Trends Cell Biol. 2009, 19, 414–422. 19589679 10.1016/j.tcb.2009.05.002

[4] D. Rhodes , H. J. Lipps , Nucleic Acids Res. 2015, 43, 8627–8637. 26350216 10.1093/nar/gkv862 PMC4605312

[5] J. Spiegel , S. Adhikari , S. Balasubramanian , Trends Chem. 2020, 2, 123–136. 32923997 10.1016/j.trechm.2019.07.002 PMC7472594

[6] D. Varshney , J. Spiegel , K. Zyner , D. Tannahill , S. Balasubramanian , Nat. Rev. Mol. Cell Biol. 2020, 21, 459–474. 32313204 10.1038/s41580-020-0236-x PMC7115845

[7] J. L. Huppert , S. Balasubramanian , Nucleic Acids Res. 2005, 33, 2908–2916. 15914667 10.1093/nar/gki609 PMC1140081

[8] J. L. Huppert , S. Balasubramanian , Nucleic Acids Res. 2006, 35, 406–413. 17169996 10.1093/nar/gkl1057 PMC1802602

[9] A. Bedrat , J.-L. Mergny , L. Lacroix , Nucleic Acids Res. 2016, 44, 1746–1759. 26792894 10.1093/nar/gkw006 PMC4770238

[10] G. Biffi , M. Di Antonio , D. Tannahill , S. Balasubramanian , Nat. Chem. 2013, 6, 75. 24345950 10.1038/nchem.1805 PMC4081541

[11] A. Henderson , Y. Wu , Y. C. Huang , E. A. Chavez , J. Platt , F. B. Johnson , J. R. M. Brosh , D. Sen , P. M. Lansdorp , Nucleic Acids Res. 2014, 42, 860–869. 24163102 10.1093/nar/gkt957 PMC3902944

[12] H.-Y. Liu , Q. Zhao , T.-P. Zhang , Y. Wu , Y.-X. Xiong , S.-K. Wang , Y.-L. Ge , J.-H. He , P. Lv , T.-M. Ou , J.-H. Tan , D. Li , L.-Q. Gu , J. Ren , Y. Zhao , Z.-S. Huang , Cell Chem. Biol. 2016, 23, 1261–1270. 27693060 10.1016/j.chembiol.2016.08.013

[13] V. S. Chambers , G. Marsico , J. M. Boutell , M. Di Antonio , G. P. Smith , S. Balasubramanian , Nat. Biotechnol. 2015, 33, 877. 26192317 10.1038/nbt.3295

[14] R. Hänsel-Hertsch , D. Beraldi , S. V. Lensing , G. Marsico , K. Zyner , A. Parry , M. Di Antonio , J. Pike , H. Kimura , M. Narita , D. Tannahill , S. Balasubramanian , Nat. Genet. 2016, 48, 1267. 27618450 10.1038/ng.3662

[15] A. Bugaut , S. Balasubramanian , Nucleic Acids Res. 2012, 40, 4727–4741. 22351747 10.1093/nar/gks068 PMC3367173

[16] C. K. Kwok , G. Marsico , A. B. Sahakyan , V. S. Chambers , S. Balasubramanian , Nat. Methods 2016, 13, 841–844. 27571552 10.1038/nmeth.3965

[17] S. Y. Yang , P. Lejault , S. Chevrier , R. Boidot , A. G. Robertson , J. M. Y. Wong , D. Monchaud , Nat. Commun. 2018, 9, 4730–4740. 30413703 10.1038/s41467-018-07224-8 PMC6226477

[18] M. J. Lista , R. P. Martins , O. Billant , M.-A. Contesse , S. Findakly , P. Pochard , C. Daskalogianni , C. Beauvineau , C. Guetta , C. Jamin , M.-P. Teulade-Fichou , R. Fåhraeus , C. Voisset , M. Blondel , Nat. Commun. 2017, 8, 16043. 28685753 10.1038/ncomms16043 PMC5504353

[19] F. Doria , E. Salvati , L. Pompili , V. Pirota , C. D′Angelo , F. Manoli , M. Nadai , S. N. Richter , A. Biroccio , I. Manet , M. Freccero , Chem. Eur. J. 2019, 25, 11085–11097. 31219221 10.1002/chem.201900766

[20] A. Piazza , J.-B. Boulé , J. Lopes , K. Mingo , E. Largy , M.-P. Teulade-Fichou , A. Nicolas , Nucleic Acids Res. 2010, 38, 4337–4348. 20223771 10.1093/nar/gkq136 PMC2910037

[21] L. T. Gray , E. Puig Lombardi , D. Verga , A. Nicolas , M.-P. Teulade-Fichou , A. Londoño-Vallejo , N. Maizels , Cell Chem. Biol. 2019, 26, 1681–1691. 31668518 10.1016/j.chembiol.2019.10.003

[22] M. Di Antonio , F. Doria , S. N. Richter , C. Bertipaglia , M. Mella , C. Sissi , M. Palumbo , M. Freccero , J. Am. Chem. Soc. 2009, 131, 13132–13141. 19694465 10.1021/ja904876q

[23] F. Doria , M. Nadai , M. Folini , M. Scalabrin , L. Germani , G. Sattin , M. Mella , M. Palumbo , N. Zaffaroni , D. Fabris , M. Freccero , S. N. Richter , Chem. Eur. J. 2013, 19, 78–81. 23212868 10.1002/chem.201203097 PMC3863998

[24] D. Verga , F. Hamon , F. Poyer , S. Bombard , M.-P. Teulade-Fichou , Angew. Chem. Int. Ed. 2014, 53, 994–998; 10.1002/anie.201307413 24338872

[25] M. Nadai , F. Doria , L. Germani , S. N. Richter , M. Freccero , Chem. Eur. J. 2015, 21, 2330–2334. 25512076 10.1002/chem.201405215

[26] K. Onizuka , M. E. Hazemi , N. Sato , G.-i. Tsuji , S. Ishikawa , M. Ozawa , K. Tanno , K. Yamada , F. Nagatsugi , Nucleic Acids Res. 2019, 47, 6578–6589. 31188442 10.1093/nar/gkz512 PMC6649768

[27] F. Nagatsugi , K. Onizuka , Chem. Lett. 2020, 49, 771–780.

[28] E. Cadoni , A. Manicardi , M. Fossépré , K. Heirwegh , M. Surin , A. Madder , Chem. Commun. 2021, 57, 1010–1013. 10.1039/d0cc06030e 33404017

[29] X. Zhang , J. Spiegel , S. Martínez Cuesta , S. Adhikari , S. Balasubramanian , Nat. Chem. 2021, 13, 626–633. 34183817 10.1038/s41557-021-00736-9 PMC8245323

[30] H. Su , J. Xu , Y. Chen , Q. Wang , Z. Lu , Y. Chen , K. Chen , S. Han , Z. Fang , P. Wang , B.-F. Yuan , X. Zhou , J. Am. Chem. Soc. 2021, 143, 1917–1923. 33471508 10.1021/jacs.0c10792

[31] S. Arumugam , J. Guo , N. E. Mbua , F. Friscourt , N. Lin , E. Nekongo , G.-J. Boons , V. V. Popik , Chem. Sci. 2014, 5, 1591–1598. 24765521 10.1039/C3SC51691A PMC3994131

[32] P. G. McCracken , J. L. Bolton , G. R. J. Thatcher , J. Org. Chem. 1997, 62, 1820–1825.

[33] M. P. McCrane , M. A. Hutchinson , O. Ad , S. E. Rokita , Chem. Res. Toxicol. 2014, 27, 1282–1293. 24896651 10.1021/tx500152d

[34] D. Verga , M. Nadai , F. Doria , C. Percivalle , M. Di Antonio , M. Palumbo , S. N. Richter , M. Freccero , J. Am. Chem. Soc. 2010, 132, 14625–14637. 20863115 10.1021/ja1063857

[35] D. Verga , S. N. Richter , M. Palumbo , R. Gandolfi , M. Freccero , Org. Biomol. Chem. 2007, 5, 233–235. 17205165 10.1039/b616292d

[36] W. F. Veldhuyzen , P. Pande , S. E. Rokita , J. Am. Chem. Soc. 2003, 125, 14005–14013. 14611237 10.1021/ja036943o

[37] S. Arumugam , V. V. Popik , J. Am. Chem. Soc. 2009, 131, 11892–11899. 19650661 10.1021/ja9031924

[38] C. Percivalle , A. La Rosa , D. Verga , F. Doria , M. Mella , M. Palumbo , M. Di Antonio , M. Freccero , J. Org. Chem. 2011, 76, 3096–3106. 21425810 10.1021/jo102531f

[39] F. Doria , C. Percivalle , M. Freccero , J. Org. Chem. 2012, 77, 3615–3619. 22397717 10.1021/jo300115f

[40] Đ. Škalamera , K. Mlinarić-Majerski , I. Martin-Kleiner , M. Kralj , P. Wan , N. Basarić , J. Org. Chem. 2014, 79, 4390–4397. 24758707 10.1021/jo500290y

[41] Đ. Škalamera , C. Bohne , S. Landgraf , N. Basarić , J. Org. Chem. 2015, 80, 10817–10828. 26461794 10.1021/acs.joc.5b01991

[42] F. Doria , S. N. Richter , M. Nadai , S. Colloredo-Mels , M. Mella , M. Palumbo , M. Freccero , J. Med. Chem. 2007, 50, 6570–6579. 18047263 10.1021/jm070828x

[43] S. N. Richter , S. Maggi , S. C. Mels , M. Palumbo , M. Freccero , J. Am. Chem. Soc. 2004, 126, 13973–13979. 15506758 10.1021/ja047655a

[44] W. J. Schreier , J. Kubon , N. Regner , K. Haiser , T. E. Schrader , W. Zinth , P. Clivio , P. Gilch , J. Am. Chem. Soc. 2009, 131, 5038–5039. 19309140 10.1021/ja900436t

[45] F. Doria , A. Lena , R. Bargiggia , M. Freccero , J. Org. Chem. 2016, 81, 3665–3673. 27035895 10.1021/acs.joc.6b00331

[46] H. Wang , S. E. Rokita , Angew. Chem. Int. Ed. 2010, 49, 5957–5960; 10.1002/anie.201001597 20632342

[47] R. A. Murphy , H. F. Kung , M. P. Kung , J. Billings , J. Med. Chem. 1990, 33, 171–178. 2136916 10.1021/jm00163a029

[48] K. Krohn , H. Rieger , K. Khanbabaee , Chem. Ber. 1989, 122, 2323–2330.

[49] L. Pretali , F. Doria , D. Verga , A. Profumo , M. Freccero , J. Org. Chem. 2009, 74, 1034–1041. 19123847 10.1021/jo802374r

[50] A. De Cian , L. Guittat , M. Kaiser , B. Saccà , S. Amrane , A. Bourdoncle , P. Alberti , M.-P. Teulade-Fichou , L. Lacroix , J.-L. Mergny , Methods 2007, 42, 183–195. 17472900 10.1016/j.ymeth.2006.10.004

[51] J. Dai , M. Carver , C. Punchihewa , R. A. Jones , D. Yang , Nucleic Acids Res. 2007, 35, 4927–4940. 17626043 10.1093/nar/gkm522 PMC1976458

[52] A. T. Phan , V. Kuryavyi , K. N. Luu , D. J. Patel , Nucleic Acids Res. 2007, 35, 6517–6525. 17895279 10.1093/nar/gkm706 PMC2095816

[53] Y. Wang , D. J. Patel , Structure 1993, 1, 263–282. 8081740 10.1016/0969-2126(93)90015-9

[54] A. T. Phan , V. Kuryavyi , H. Y. Gaw , D. J. Patel , Nat. Chem. Biol. 2005, 1, 167–173. 16408022 10.1038/nchembio723 PMC4690526

[55] A. Ambrus , D. Chen , J. Dai , R. A. Jones , D. Yang , Biochemistry 2005, 44, 2048–2058. 15697230 10.1021/bi048242p

[56] V. Kuryavyi , A. T. Phan , D. J. Patel , Nucleic Acids Res. 2010, 38, 6757–6773. 20566478 10.1093/nar/gkq558 PMC2965254

[57] S. Amrane , M. Adrian , B. Heddi , A. Serero , A. Nicolas , J.-L. Mergny , A. T. Phan , J. Am. Chem. Soc. 2012, 134, 5807–5816. 22376028 10.1021/ja208993r

[58] M. I. Onyshchenko , T. I. Gaynutdinov , E. A. Englund , D. H. Appella , R. D. Neumann , I. G. Panyutin , Nucleic Acids Res. 2009, 37, 7570–7580. 19820116 10.1093/nar/gkp840 PMC2794188

[59] K. W. Lim , P. Alberti , A. Guédin , L. Lacroix , J.-F. Riou , N. J. Royle , J.-L. Mergny , A. T. Phan , Nucleic Acids Res. 2009, 37, 6239–6248. 19692585 10.1093/nar/gkp630 PMC2764449

[60] J. S. Hudson , S. C. Brooks , D. E. Graves , Biochemistry 2009, 48, 4440–4447. 19348506 10.1021/bi900203z PMC3021945

[61] D. Renčiuk , I. Kejnovská , P. Školáková , K. Bednářová , J. Motlová , M. Vorlíčková , Nucleic Acids Res. 2014, 43, 1985–1985. 25539923 10.1093/nar/gku1274 PMC4330343

[62] G. Gottarelli , S. Lena , S. Masiero , S. Pieraccini , G. P. Spada , Chirality 2008, 20, 471–485. 17918751 10.1002/chir.20459

[63] V. Víglaský , L. Bauer , K. Tlučková , Biochemistry 2010, 49, 2110–2120. 20143878 10.1021/bi902099u

[64] P. Balagurumoorthy , S. K. Brahmachari , J. Biol. Chem. 1994, 269, 21858–21869. 8063830

[65] S. Redon , S. Bombard , M.-A. Elizondo-Riojas , J.-C. Chottard , Biochemistry 2001, 40, 8463–8470. 11456483 10.1021/bi001565a

[66] I. Ourliac-Garnier , M.-A. Elizondo-Riojas , S. Redon , N. P. Farrell , S. Bombard , Biochemistry 2005, 44, 10620–10634. 16060671 10.1021/bi050144w

[67] D. Gasparutto , C. Saint-Pierre , M. Jaquinod , A. Favier , J. Cadet , Nucleosides Nucleotides Nucleic Acids 2003, 22, 1583–1586. 14565471 10.1081/NCN-120023039

[68] I. Talhaoui , V. Shafirovich , Z. Liu , C. Saint-Pierre , Z. Akishev , B. T. Matkarimov , D. Gasparutto , N. E. Geacintov , M. Saparbaev , J. Biol. Chem. 2015, 290, 14610–14617. 25903131 10.1074/jbc.M115.647487 PMC4505527

