# Supplementary material for: Photoactivatable V‐Shaped Bifunctional Quinone Methide Precursors as a New Class of Selective G‐quadruplex Alkylating Agents
Source: Chemistry. 2022 May 12;28(35):e202200734. doi: 10.1002/chem.202200734 (PMC9322314; doi:10.1002/chem.202200734)

Chemistry–A European Journal

Supporting Information

Photoactivatable V-Shaped Bifunctional Quinone Methide Precursors as a New Class of Selective G-quadruplex Alkylating Agents

Alberto Lena, Alessandra Benassi, Michele Stasi, Christine Saint-Pierre, Mauro Freccero, Didier Gasparutto, Sophie Bombard, Filippo Doria,* and Daniela Verga*

Author Contributions

A.L. Conceptualization:Equal; Formal analysis:Equal; Project administration:Equal; Writing – review & editing:Equal
A.B. Formal analysis:Equal; Funding acquisition:Equal; Investigation:Equal; Writing – review & editing:Equal
M.S. Investigation:Supporting; Writing – review & editing:Supporting
C.S.-P. Formal analysis:Supporting; Writing – review & editing:Supporting
M.F. Conceptualization:Equal; Writing – review & editing:Supporting
D.G. Conceptualization:Supporting; Funding acquisition:Equal; Writing – review & editing:Supporting
S.B. Supervision:Supporting; Writing – review & editing:Supporting
F.D. Data curation:Supporting; Supervision:Equal; Writing – review & editing:Equal
D.V. Conceptualization:Lead; Data curation:Lead; Funding acquisition:Equal; Project administration:Lead; Supervision:Equal; Writing – original draft:Lead; Writing – review & editing:Lead

Table of Contents

	Page
**Experimental section**	**S2**
**Synthetic Procedures**	**S3**
**Photochemical studies**	**S9**
**Biophysical assays**	**S10**
**Biochemical assays**	**S11**
**MALDI-ToF Mass Spectrometry Analysis**	**S13**
**Figure S1.** Molar absorbivity of the ligand in water and in buffer.	**S14**
**Tables S1.** Photophysical properties of synthesized compounds	**S14**
**Figure S2-S4.** LFP spectra of compounds **1-4.**	**S15-S17**
**Figure S5.** CD spectra of the ligands **1-6.**	**S18**
**Table S2.** Isoelliptic points identified by CD titration, performed in the presence of 22AG, with ligand **1-6.**	**S19**
**Figure S6-S11.** Titration data of compounds **1-6.**	**S20-S22**
**Table S3.** Binding constants compounds **1-6**.	**S23**
**Figure S12-S17.** Denaturing gel electrophoresis of alkylation products.	**S24-S27**
**Figure S18-S19.** Sequencing gels.	**S28-S29**
**Figure S20-S21.** Chromatographic profiles of alkylation reactions.	**S30**
**Figure S22.** MALDI-ToF mass spectrum analysis alkylation product compound **4**.	**S31**
**Figure S23-S24.** MALDI-ToF mass spectrum analysis of digestion by crotalus venom phosphodiesterase I	**S32-S33**
**HPLC Purity Data**	**S34-S36**
**^{1}H-NMR and ^{13}C-NMR Spectra**	**S37-S51**
**ESI-MS Data**	**S52-S55**

Experimental section

General Information. Solvents and chemicals were purchased from TCI or Merck and were used as supplied without further purification. TLC analysis was carried out on silica gel (Merck 60F-254) with visualisation at λ=254 and 366 nm. Flash column chromatography purification have been performed with Isolera ONE Flash Chromatography System (MPLC, Biotage), combined with a tunable wavelength UV/vis detector (SNAP KP-SIL 10, 25 or 50 g columns, Biotage). HPLC analysis and HPLC purification were performed using two different systems: Agilent system SERIES 1260 combining a preparative PUMP, a PDA and a fraction collector (for preparative HPLC), and an Agilent system SERIES 1260 (for analytical HPLC). The analytical column was a XSelect CSH C18 2.5 μm (150×4.6 mm^2) (Waters). The preparative column was a XSelect CSH Prep C18 5 μm (100×30 mm^2) (Waters). Flows were 1.0 mL/min for analytical and 30 mL/min for preparative runs. For all analysis and purifications, we have employed, as solvents, 0.1% trifluoroacetic acid in water and CH_3CN. For the analytical analysis the following method A was used: isocratic gradient at 5% CH_3CN for 2 minutes, gradually increased to 100% over 10 minutes, then isocratic flow at 5% for 2 minutes. Preparative HPLC was performed by using method B: isocratic gradient over 2 minutes at 5% CH_3CN, gradually increased to 50% over 15 minutes and, subsequently, to 100% in 7 minutes, then isocratic flow at 5% for 2 minutes. ^{1}H- and ^{13}C-NMR spectra were recorded on a Bruker ADVANCE 300MHz spectrometer. UV-visible spectra were measured in an Agilent Cary-300 spectrophotometer equipped with a Peltier temperature controller. The absorbance was recorded for 200-900 nm wavelength interval at a scan rate of 200 nm/min and a slit width of 1.5 nm, with a 3 ml quartz cell with 1 cm path length.

Doubly-labelled oligonucleotides for FRET melting experiments were synthesized and purified by Eurogentec, desalted on a Sephadex G25 column and stored at -20 °C as a 250 μM aqueous solution. 22AG d[AGGGTTAGGGTTAGGGTTAGGG], Pu24T d[TTGAGGGTGGGTAGGGTGGGTAA], and ihTel d[CCCTAACCCTAACCCTAACCCT] for gel experiments were synthesized and PAGE purified by Eurogentec and stored at -20 °C as a 1 mM aqueous solution. The oligonucleotide was 5'-end-labeled using bacterial T4 polynucleotide kinase (purchased Lucigen) and [γ-^{32}P]ATP (purchased PerkinElmer).

Synthetic Procedures

We synthesised the starting intermediates **p-7**, **m-7** and **9** according to published procedures.[1,2]

Synthesis of V-shaped 4- and 5- arylethynyl benzo- Mannich bases (1 and 3)

The corresponding aryl iodide **p,m-7** (0.300 g, 1.08 mmol), $PdCl_2(PPh_3)_2$ (76 mg, 10 mol %), and copper (I) iodide (41 mg, 20 mol %) were dissolved in dry DMF (15 mL). After 5 minutes of bubbling with argon, a solution of 1,3-diethynybenzene (72 µL, 0.54 mmol) in neat triethylamine (1.5 mL, 10.8 mmol) was added under inert atmosphere, and the mixture was stirred at room temperature for 10 hours. After this period of time, the solvent was removed under reduced pressure. The crude was dissolved in DCM (50 mL) and washed with 1% $NaHCO_3$ (3x30 mL). The organic phase was dried over Na_2SO_4 and the solvent removed under reduced pressure. The obtained product was purified by reverse phase HPLC (H_2O 0.1% TFA/CH_3CN, method B). The aqueous phase was neutralized with $NaHCO_3$, extracted in DCM (3x10 mL), and the combined organic phases were dried over Na_2SO_4, and the solvent was removed under reduced pressure. **TLC** (AcOEt/MeOH 8:2) R_f 0.20.

Compound **1**. White solid (Yield = 38%); ^{1}H-NMR (300 MHz, $CDCl_3$, 25 °C, TMS) δ (ppm) 10.28 (bs, 2H), 7.65 (s, 1H), 7.45–7.28 (m, 5H), 7.19 (d, J = 1.7 Hz, 2H), 6.84 (d, J = 8.4 Hz, 2H), 3.66 (s, 4H), 2.36 ppm (s, 12H); ^{13}C-NMR (75 MHz, $CDCl_3$, 25°C, TMS) δ (ppm) 158.7, 134.0, 132.4, 131.7, 130.5, 128.3, 123.9, 121.9, 116.3, 113.1, 90.1, 86.8, 62.3, 44.3. Anal. calcd for $C_{28}H_{28}N_2O_2$ (%): C, 79.22; H, 6.65; N, 6.60. Found (%): C, 79.19; H, 6.66; N, 6.64.

Compound **3**. White solid (Yield = 25%); ^{1}H-NMR (300 MHz, $CDCl_3$, 25 °C, TMS) δ (ppm) 8.64 (bs, 2H), 7.71 (s, 1H), 7.49 (d, J = 7.8 Hz, 2H), 7.34 (t, J = 7.3 Hz, 1H), 7.02–6.96 (m, 6H), 3.67 (s, 4H), 2.35 ppm (s, 12H); ^{13}C-NMR (75 MHz, $CDCl_3$, 25°C, TMS) δ (ppm) 159.7, 136.3, 132.9, 130.1, 130.0, 125.4, 124.9, 124.5, 124.3, 120.7, 91.7, 89.9, 64.4, 46.2. Anal. calcd for $C_{28}H_{28}N_2O_2$ (%): C, 79.22; H, 6.65; N, 6.60. Found (%): C, 79.21; H, 6.64; N, 6.65.

Synthesis of V-shaped 4- and 5- arylethynyl benzo- ammonium salts (2 and 4)

Iodomethane (11 µL, 0.17 mmol) was added to a stirred solution of the corresponding Mannich base (10 mg, 0.029 mmol) in CH_3CN (10 mL) and the reaction mixture was stirred at room temperature for 48 hours. The solvent and excess iodomethane were removed under reduced pressure. The product was purified by reverse phase HPLC (H_2O 0.1% TFA/CH_3CN, method B) and the anion exchanged with 1% HCl (1 mL).

Compound **2**. White solid (yield = 97%); ^{1}H-NMR (300 MHz, CD_3OD, 25 °C, TMS) δ (ppm) 7.67 (s, 2H), 7.65 (s, 1H), 7.60-7.56 (m, 2H), 7.5-7.49 (m, 3H), 7.04-6.97 (m, 2H), 4.6 (s, 4H), 3.2 (s, 18H). ^{13}C-NMR (75 MHz, CD_3OD, 25 °C, TMS) δ (ppm) 159.5, 139.4, 137.2, 135.3, 132.3, 130.2, 125.4, 117.9, 116.6, 116.0, 90.4, 88.7, 65.2, 53.9. Anal. calcd for $C_{30}H_{34}Cl_2N_2O_2$ (%): C, 68.57; H, 6.52; N, 5.33. Found (%): C, 68.60; H, 6.51; N, 5.31.

Compound **4**. White solid (yield = 99%); ^{1}H-NMR (300 MHz, CD_3OD, 25 °C, TMS) δ (ppm) 7.70 (s, 1H), 7.59 (s, 1H), 7.56-7.49 (m, 4H), 7.18-7.16 (m, 4H), 4.60 (s, 4H), 3.19 (s, 18H). ^{13}C-NMR (75 MHz, CD_3OD, 25 °C, TMS) δ (ppm) 159.0, 136.3, 135.7, 133.2, 130.5, 128.6, 124.9, 124.5, 120.1, 116.8, 90.8, 90.3, 65.4, 54.0, 53.9. Anal. calcd for $C_{30}H_{34}Cl_2N_2O_2$ (%): C, 68.57; H, 6.52; N, 5.33. Found (%): C, 68.59; H, 6.53; N, 5.34.

Synthesis of 7-bromo-3-hydroxy-N,N-dimethyl-2-naphthamide (10)

7-Bromo-3-hydroxynaphthalene-2-carboxylic acid **9** (2 g, 0.0075 mol, 1 eq.) was dissolved into 20 ml of thionyl chloride and the reaction mixture was stirred at 80°C for four hours. After, thionyl chloride was removed under vacuum and the obtained red solid was dissolved into 8 ml of dichlorometane. In the meantime, in a one-necked flask, 490 mg of sodium hydroxide (0.009 mol, 1.2 eq.) were dissolved in 30 ml (0.9 mol, 100 eq.) of dimethylamine solution (11% in ethanol) and cooled to 0 °C with an ice bath. The acyl chloride was transferred into a dropping funnel and added dropwise to the dimethylamine solution. The reaction mixture was stirred for one hour at 0°C, then 100 ml of HCl 10% were added to induce product precipitation. The obtained suspension was filtered in a Buchner funnel and 2.01 g of a white solid were collected and used for the subsequent step without purification. White solid (Yield = 93%). ^{1}H-NMR (300 MHz, DMSO-d_6, 25 °C, TMS) δ (ppm) 10.35 (s, 1H), 8.09 (s, 1H), 7.71 (m, 2H), 7.52 (d, *J*= 8.86 Hz, 1H), 7.23 (s, 1H), 3.00 (s, 3H), 2.80 (s, 3H) ^{13}C-NMR (75 MHz, DMSO-d_6, 25°C, TMS) δ (ppm) 167.8, 152.0, 132.8, 129.5, 129.0, 128.5, 128.1, 126.4, 115.9, 109.2, 37.6, 34.1. Anal. calcd for $C_{13}H_{12}BrNO_2$ (%): C, 53.08; H, 4.11; N, 4.76. Found (%): C, 53.10; H, 4.10; N, 4.75.

Synthesis of 6-bromo-3-((dimethylamino)methyl)naphthalene-2-ol (11)

7-bromo-3-hydroxy-N,N-dimethyl-2-naphthamide **10** (8.88 g, 30.2 mmol) was suspended in anhydrous THF (250 mL) and the mixture was cooled at 0°C. Lithium aluminium hydride (2.5 g, 65.9 mmol) was added in one portion under stirring and the reaction mixture was heated at 70 °C for 2 hours. Hence, the mixture was cooled in an ice bath and quenched with a saturated aqueous $NaHCO_3$ solution (100 mL). The organic solvent was removed under reduced pressure and the aqueous phase was extracted with DCM (3x50 mL). The combined organic phases were washed with brine and dried over Na_2SO_4. The solvent was removed under reduced pressure, and the residue was purified by recrystallization in ethanol. **TLC** ($CHCl_3$/MeOH 95:5) R_f 0.56. White solid (yield = 96%). ^{1}H-NMR (300 MHz, DMSO-d_6, 25 °C, TMS) δ (ppm) 8.11 (s, 1H), 7.77-7.72 (m, 2H), 7.57 (d, *J*= 8.79 Hz), 7.24 (s, 1H), 3.85 (s, 2H), 2.39 (s, 6H). =^{13}C-NMR (75 MHz, DMSO-d_6, 25 °C, TMS): δ (ppm)156.1, 132.4, 129.0, 128.8, 128.7, 128.6, 128.0, 127.3, 126.9, 115.3, 111.6, 109.0, 60.4, 44.3. Anal. calcd for $C_{13}H_{14}BrNO$ (%): C, 55.73; H, 5.04; N, 5.00. Found (%): C, 55.76; H, 5.05; N, 5.00.

Synthesis of 3-((dimethylamino)methyl)-6-((trimethylsilyl)ethynyl)naphthalen-2-ol (12)

6-bromo-3-((dimethylamino)methyl)naphthalene-2-ol **11** (500 mg, 1.78 mmol), $PdCl_2(PPh_3)_2$ (50 mg, 4% mol), and copper (I) iodide (13.6 mg, 4% mol) were added into a three necked round-bottomed flask equipped with a condenser and a magnetic stirrer and kept under Ar atmosphere. Trimethylsilylacetylene (378 μL, 2.68 mmol) was dissolved in triethylamine (20 mL), degassed with Ar for 5 minutes, and added in the reaction flask. The reaction mixture was stirred at 80 °C for 1 hour. The mixture was filtered through a thin pad of celite, washed with chloroform (3x20 mL), and the solvent was removed under reduced pressure. The crude was purified by flash chromatography (cyclohexane/AcOEt gradient). **TLC** ($CHCl_3$/MeOH 9:1) R_f 0.6. White solid (yield = 86%). ^{1}H-NMR (300 MHz, $CDCl_3$, 25 °C, TMS) δ (ppm)= 7.86 (s, 1H), 7.60 (d, J = 8.5 Hz, 1H), 7.46–7.39 (m, 2H), 7.16 (s, 1H), 3.83 (s, 2H), 2.40 (s, 6H), 0.30 (s, 9H); ^{13}C-NMR (75 MHz, $CDCl_3$, 25 °C, TMS) δ (ppm)= 157.0, 134.1, 131.4, 128.7, 127.3, 127.2, 126.0, 125.2, 117.3, 110.3, 105.9, 93.2, 62.7, 44.3, 0.0. Anal. calcd for $C_{18}H_{23}NOSi$ (%): C, 72.68; H, 7.79; N, 4.71. Found (%): C, 72.69; H, 4.69; N, 4.70.

Synthesis of 3-((Dimethylamino)methyl)-6-ethynylnaphthalen-2-ol (13)

3-((dimethylamino)methyl)-6-((trimethylsilyl)ethynyl)naphthalen-2-ol **12** (400 mg, 1.34 mmol) was dissolved in methanol (25 mL) and potassium carbonate (204 mg, 1.608 mmol) was added in one portion. The suspension was stirred at room temperature for 16 hours. The solvent was removed under reduced pressure and the crude was treated with 1% HCl (10 mL) and neutralized with $NaHCO_3$. The product was extracted in DCM (3x20 mL), the combined organic phases were dried over Na_2SO_4 and the solvent was removed under reduced pressure. The crude was purified by flash chromatography (cyclohexane/AcOEt gradient). **TLC** ($CHCl_3$/MeOH 9:1) R_f 0.69. White solid (yield = 59%); ^{1}H-NMR (300 MHz, $CDCl_3$, 25 °C, TMS): δ (ppm)= 9.85 (bs, 1H), 7.88 (s, 1H), 7.63 (d, J = 8.5 Hz, 1H), 7.45 (d, J = 7.8, 1H), 7.44 (s, 1H), 7.17 (s, 1H), 3.82 (s, 2H), 3.11 (s, 1H), 2.38 ppm (s, 6H); ^{13}C-NMR (75 MHz, $CDCl_3$, 25 °C, TMS): δ (ppm) = 156.9, 133.9, 131.3, 128.3, 128.0, 126.9, 125.8, 125.1, 115.8, 109.9, 84.0, 76.0, 62.5, 44.0. Anal. calcd for $C_{15}H_{15}NO$ (%): C, 79.97; H, 6.71; N, 6.22. Found (%): C, 79.94; H, 6.72; N, 6.21.

Synthesis of 6,6'-(1,3-phenylenebis(ethyne-2,1-diyl))bis(3-((dimethylamino)methyl)naphthalen-2-ol) (5). 1,3-diiodobenzene **14** (73 mg, 0.22 mmol), $PdCl_2(PPh_3)_2$ (12.4 mg, 4% mol), and copper (I) iodide (3.4 mg, 4% mol) were added into a three necked round-bottomed flask equipped with a condenser and a magnetic stirrer and kept under Ar atmosphere. 3-((dimethylamino)methyl)-6-ethynylnaphthalen-2-ol **13** (100 mg, 0.44 mmol) was dissolved in triethylamine (10 mL), degassed with Ar for 5 minutes and added in the reaction flask. The reaction mixture was stirred at 80 °C for 2 hours. The residue was dissolved in DCM (25 mL) and washed with 1% $NaHCO_3$ (3x20 mL). The organic phase was dried over Na_2SO_4 and the solvent was removed under reduced pressure. The crude was purified by reverse phase HPLC

(H_2O 0.1% TFA/CH_3CN gradient) with method B. The aqueous phase was neutralized with $NaHCO_3$, extracted in DCM (3x10 mL), and the combined organic phases were dried over Na_2SO_4. At last, the solvent was removed under reduced pressure. **TLC** ($CHCl_3$/MeOH 95:5) R_f 0.44. White solid (yield = 81%); ^{1}H-NMR (300 MHz, DMSO-d$_6$, 25 °C, TMS) δ (ppm)8.06 (s, 2H), 7.77–7.74 (m, 5H), 7.62 (d, J = 7.2 Hz, 2H), 7.53-7.49 (m, 3H), 7.19 (s, 2H), 3.89 (s, 4H), 2.39 ppm (s, 12H); ^{13}C-NMR (75 MHz, DMSO-d$_6$, 25 °C, TMS) δ (ppm)= 156.6, 133.8, 133.7, 131.2, 131.1, 129.4, 128.7, 128.2, 127.0, 126.3, 125.9, 123.2, 116.0, 109.2, 91.0, 87.8, 59.6, 44.0. Anal. calcd for $C_{36}H_{32}N_2O_2$ (%): C, 82.41; H, 6.15; N, 5.34. Found (%): C, 82.39; H, 6.14; N, 5.35.

Synthesis of 1,1'-((1,3-phenylenebis(ethyne-2,1-diyl))bis(3-hydroxynaphthalene-7,2-diyl))bis(N,N,N-trimethylmethanaminium) chloride (6). Iodomethane (268 µL, 4.3 mmol) and sodium acetate (14.8 mg, 0.18 mmol) were added to a stirred solution of the Mannich base **5** (94 mg, 0.18 mmol) in CH_3CN (10 mL) and the reaction mixture was stirred at room temperature for 24 hours. After this time, further iodomethane (67 µL, 1.08 mmol) was added and the suspension was stirred at room temperature for additional 16 hours. Hence, the solvent and excess iodomethane were removed under reduced pressure. The product was purified by reverse phase HPLC (H_2O 0.1% TFA/CH_3CN gradient) with method B and the anion was exchanged with 1% HCl (1 mL). White solid (yield = 68%); ^{1}H-NMR (300 MHz, CD_3OD, 25 °C, TMS) δ (ppm) 8.06 (s, 4H), 7.73–7.69 (m, 3H), 7.59–7.52 (m, 4H), 7.41 (dd, J =8.3, 7.0 Hz, 1H), 7.33 (s, 2H), 4.72 (s, 4H), 3.21 (s, 18H); ^{13}C-NMR (75 MHz, CD_3OD, 25 °C, TMS): δ (ppm)= 156.7, 137.4, 137.0, 135.5, 133.3, 132.7, 131.6, 130.3, 129.0, 127.7, 125.4, 119.7, 119.6, 111.6, 91.5, 89.7, 65.6, 53.9. Anal. calcd for $C_{38}H_{38}Cl_2N_2O_2$ (%): C, 72.95; H, 6.12; N, 4.48. Found (%): C, 72.92; H, 6.11; N, 4.49.

Synthesis of 1-(2-hydroxy-4-((3-((3-hydroxy-4-(hydroxymethyl) phenyl)ethynyl)phenyl) ethynyl) phenyl)-N,N,N-trimethylmethanaminium (16) and 4,4'-(1,3-phenylenebis(ethyne-2,1-diyl))bis(2 (hydroxymethyl)phenol) (17). Compound **2** (0.02 mmol) has been dissolved into 200 ml of distilled water to achieve concentration of 0.1 mM. The solution was transferred into a quartz tube and irradiated with a Rayonet at 310 nm (32W, 2 lamps) for 30 minutes. Then solvent was removed under vacuum and the crude was purified by reverse phase HPLC (H_2O 0.1% TFA/CH_3CN gradient) with method B.

Compound **16**. Yellow oil (Yield = 62%); ^{1}H-NMR (300 MHz, DMSO-d$_6$, 25 °C, TMS) δ (ppm) 10.96 (s, 1H), 9.91 (s, 1H), 7.66-7.45 (m, 7H), 7.27-7.26 (m, 1H), 7.05 (d, J= 8.51 Hz, 1H), 6.82 (d, J= 8.3 Hz, 1H), 4.48 (s, 2H), 4.4.46 (s, 2H), 3.07 (s, 9H). ^{13}C-NMR (75 MHz, DMSO-d$_6$, 25 °C, TMS) δ (ppm) 158.1, 158.0, 155.0, 138.0, 135.3, 131.0, 130.9, 130.6, 129.4, 129.3, 123.7, 123.1, 116.8, 115.5, 114.9, 112.7, 111.8, 91.3, 89.7, 87.2, 86.1, 62.6, 57.7, 52.2, 47.7. Anal. calcd for $C_{27}H_{26}ClNO_3$ (%): C, 72.39; H, 5.85; N, 3.13. Found (%): C, 72.43; H, 5.85; N, 3.12.

Compound **17**. Yellow oil (Yield = 8%); ^{1}H-NMR (300 MHz, DMSO-d_6, 25 °C, TMS) δ (ppm) 9.95 (s, 2H), 7.60 (s, 1H), 7.50-7.40 (m, 5H), 7.28 (d, *J*= 8.29 Hz, 2H), 6.81 (d, *J*= 8.27 Hz, 2H), 4.48 (s, 4H). ^{13}C-NMR (75 MHz, DMSO-d_6, 25 °C, TMS) δ (ppm) 163.0, 157.9, 155.0, 152.1, 133.2, 130.9, 130.7, 130.6, 129.4, 129.2, 123.6, 114.9, 111.9, 99.7, 90.6, 86.3, 60.1, 57.7, 56.2. Anal. calcd for $C_{24}H_{18}O_4$ (%): C, 77.82; H, 4.90. Found (%): C, 77.80; H, 4.89.

Synthesis of 1-(2-hydroxy-4-((3-((3-hydroxy-4-(hydroxymethyl)phenyl) ethynyl) phenyl)ethynyl)phenyl)-N,N,N-trimethylmethanaminium (19) and 5,5'-(1,3-phenylenebis(ethyne-2,1-diyl))bis(2-(hydroxymethyl)phenol) (20). Compound **4** (0.2 mmol) was dissolved into 250 ml of distilled water to achieve concentration of 0.1 mM. The solution was transferred into a quartz tube and irradiated with a Rayonet at 310 nm (32W, 2 lamps) for one hour. Then solvent was removed under vacuum and the crude was purified by reverse phase HPLC (H_2O 0.1% TFA/CH_3CN gradient) with method B.

Compound **19**. Yellow oil (Yield =43 %); ^{1}H-NMR (300 MHz, DMSO-d_6, 25 °C, TMS) δ (ppm) 10.79 (s, 1H), 7.73 (s, 1H), 7.62-7.43 (m, 4H), 7.36 (d, *J*= 8.29 Hz, 1H), 7.15-7.13 (m, 2H), 7.03 (d, *J*= 8.70 Hz, 1H), 6.94 (s, 1H), 4.50 (s, 2H), 4.47 (s, 2H), 3.05 (s, 9H). ^{13}C-NMR (75 MHz, DMSO-d_6, 25°C, TMS) δ (ppm) 157.3, 154.0, 135.1, 133.9, 131.8, 131.5, 130.6, 129.4, 127.4, 125.3, 123.2, 122.5, 122.5, 122.3, 120.4, 118.4, 116.8, 115.9, 90.6, 89.2, 89.2, 87.1, 62.7, 57.9, 52.1. Anal. calcd for $C_{27}H_{26}ClNO_3$ (%): C, 72.39; H, 5.85; N, 3.13. Found (%): C, 72.37; H, 5.87; N, 3.13.

Compound **20**. Yellow oil (Yield =5 %) ^{1}H-NMR (300 MHz, DMSO-d_6, 25 °C, TMS) δ (ppm) 9.73 (s, 2H), 7.69 (s, 1H), 7.57 (d, *J*= 7.2 Hz, 2H), 7.50-7.44 (m, 1H), 7.35 (d, *J*= 7.8 Hz, 2H), 7.03 (d, *J*= 7.77 Hz, 2H), 6.93 (s, 2H), 4.50 (s, 4H). ^{13}C-NMR (75 MHz, DMSO-d_6, 25°C, TMS) δ (ppm) 161.1, 153.9, 133.7, 131.3, 130.5, 129.3, 127.4, 123.1, 122.3, 120.5, 116.8, 90.5, 87.3, 57.9. Anal. calcd for $C_{24}H_{18}O_4$ (%): C, 77.82; H, 4.90. Found (%): C, 77.79; H, 4.91.

Synthesis of 1-(5-((3-((2-ethoxychroman-6-yl)ethynyl)phenyl)ethynyl)-2-hydroxyphenyl)-*N,N,N*-trimethylmethanaminium chloride (21). Compound **2** (0.02 mmol) has been dissolved into 200 ml of 1:1 CH_3CN:phosphate buffer (pH 7.4) to achieve concentration of 0.1 mM. The solution was transferred into a quartz tube and irradiated with a Rayonet at 310 nm (32W, 2 lamps) for 15 minutes. Then solvent was removed under vacuum and the crude was purified by reverse phase HPLC (H_2O 0.1% TFA/CH_3CN gradient) with method B.

Compound **21**. Yellow oil (Yield 60%). ^{1}H-NMR (300 MHz, DMSO-d_6, 25 °C, TMS) δ (ppm); 10.96 (s, 1H), 7.64 (d, *J* = 5.58 Hz, 2H), 7.59-7.46 (m, 4H), 7.31 (s, 2H), 7.05 (d, J = 8.47 Hz, 1H), 6.84 (d, *J* = 8.93 Hz, 1H), 5.35 (s, 1H), 4.45 (s, 2H), 3.82-3.61 (m, 2H), 3.07 (s, 9H), 2.88-2.62 (m, 2H), 2.02-1.85 (m, 2H), 1.13 (t, J = 7.03 Hz, 3H). ^{13}C-NMR (75 MHz, DMSO-d_6, 25°C, TMS) δ (ppm) 158.2, 152.9, 138.0, 133.4, 132.6, 131.0, 130.7, 129.4, 123.2, 123.1, 117.2, 116.8, 115.5, 113.7, 112.7, 96.8, 89.8, 86.7, 63.3, 52.2, 25.7, 19.6, 15.1.

References

[1] F. Doria, A. Lena, R. Bargiggia and M. Freccero, *J. Org. Chem.* **2016**, *81*, 3665-3673.

[2] C.-J. Qiao, X.-K. Wang, F. Xie, W. Zhong and S. Li, *Molecules* **2015**, *20*, DOI: 10.3390/molecules201219846.

Photochemical studies

Quantum yield measurement

Reaction quantum yield (Φ) were obtained by irradiating a 3 mL solution (1:1 CH_3CN:PBS 50mM pH=7.4) of the desired QMP (concentration 10^{-4} to 10^{-5}) in a 1 cm optical path cuvette and monitoring the consumption of the QMP by reverse-phase HPLC. The lamp source was a focalized 150 W high-pressure mercury arc equipped with a transmittance filter (transmission 313nm) and a single-photon detector (photon flux q_p=4.3E-9 s^{-1}). Calibration of the apparatus was performed using the photochemical decomposition of potassium ferrioxalate as actinometer. Quantum yield values have been determined by using the following equation:

$$\Phi\ (313\ nm) = (mol\ of\ QMP\ reacted) / (mole\ of\ absorbed\ photons)$$

LFP Experiments

Nanosecond Laser Flash Photolysis profiles were measured by means of a photolysis apparatus. The spectra were reconstructed point from time profiles taken each 5 nm. The minimum response time of the detection system was of about 10 ns. The laser beam (Nd/YAG operated at λ = 266 nm) was focused on a 3 mm wide circular area of the cell and the first 5 mm in depth were analyzed at a right angle geometry. The incident pulse energies used were 4-5 mJ per pulse. The sample absorbance at 266 nm was typically 0.8-1.0 over 1 cm. When required, oxygen was removed from the solution by vigorously bubbling with argon. The temperature was kept constant at 298±2 K. Acquisition and processing of the absorption signals were performed by LP900 7.0.2 (Build 0) Software. Non-linear fitting procedures by the least square method was used to judge the goodness of the fit.

Biophysical assays

FRET-melting assay

FRET-melting assays were performed with 0.2 μM FAM (6-carboxyfluorescein) 5′-end- and Tamra (6-carboxy-tetramethylrhodamine) 3′-end-labelled oligonucleotides in 10 mM lithium cacodylate buffer (pH 7.2), 99 mM LiCl and 1 mM KCl (90 mM LiCl and 10 mM KCl for F21T, F(21CTA)T and FdxT oligonucleotides), heat denatured for 5 min at 95 °C, and folded in G4 structure by cooling the solutions at 4 °C overnight or left to slowly cool down at room temperature for hairpin duplex. Before the analysis, the proper amount of competitor (ds26) was added, followed by the tested compound (concentration from 1 μM to 20 μM) or water as a reference. Fluorescence melting curves were measured with a 7900HT Fast Real-Time quantitative PCR machine (Applied Biosystems, Thermo Fischer Scientific), using 96-well plates and a total reaction volume of 25 μL. After a first equilibration step at 25 °C for 5 minutes, a stepwise increase of 0.5 °C every 30 seconds for 140 cycles was performed to reach 95 °C. A measurement was completed after each cycle by reading the FAM channel. Oligonucleotide melting was monitored by observing 6-carboxyfluorescein (6-FAM) emission, which was normalized between 0 and 1. The melting temperature ($T_{1/2}$) was defined as the temperature at the inflection point of the sigmoid dose-response best fitting curve. Labelled sequences for FRET-melting assays: F21T 5'-Fam-GGGTTAGGGTTAGGGTTAGGG-Tamra-3', F(21CTA)T 5'-Fam-GGGCTAGGGCTAGGGCTAG GG-Tamra-3', F(Pu24T)T 5'-Fam-TGAGGGTGGTGAGGGTGGGGAAGG-Tamra-3', F(c-Myc)T 5'-Fam-TTGAGGGTGGGTAGGGTGGGTAA-Tamra-3', F(c-Kit2)T 5'-Fam-GGGCGGGCGCGAGGG AGGGG-Tamra-3', F(Bcl2)T 5'-Fam-AGGGGCGGGCGCGGGAGGAAGGGGGCGGGA-Tamra-3', F(CEB25wt)T 5'-Fam-AAGGGTGGGTGTAAGTGTGGGTGGGT-Tamra-3', FdxT 5'-Fam-TATAGCTAT-HEG-ATAGCTATA-Tamra and competitor ds DNA sequence ds26 5'-CAATCGGATCGAATTCGATCCGATTG-3'.

Circular dichroism

CD titrations were performed using a JASCO J-710 spectropolarimeter equipped with a Peltier temperature controller (Jasco PTC-348WI) and 1 mL black-walled rectangular quartz cells with 1 cm path length. The reaction volume was 1 mL. Prior measurements, 3 μM oligonucleotide in 10 mM lithium cacodylate buffer (pH 7.2) and 100 mM KCl or NaCl were heat denatured for 5 min at 95 °C, and folded in G4 structure by cooling the solution at 0 °C for 30 minutes. The G4 was titrated with a 2 mM solution of the tested compound. The titration was stopped after adding 1–10, 12, or 14 equiv of the tested compound. Scans were recorded from 220 to 450 nm with 50 nm min^{-1} scan speed, 1.0 nm band width, 0.5 s integration time, 0.5 nm data pitch, and were corrected by a baseline obtained from the buffer in the same conditions. The signal, as an average of 4 accumulations, was further smoothened with a Savitzky-Golay method (2 order, 20 points window).

Biochemical assays

Oligonucleotide 5'-end labelling and purification

^{32}P-5'-end labelling was achieved by incubating at 37 °C for 30 minutes a solution prepared by mixing 1 µL of 20 µM PAGE-purified 22AG d[AGGGTTAGGGTTAGGGTTAGGG] or Pu24T d[TGAGGGTGGTGAGGGTGGGGAAGG], 3 µL of 3.33 µM (.ca 10 mCi/mL) [γ-^{32}P]-ATP (PerkinElmer), 2 µL of T4 Polinucleotide Kinase (T4 PNK, 10^4 Richardson units/mL, Tris-HCl 10 mM, KCl 50 mM, 1,4-Dithiothreitol (DTT) 1 mM, EDTA 0.1 mM, 50% v/v Glycerol, ATP 0.1 µM, pH 7.4 at 25°C), 1 µL of 10X T4 PNK reaction buffer and 3 µL of water. The oligonucleotides were subsequently purified by gel filtration on Sephadex® G-25 centrifugal filters or PAGE. Radioactivity incorporation was eventually measured on 1 µL samples by β-emission counting on a Tri-Carb® 2910TR Liquid Scintillator Analyser. For a simple irradiation-PAGE analysis an amount of solution corresponding to about $5 \cdot 10^4$ cpm was used. For subsequent band isolation and secondary gels or sequencing assays the freshly prepared labelled oligonucleotide was used in its entirety after concentration in a SpeedVacTM system (≈ 10^7 cpm).

Irradiation of the DNA-Ligand complexes

The irradiations were performed with a MAX-303 Xenon light source (Asahi) provided with a filter centered at 365 nm, a quartz light guide and a collimator lens. The apparatus was setup in a top-down fashion, exposing an ice-cooled uncapped 1.5 mL Eppendorf tube to the UV beam at a distance of 120 mm measured from the collimator extremity to the Eppendorf top (at this distance, the irradiance is approximately 1.8 mW/cm2 at 365 nm). The reaction volume was 20 µL. Samples were prepared by heat denaturing for 10 min at 95 °C a mixture of PAGE-purified ^{32}P-5'-end-labelled (.ca 100 Bq/µL, 22AG and Pu24T) and 10 µM of cold 22AG or Pu24T, for G4 folding, or 10 µM of cold 22AG and 12 µM of cold ihTel, for duplex DNA hybridization, in 10 mM lithium cacodylate buffer (pH 7.2) and 100 mM KCl in a total volume of 18 µL; G4 folding or hybridization into *ds* DNA was obtained by cooling the solutions at room temperature over a period of ~3 hours. Before irradiation, G4 or *ds* DNA was incubated with the compounds (20 µM) and the competitor at the appropriated concentrations for 30 min at room temperature to reach the total volume of 20 µL. After irradiation, the sample volumes were reduced and 5 µL of dye solution (80% formamide, 0.1 M EDTA, 0.1% xylene cyanol and 0.1% bromphenol blue (blue loading dye)) were added. A denaturing 15% polyacrylamide gel was run at 20 W. The gel was exposed to a storage phosphor screen and converted to a digital image with a Typhoon Trio variable-mode imager (GE Healthcare).

The yield of alkylation was defined as the ratio between the counts corresponding to the alkylation band and the sum of the counts corresponding to both the alkylation and the non-modified DNA bands, corrected by the background noise. The values given in % are the mean of at least three experiments.

DNA-Ligand covalent adduct purification

Freshly prepared ^{32}P-5'-end-labelled oligonucleotide (.ca 50000 Bq/µL) mixed with 10 µM cold DNA was incubated with 20 µM ligand and irradiated for 1 hour. The reaction mixture was purified by denaturing 15% PAGE and the gel was exposed to the phosphor screen. The alkylated bands were isolated, eluted from the gel, and precipitated. Centrifugation at 125000 rpm at 4 °C for 2 hours and supernatant removal allowed purified DNA recovery. Water was subsequently added to dissolve DNA and the radioactivity of samples was inferred by liquid scintillator analysis.

3'-Exonuclease digestion coupled with PAGE analysis

PAGE-purified alkylated DNA was digested with two different concentration of Phosphodiesterase I from Crotalus adamanteus venom (0.2 U/ µL, Tris-HCl 110 mM, NaCl 100 mM, MgCl2 15 mM, 50%v/v Glycerol). The alkylated products were incubated in 10 mM Tris-HCl buffer (pH = 8.0), in the presence of 2 mM $MgCl_2$ and 0.5 mg/mL transfer RNA with the 3'- Phosphodiesterase I from Crotalus adamanteus venom at 0.04 U/µL or 0.2 U/µL to a final volume of 5 µL for 25 min at 37 °C. The partial digestion of non-alkylated DNA was run with the enzyme at 0.01 and 0.007 U/µL concentration. For each adduct both treated and untreated samples were analysed by denaturing 20% polyacrylamide gel electrophoresis, along with treated and untreated unmodified DNA and a reference ladder. The main bands corresponding to an arrest of the exonuclease activity have been isolated, eluted, and ethanol precipitated. The migration of the fragments depends on the presence of the alkylating agents; therefore, each alkylated adduct was heated at 95 °C for 15 minutes to afford partial thermal reversion of the alkylation adduct. After precipitation, the dealkylated fragments were migrated on 20% denaturing gel.

Ladder: an amount of PAGE-purified unmodified DNA corresponding to a radioactivity 2 times higher than that of the most concentrated sample was added with 1 µL of reaction buffer and 1 µL of 0.01 U/µL or 0.07 U/µL exonuclease solutions, and if necessary, water was added to obtain a total volume of 5 µL. The solution was incubated 25 minutes at 37 °C, immediately cooled down to 0 °C and 3 µL of blue loading dye were added.

5X Reaction Buffer: Tris-HCl 250 mM, $MgCl_2$ 5 mM, pH 7.5 at 25 °C.

MALDI-ToF Mass Spectrometry Analysis

DNA alkylation product separation was performed on a C18 Uptisphere 300 Å, 150 x 2.1 mm column (Interchim) using a gradient of 0 % to 50 % CH_3CN in 10 mM TriEthylAmmonium Acetate buffer (TEAA) pH 7 at 50 °C. Chromatograms were obtained at 260 and 320 nm with a diode array detector and UV spectra were extracted for each chromatographic peak. The mass spectra of each purified peak were obtained in the negative mode on a Microflex MALDI-ToF mass spectrometer (Bruker), equipped with a 337-nm nitrogen laser and pulsed delay source extraction. The matrix was prepared by dissolving 3-hydroxypicolinic acid in 10 mM ammonium citrate buffer. A mixture of purified DNA sample (10 pmol; 1 µL) was added to matrix (1 µL) and spotted on a polished stainless target plate using the dried droplet method. Spectra were calibrated using reference oligonucleotides of known masses.

3'- and 5'-Exonuclease digestions coupled with MALDI-ToF MS analysis

Typically, 200 pmol of oligonucleotide (unmodified or adduct-containing sequences) in 20 µL of 0.02 M ammonium citrate buffer was digested at 37 °C by incubation with 0.03 unit of crotalus venom phosphodiesterase (3′→5′ exonuclease activity, pH 9) or bovine spleen phosphodiesterase (5′→3′ exonuclease activity, pH 5). At increasing periods of time, aliquots of 2 µL from the digestion mixture were collected and analyzed by MALDI-ToF-MS following the protocol mentioned above.

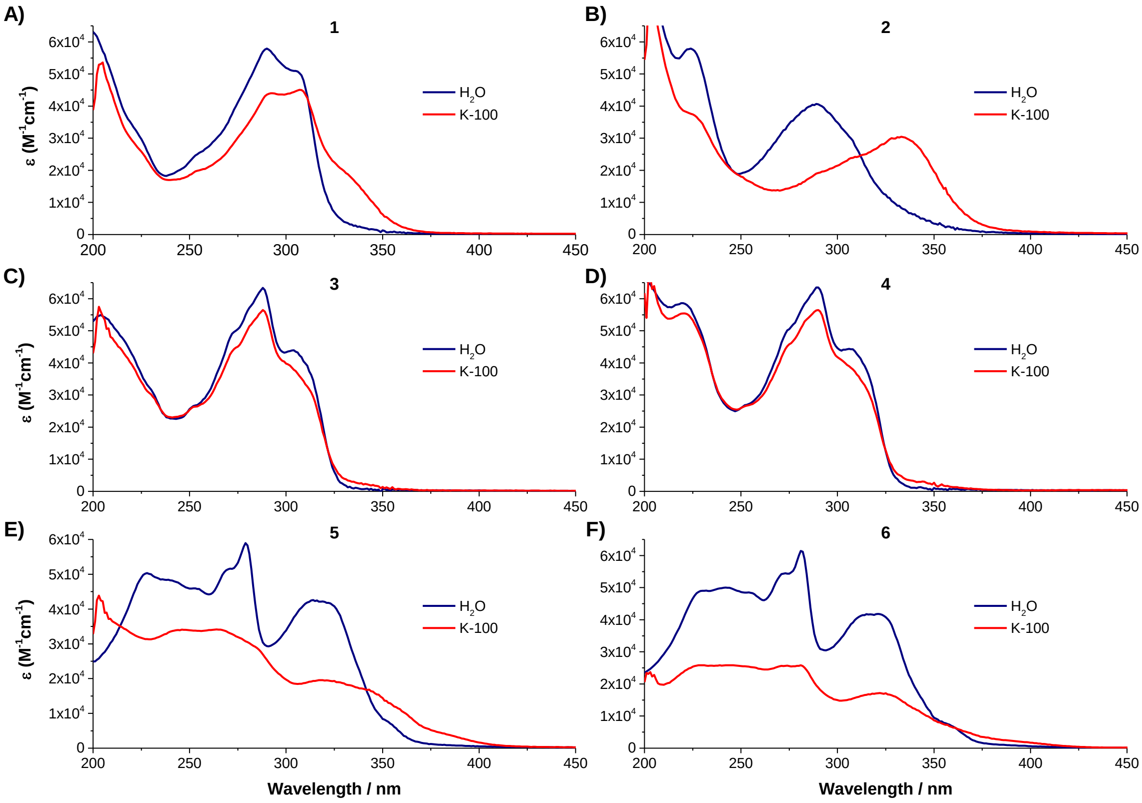

Figure S1. Ligand molar absorptivity in water and in 10 mM lithium cacodylate buffer (pH 7.2), 100 mM KCl. (A) **1**, (B) **2**, (C) **3**, (D) **4**, (E) **5**, and (F) **6**.

Table S1. Photophysical properties of the arylethynyl derivatives **1-6** in water and in buffer (KCl 100 mM, LiCaco 10 mM, pH 7.2).

Ligand	**Solvent**	**λ_{max} (nm)**	**ε_{max}(M^{-1} cm^{-1})**	**λ_{tail} (nm)**
**1**	Water	299	$5.24 \cdot 10^4$	340
	Buffer	320		360
**2**	Water	291	$6.28 \cdot 10^4$	340
	Buffer	320		380
**3**	Water	291	$5.80 \cdot 10^4$	327
	Buffer	291		346
**4**	Water	291	$6.28 \cdot 10^4$	327
	Buffer	291		355
**5**	Water	325	$4.08 \cdot 10^4$	380
	Buffer	325		410
**6**	Water	325	$4.08 \cdot 10^4$	380
	Buffer	325		416

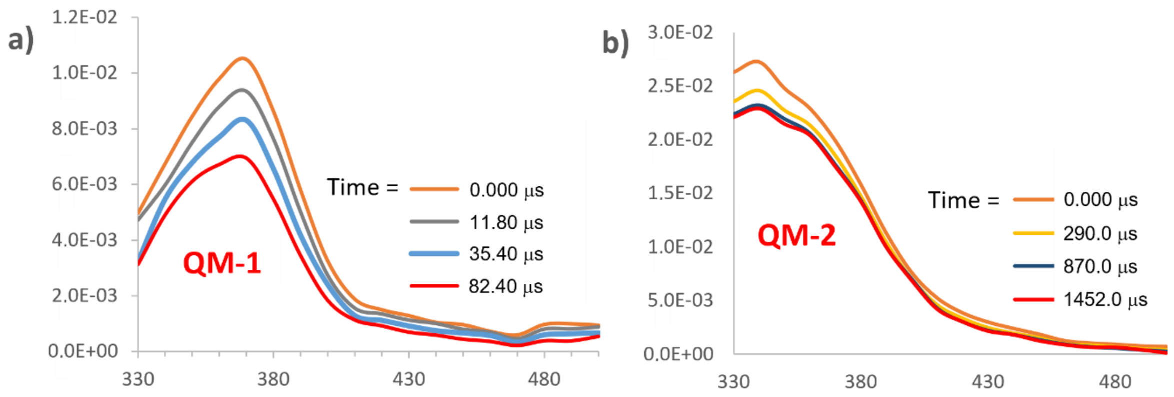

Figure S2. Transient absorption spectra of 10^{-4} M CH_3CN solutions of **1** (a) and **2** (b), in the presence of O_2, irradiated at 266 nm by LFP.

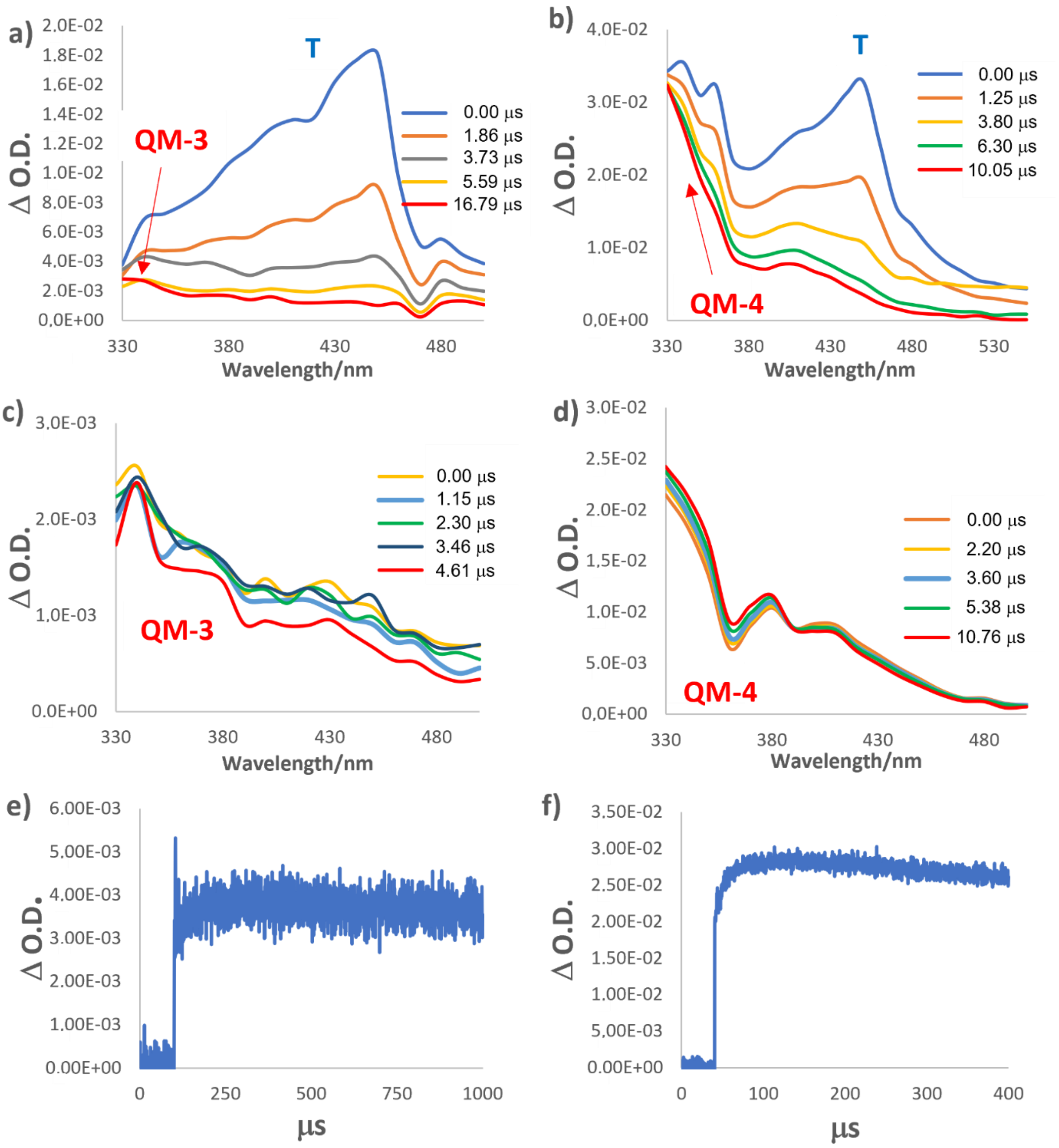

Figure S3. Transient absorption spectra of 10^{-4} M CH_3CN solutions of **3** (a) and **4** (b), purged by argon and irradiated at 266 nm by LFP. Transient absorption spectra of 10^{-4} M CH_3CN solutions of **3** (c) and **4** (d), in the presence of O_2. irradiated at 266 nm by LFP. (e) Decay traces monitored at 340 nm of CH_3CN solutions of **3** in the presence of O_2. (f) Decay traces monitored at 330 nm of CH_3CN solutions of **4** in presence of O_2.

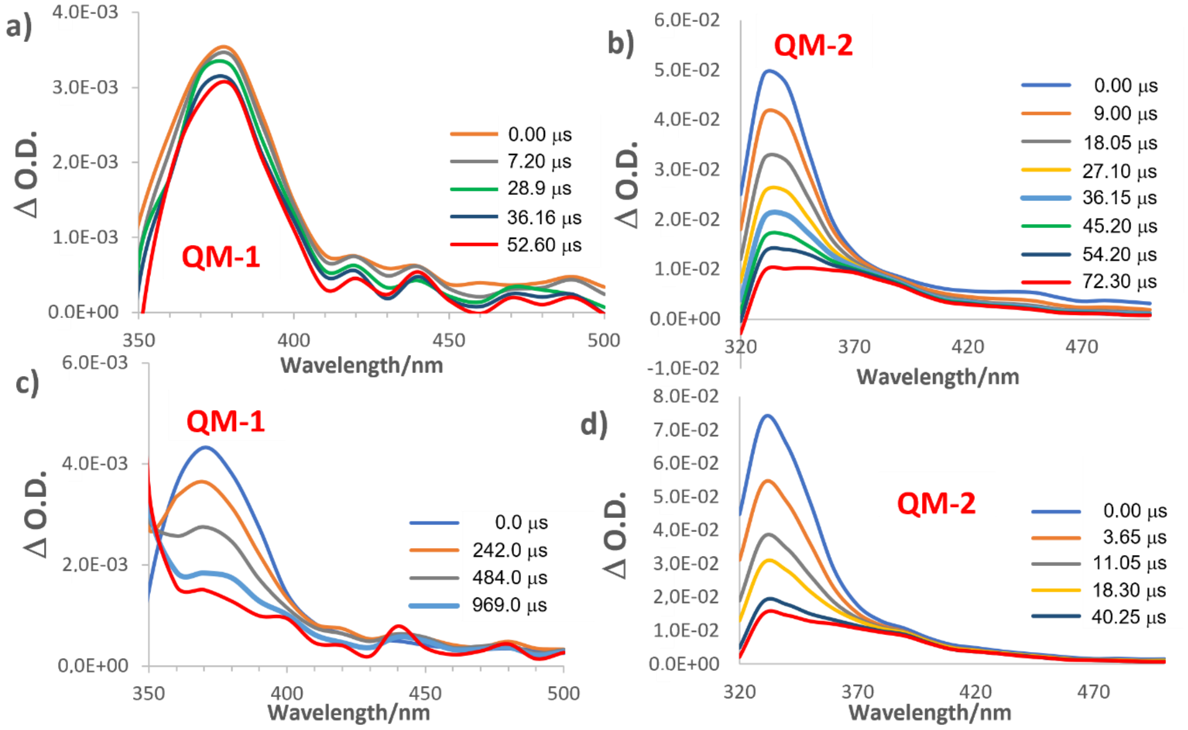

Figure S4. Transient absorption spectra of 10^{-4} M $CH_3CN:H_2O$ (1:1) solutions of **1** (a) and **2** (b), purged by argon and irradiated at 266 nm by LFP. Transient absorption spectra of 10^{-4} M $CH_3CN:H_2O$ (1:1) solutions of **1** (c) and **2** (d), in the presence of O_2, irradiated at 266 nm by LFP.

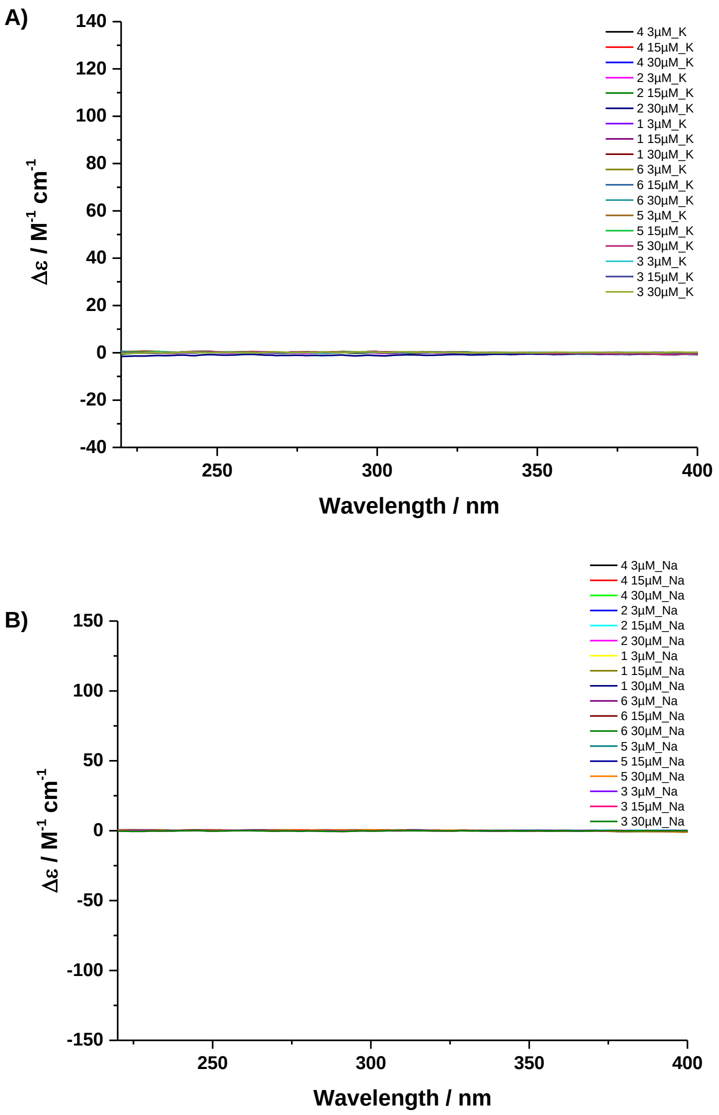

Figure S5. CD spectra of compounds **1-6** at 3, 15, and 30 µM in (A) 10 mM Li Caco buffer (pH 7.2), 100 mM KCl and (B) 10 mM lithium cacodylate buffer (pH 7.2), 100 mM NaCl.

Table S2. Isoelliptic points identified by CD titration performed in the presence of 22AG both in K- and Na-rich buffer and the synthesized ligands.

Ligand	Isoelliptic point$_{22AG.K}$ (nm)	Isoelliptic point$_{22AG.Na}$ (nm)
**1**	240, 260, 302	249, 274, 285, 298
**2**	262, 297	251, 273, 298
**3**	240, 267, 290, 336	250, 271, 292, 297, 323, 340
**4**	240	251, 269, 289, 322, 340
**5**	267	261
**6**	278	250, 275, 304

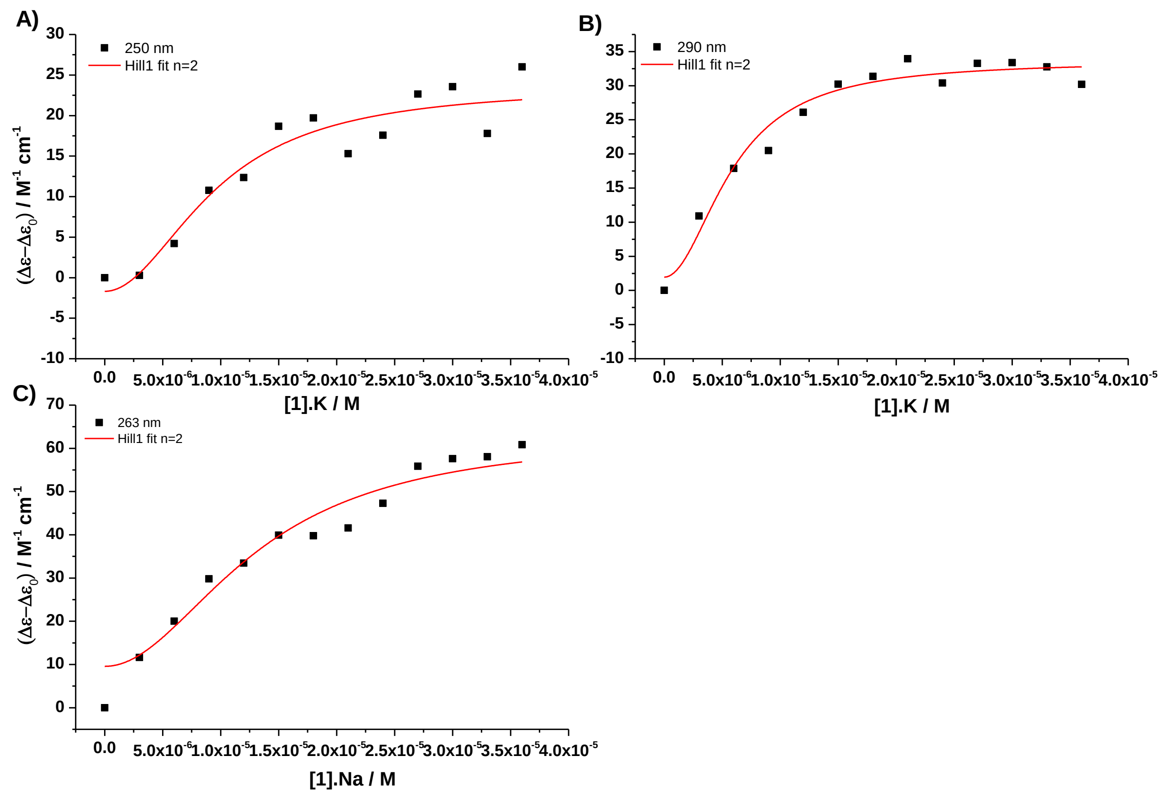

Figure S6. Titration data of compound **1** derived by taking a vertical cross section at (A) 250 nm, (B) 290 nm and (C) 263 nm in K-rich (A and B) and Na-rich (C) buffer respectively in Figure 5. The data have been normalized by subtraction of pure 22AG spectrum in (A-B) in 10 mM Li Caco buffer (pH 7.2), 100 mM KCl, and (C) in 10 mM Li Caco buffer (pH 7.2), 100 mM NaCl, respectively. The solid line is the theoretical fit of the data using the Hill equation.

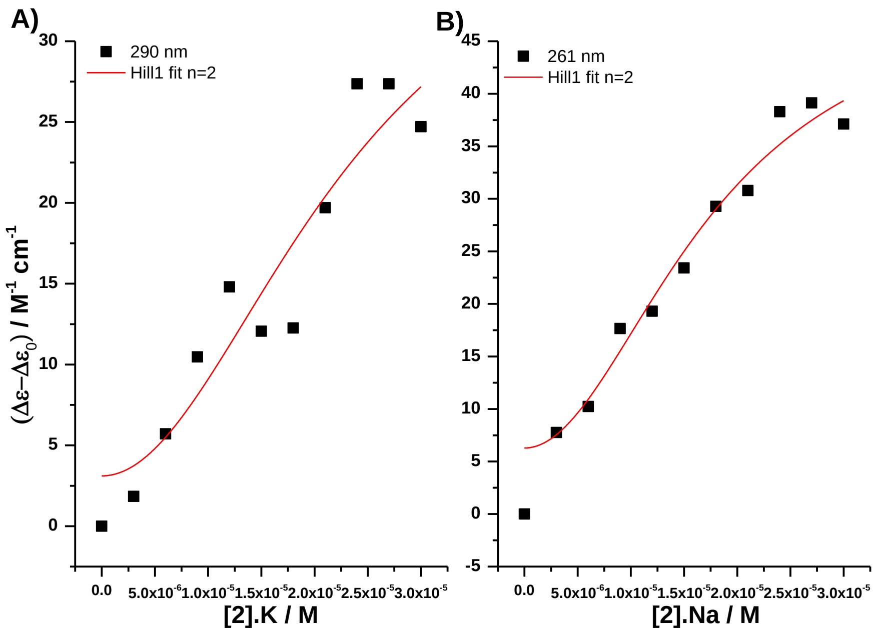

Figure S7. Titration data of compound **2** derived by taking a vertical cross section at (A) 290 nm and (B) 261 nm in K-rich and Na-rich buffer respectively in Figure 5. The data have been normalized by subtraction of pure 22AG spectrum in (A) in 10 mM Li Caco buffer (pH 7.2), 100 mM KCl, and (B) in 10 mM Li Caco buffer (pH 7.2), 100 mM NaCl, respectively. The solid line is the theoretical fit of the data using the Hill equation.

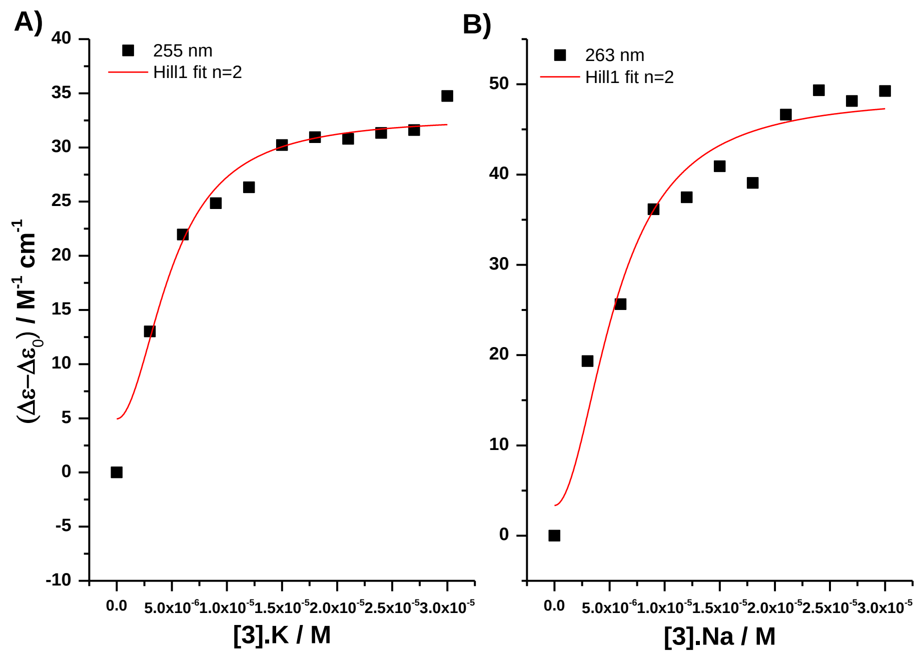

Figure S8. Titration data of compound **3** derived by taking a vertical cross section at (A) 255 nm and (B) 263 nm in K-rich and Na-rich buffer respectively in Figure 5. The data have been normalized by subtraction of pure 22AG spectrum in (A) in 10 mM Li Caco buffer (pH 7.2), 100 mM KCl, and (B) in 10 mM Li Caco buffer (pH 7.2), 100 mM NaCl, respectively. The solid line is the theoretical fit of the data using the Hill equation.

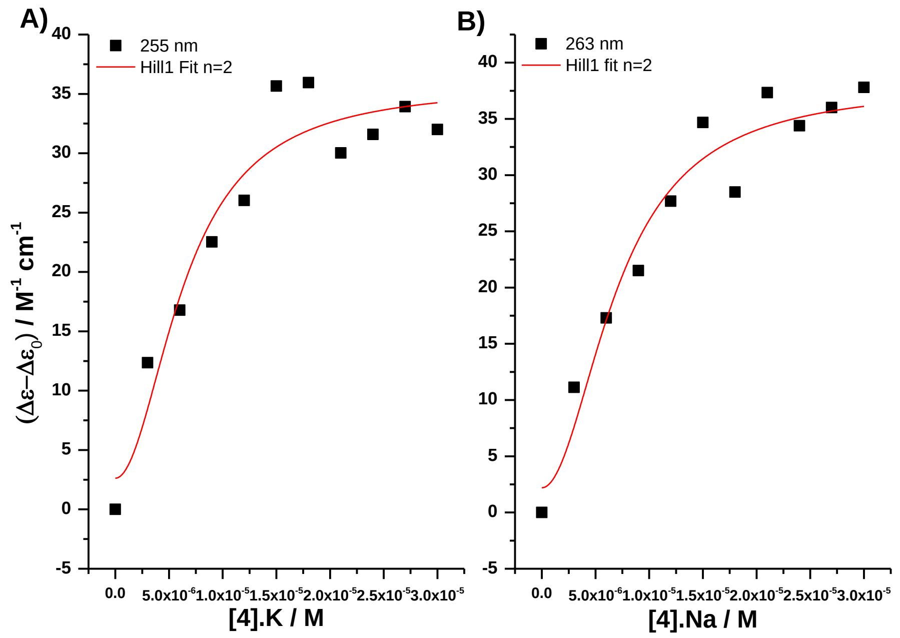

Figure S9. Titration data of compound **4** derived by taking a vertical cross section at (A) 255 nm and (B) 263 nm in K-rich and Na-rich buffer respectively in Figure 5. The data have been normalized by subtraction of pure 22AG spectrum in (A) in 10 mM Li Caco buffer (pH 7.2), 100 mM KCl, and (B) in 10 mM Li Caco buffer (pH 7.2), 100 mM NaCl, respectively. The solid line is the theoretical fit of the data using the Hill equation.

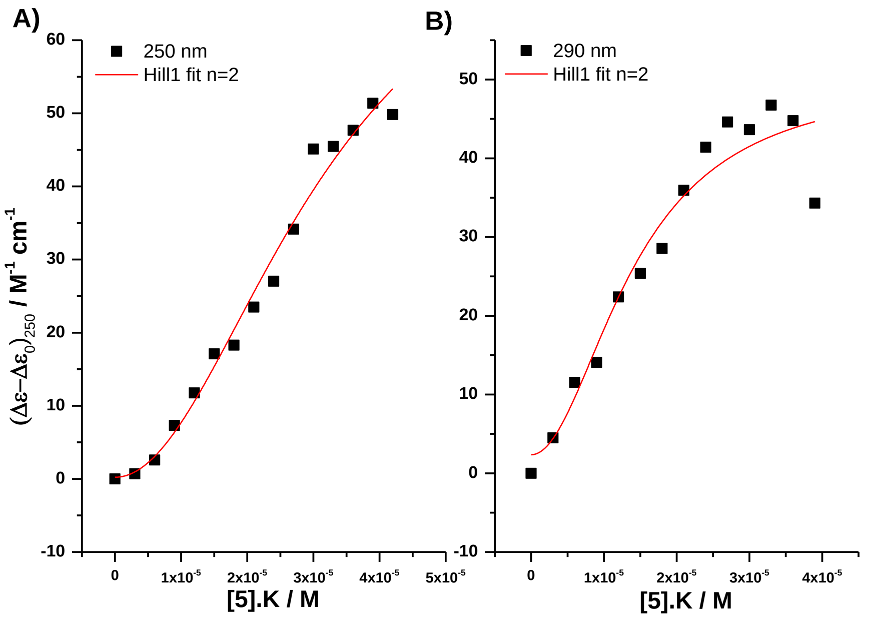

Figure S10. Titration data of compound **5** derived by taking a vertical cross section at (A) 250 nm, and (B) 290 nm in K-rich (A) and Na-rich (B) buffer respectively in Figure 5. The data have been normalized by subtraction of pure 22AG spectrum in (A) in 10 mM Li Caco buffer (pH 7.2), 100 mM KCl, and (B) in 10 mM Li Caco buffer (pH 7.2), 100 mM NaCl, respectively. The solid line is the theoretical fit of the data using the Hill equation.

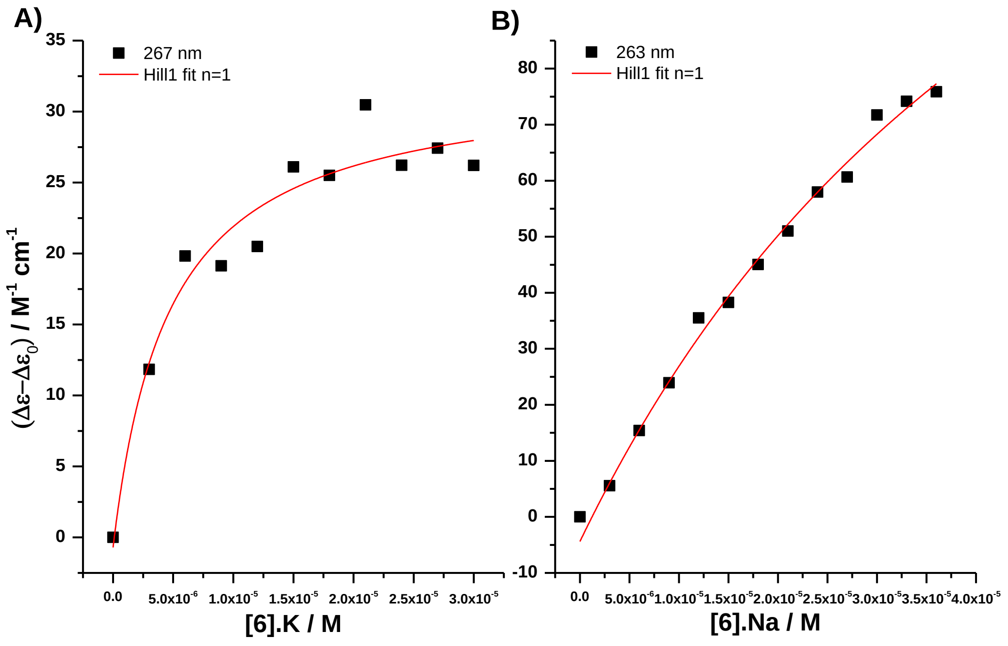

Figure S11. Titration data of compound **6** derived by taking a vertical cross section at (A) 267 nm, and (B) 263 nm in K-rich (A) and Na-rich (B) buffer respectively in Figure 5. The data have been normalized by subtraction of pure 22AG spectrum in (A) in 10 mM Li Caco buffer (pH 7.2), 100 mM KCl, and (B) in 10 mM Li Caco buffer (pH 7.2), 100 mM NaCl, respectively. The solid line is the theoretical fit of the data using the Hill equation.

Table S3. Binding constants obtained by global fitting using a non-linear regression with the Hill equation at appropriate wavelength (maximum identified by the ICD spectrum for each compound). Calculated values of binding constants are obtained from duplicates.

Ligand	K_d.K (M)	K_d.Na (M)
**1**	$9.6(\pm0.6)\cdot10^{-6}{}_{(\lambda250)}$	$13.3(\pm0.3)\cdot10^{-6}{}_{(\lambda263)}$
	$5.9(\pm0.7)\cdot10^{-6}{}_{(\lambda290)}$	
**2**	$23.3(\pm15.3)\cdot10^{-6}{}_{(\lambda290)}$	$18.1(\pm3.5)\cdot10^{-6}{}_{(\lambda261)}$
**3**	$5.0(\pm2.8)\cdot10^{-6}{}_{(\lambda255)}$	$5.6(\pm2.2)\cdot10^{-6}{}_{(\lambda263)}$
**4**	$6.5(\pm1.0)\cdot10^{-6}{}_{(\lambda255)}$	$7.1(\pm3.6)\cdot10^{-6}{}_{(\lambda263)}$
**5**	$31.9(\pm21.8)\cdot10^{-6}{}_{(\lambda250)}$	-
	$14.2(\pm3.2)\cdot10^{-6}{}_{(\lambda290)}$	-
**6**	$4.6(\pm1.0)\cdot10^{-6}{}_{(\lambda267)}$	$58.8(\pm1.6)\cdot10^{-6}{}_{(\lambda263)}$

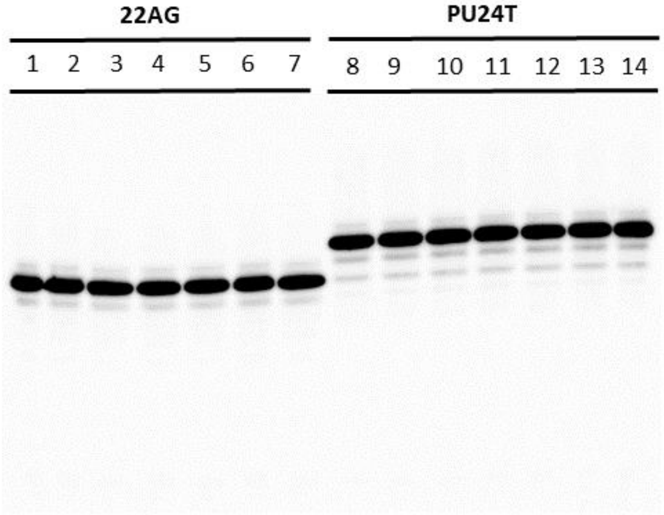

Figure S12. Control experiments showing the effect in the absence of irradiation (Lanes 1 and 8) and 22AG irradiation after 0.25, 0.5, 1, 2, 4, and 8 hrs at 365 nm (Lane 2, 3, 4, 5, 6, and 7) and Pu24T irradiation after 0.25, 0.5, 1, 2, 4, and 8 hrs at 365 nm (Lane 9, 10, 11, 12, 13, and 14).

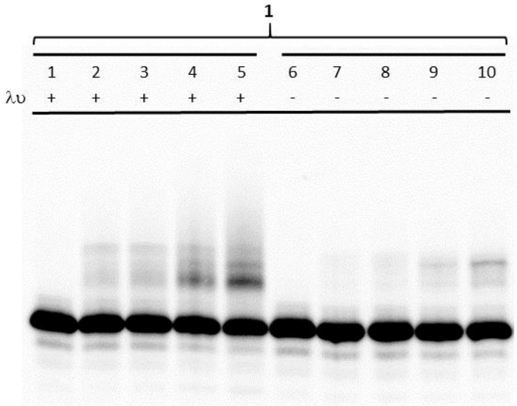

Figure S13. Denaturing gel electrophoresis (15% acrylamide) of the alkylation products of 22AG (10 µM) in the presence of K^+ buffer (100 mM) with **1** (10, 20, 50, and 100 µM). Lanes 1, 6, 7, 8, 9, and 10 are control experiments showing the effect in the absence of irradiation and in the presence of 0, 10, 20, 50, and 100 µM of **1** (Lanes 6, 7, 8, 9, and 10) and 60 minute 22AG irradiation at 365 nm without ligand (Lane 1). Lanes 2, 3, 4, and 5 show the results of irradiation at 365 nm during 60 minutes in the presence of 1, 2, 5, and 10 equivalents of **1**.

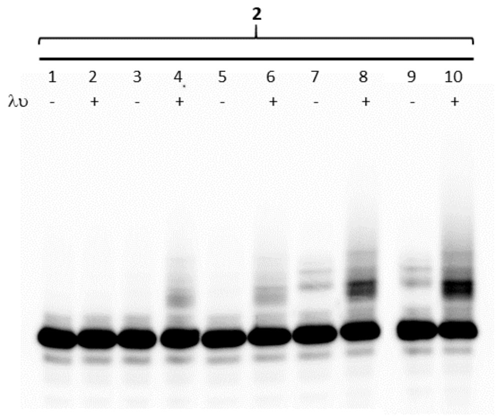

Figure S14. Denaturing gel electrophoresis (15% acrylamide) of the alkylation products of 22AG (10 µM) in the presence of K^+ buffer (100 mM) with **2** (10, 20, 50, and 100 µM). Lanes 1, 2, 3, 5, 7, and 9 are control experiments showing the effect in the absence of irradiation and in the presence of 0, 10, 20, 50, and 100 µM of **2** (Lanes 1, 3, 5, 7, and 9) and 60 minute 22AG irradiation at 365 nm without ligand (Lane 2). Lanes 4, 6, 8, and 10 show the results of irradiation at 365 nm during 60 minutes in the presence of 1, 2, 5, and 10 equivalents of **2**.

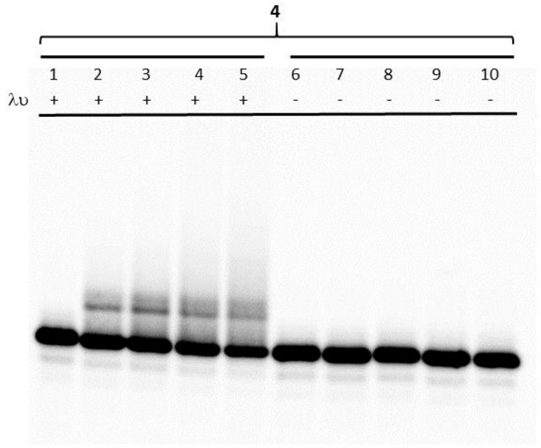

Figure S15. Denaturing gel electrophoresis (15% acrylamide) of the alkylation products of 22AG (10 µM) in the presence of K^+ buffer (100 mM) with **4** (10, 20, 50, and 100 µM). Lanes 1, 6, 7, 8, 9, and 10 are control experiments showing the effect in the absence of irradiation and in the presence of 0, 10, 20, 50, and 100 µM of **4** (Lanes 6, 7, 8, 9, and 10) and 60 minute 22AG irradiation at 365 nm without ligand (Lane 1). Lanes 2, 3, 4, and 5 show the results of irradiation at 365 nm during 60 minutes in the presence of 1, 2, 5, and 10 equivalents of **4**.

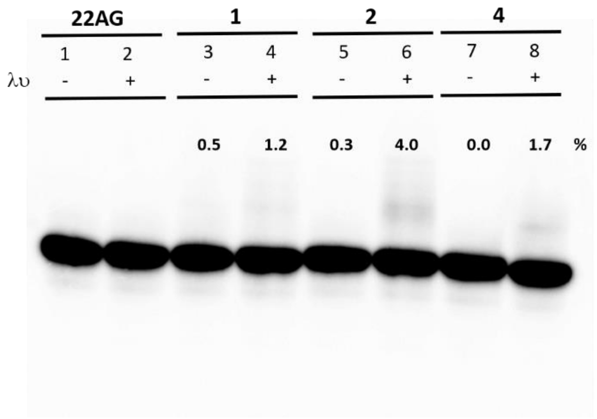

Figure S16. Denaturing gel electrophoresis (15% acrylamide) of the irradiation of the duplex DNA constituted by 22AG and ihTel in the presence of K^+-rich buffer by compounds **1**, **2** and **4**. Lanes 1, 2, 3, 4, 5, 6, 7 and 8 are control experiments that show the effect of no irradiation (lane 1, 3, 5 and 7) and irradiation at 365 nm (lanes 2, 4, 6 and 8).

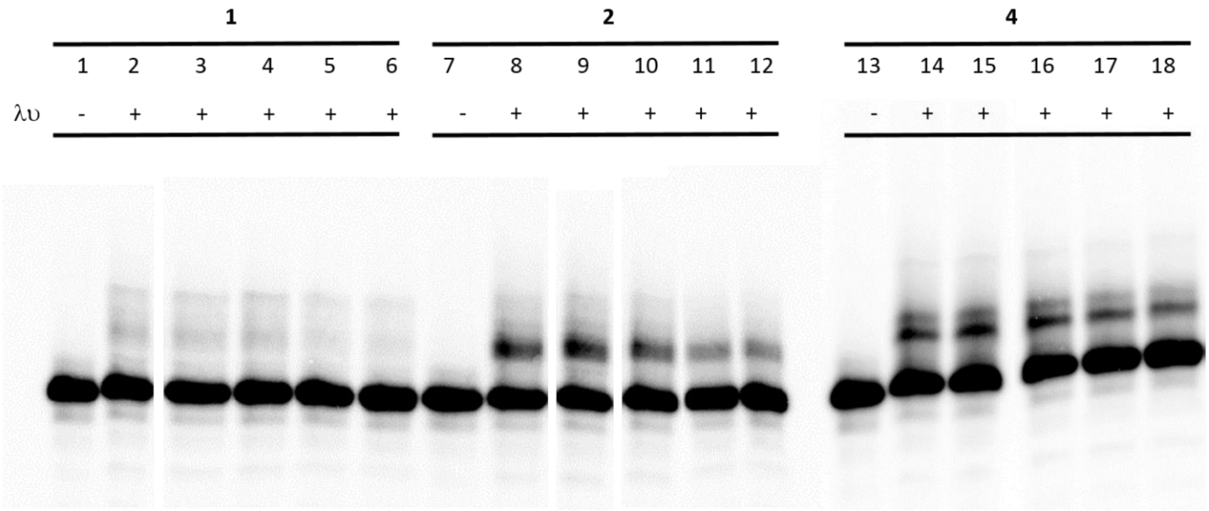

Figure S17. Denaturing gel electrophoresis (15% acrylamide) of the alkylation products of 22AG (10 µM) in K^+-buffer (100 mM) in the presence of **1**, **2**, and **4** (20 µM) in the absence or in the presence of increasing concentration of ds26 competitor (0, 10, 20, 50, and 100 µM). Lanes 1, 7, and 13 are control experiments showing the effect in the absence of irradiation and 22AG irradiation at 365 nm in the presence of 0, 10, 20, 50, and 100 µM ds26 competitor (Lanes 2, 3, 4, 5, and 6 for **1**; Lanes 8, 9, 10, 11 and 12 for **2**; Lanes 14, 15, 16, 17, and 18 for **4**).

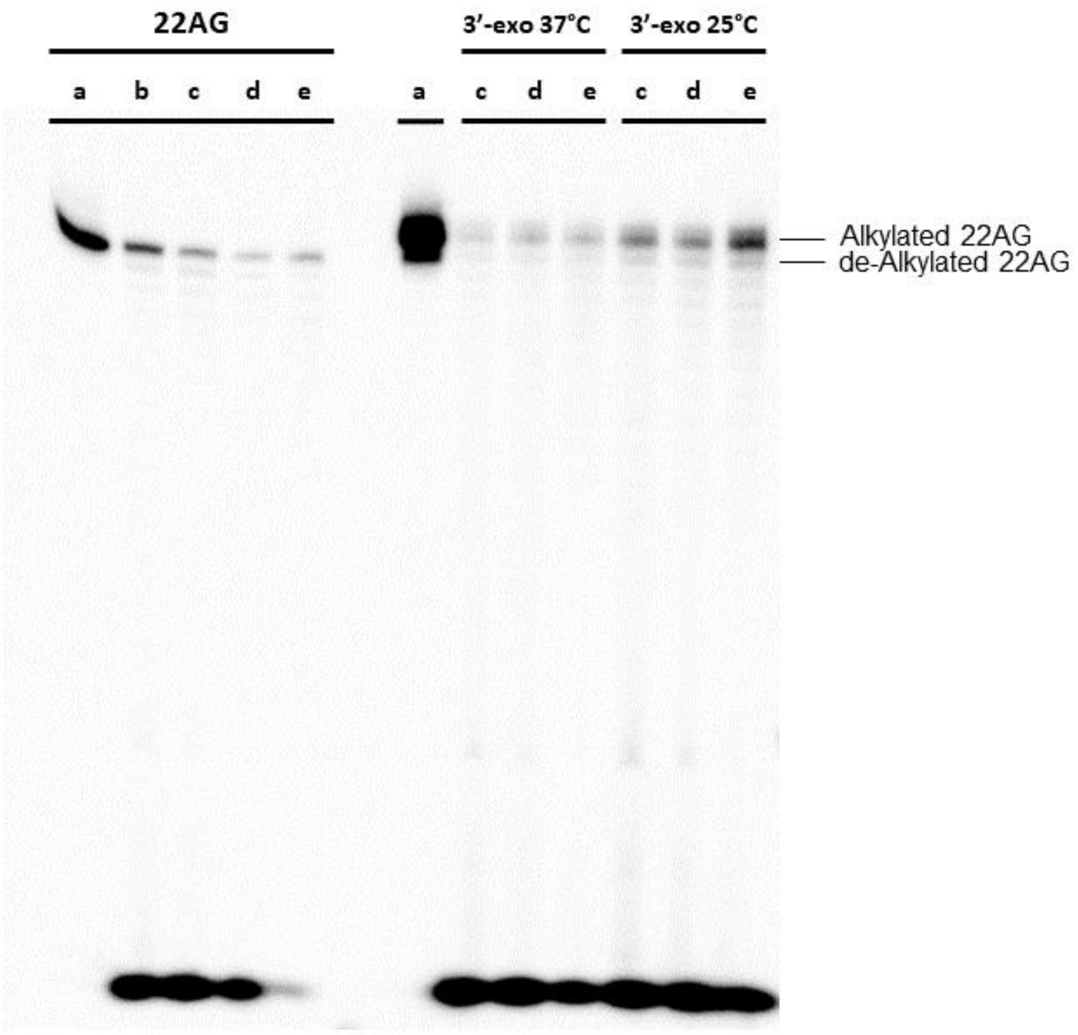

Figure S18. Denaturing gel electrophoresis of alkylated products of 22AG in K-rich buffer. 3'-Exonuclease digestion of the band obtained by alkylation with **2**: enzyme units/µL lane a, 0; lane b, 0.007; lane c, 0.01; lane d, 0.02; lane e, 0.2. Alkylated 22AG and de-alkylated 22AG bands are shown.

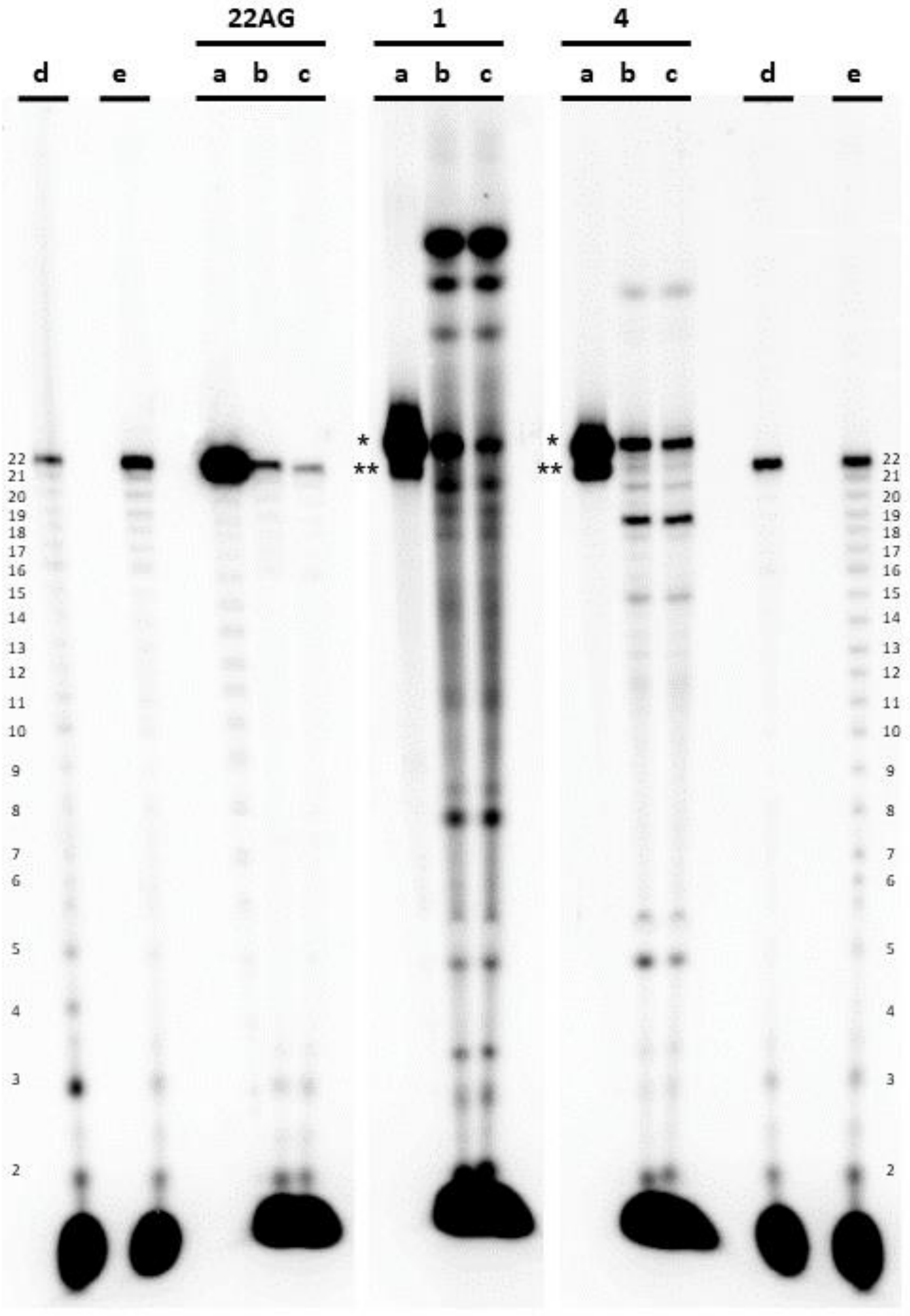

Figure S19. Denaturing gel electrophoresis of alkylated products of 22AG in K-rich buffer. 3'-Exonuclease digestion of the band obtained by alkylation with **1** and **4**: enzyme units/µL lane a, 0; lane b, 0.04; lane c, 0.2; 22AG corresponds to control of non-alkylated G4 structure; DNA ladders are obtained treating 22AG with different dilution of 3'-exonuclease: lane d, 0.01; lane e, 0.007. *: Alkylated 22AG and **: 22AG

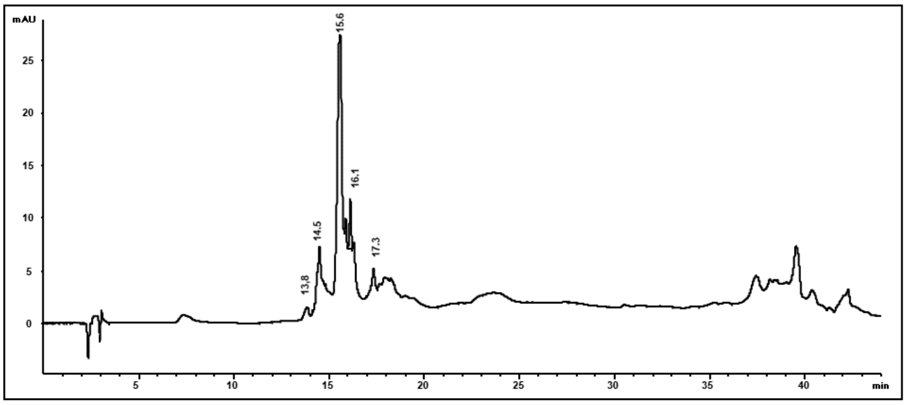

Figure S20. Chromatographic profile of the crude reaction mixture obtained after irradiation of 22AG with the Product **2** using a gradient of 0 % to 50 % CH_3CN in 10 mM TEAA buffer at 50 °C detected at 320 nm.

a)

b)

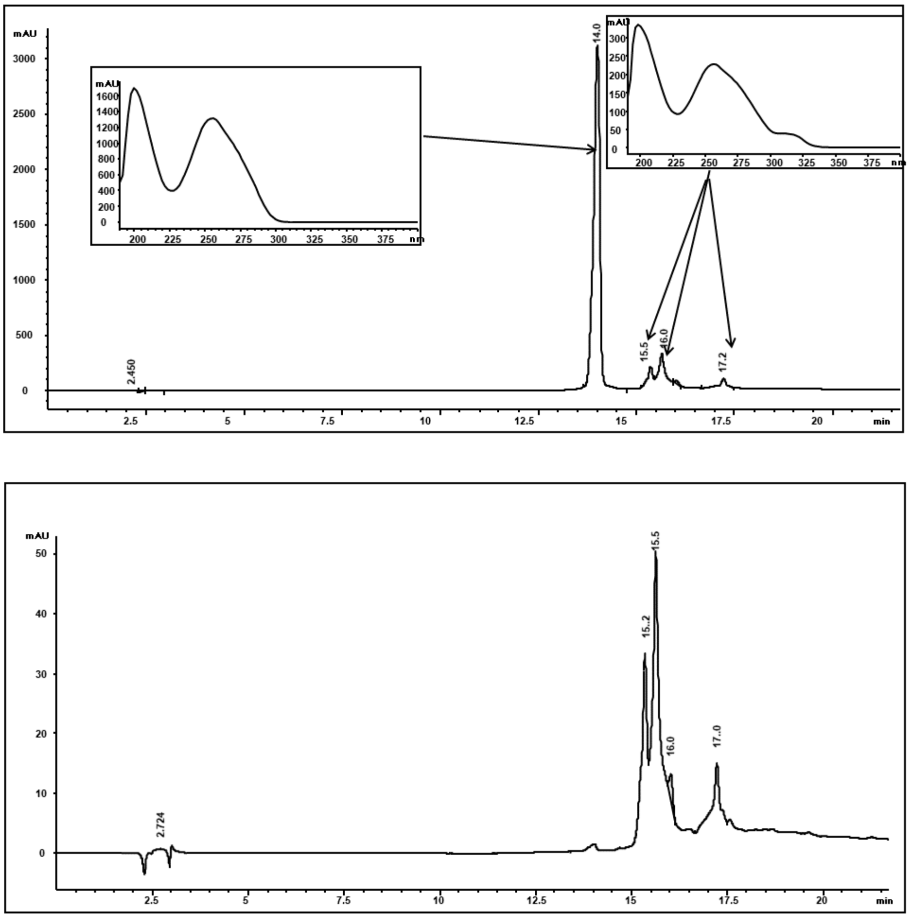

Figure S21. (a) Chromatographic profile of the crude reaction mixture obtained after irradiation of 22AG with the compound **4** using a gradient of 0 % to 50 % CH_3CN in 10 mM TEAA buffer at 50 °C detected at 260 nm. (b) Chromatographic profile of the crude reaction mixture obtained after irradiation of 22AG with the compound **4** detected at 320 nm.

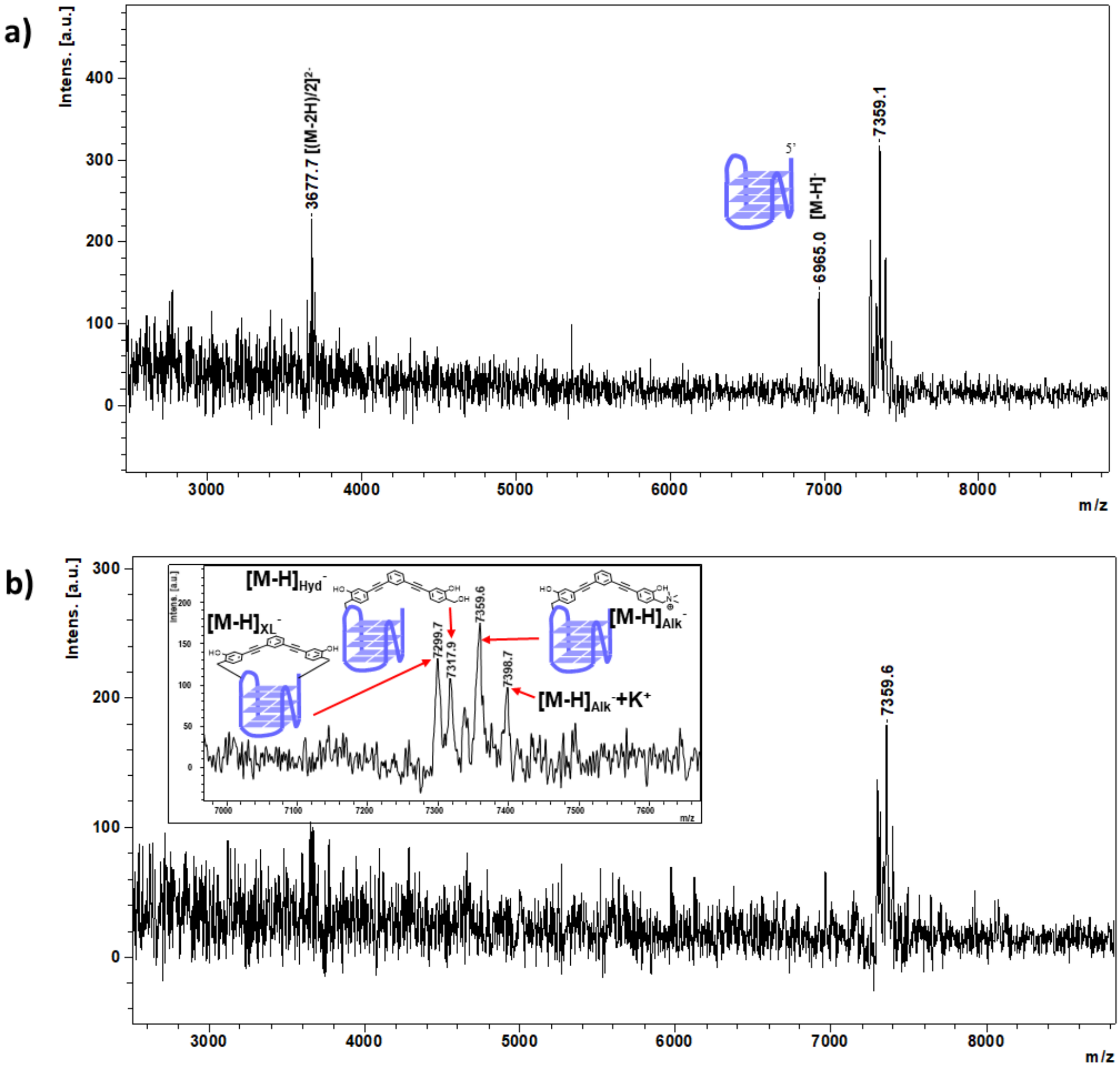

Figure S22. (a) MALDI-ToF mass spectrum of the collected product eluted at 15.2 min obtained after irradiation of 22AG and compound **4**. (b) MALDI-ToF mass spectrum of the collected product eluted at 15.5 min obtained after irradiation of 22AG and compound **4** and schematic representation of the covalent products.

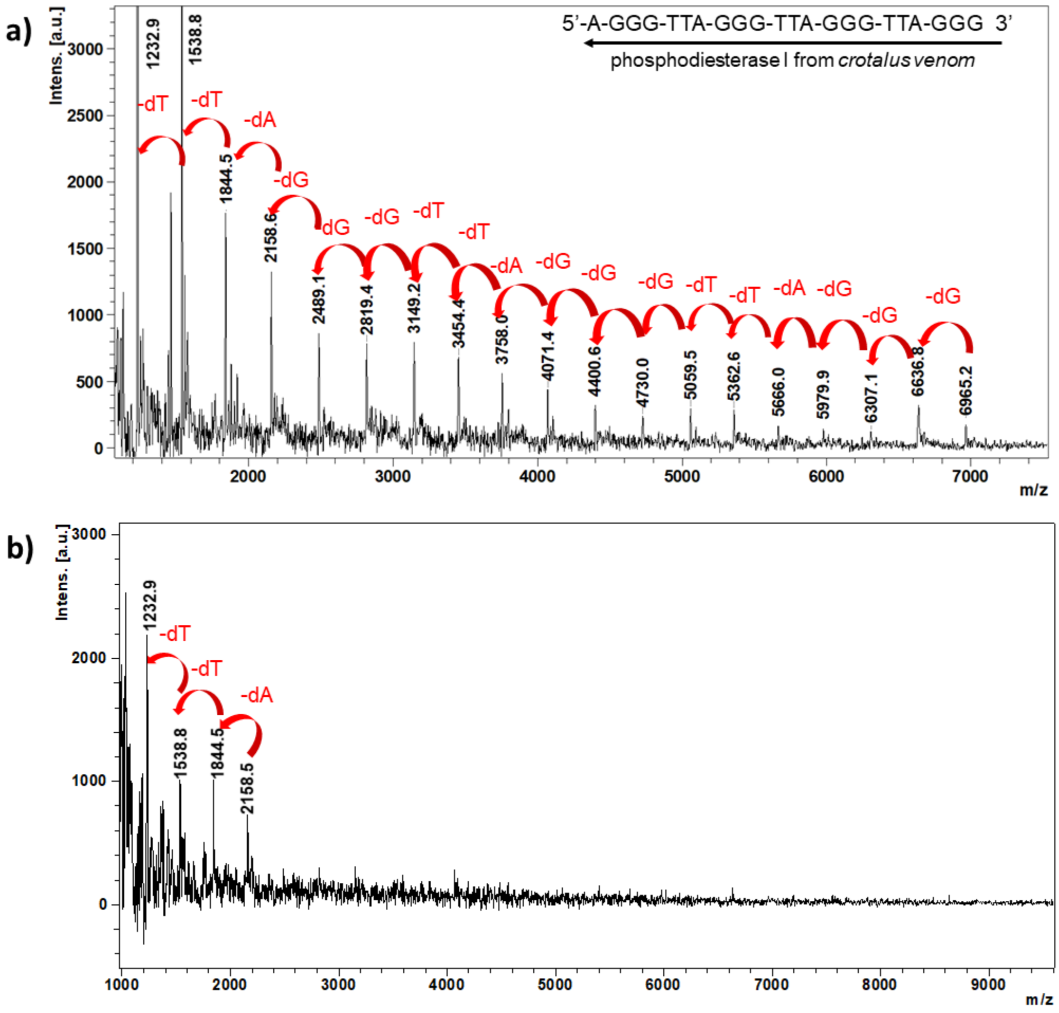

Figure S23. MALDI-ToF Mass Spectra of unmodified oligonucleotide 22AG (2 picomoles) digested at 37 °C by incubation with 0.03 unit of crotalus venom phosphodiesterase I (3′→5′ exonuclease activity) (a) for 40 minutes and (b) for 2 hours.

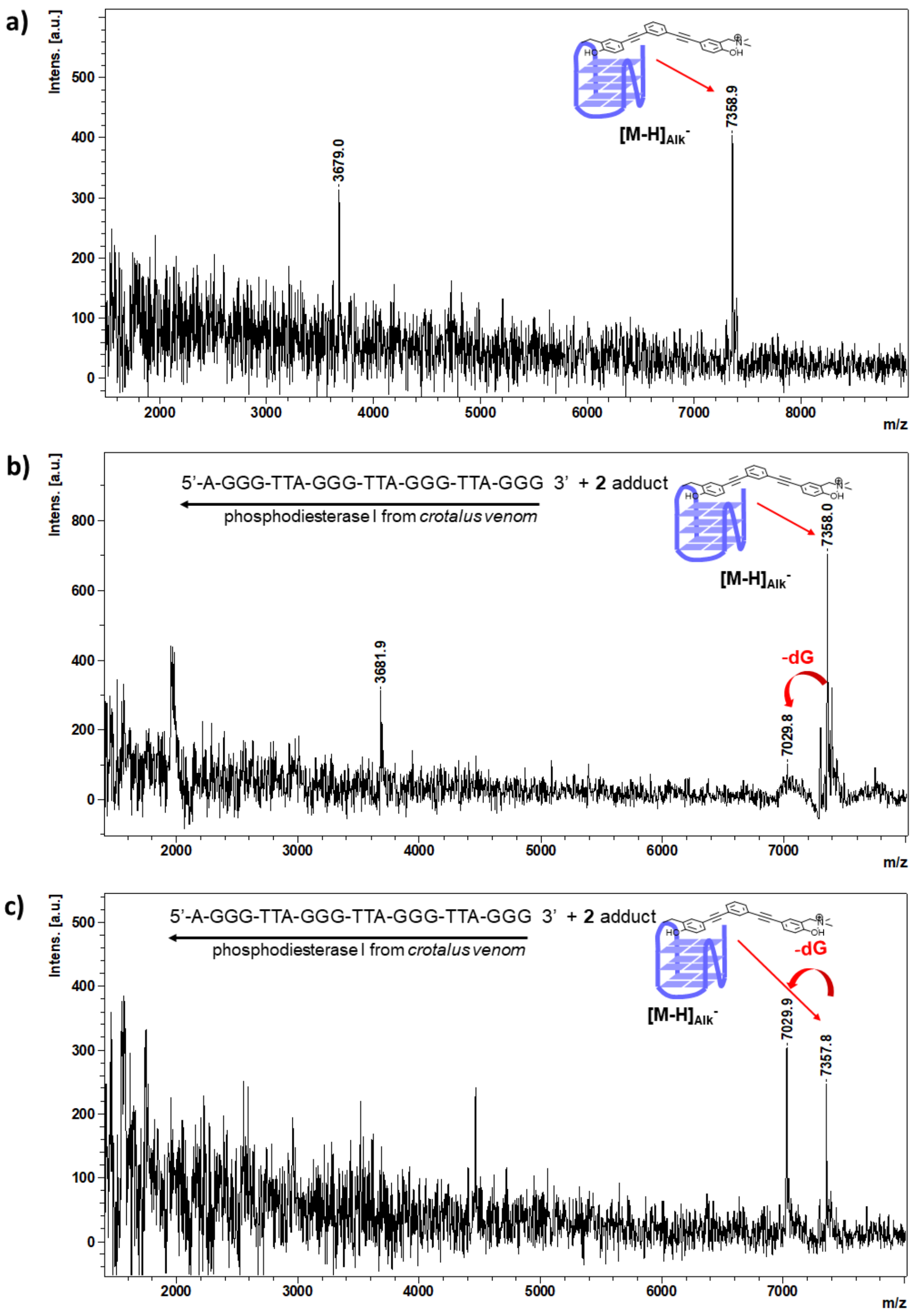

Figure S24. (a) MALDI-ToF mass spectrum of the purified mono-alkylated product obtained after irradiation of 22AG in presence of compound **2**. MALDI-ToF mass spectra of 2 picomoles of mono-alkylated product digested for (b) 10 minutes or (c) 6 h by phosphodiesterase I from crotalus venom.

HPLC Purity Analysis

1. (rT= 8.7 min, 98%)

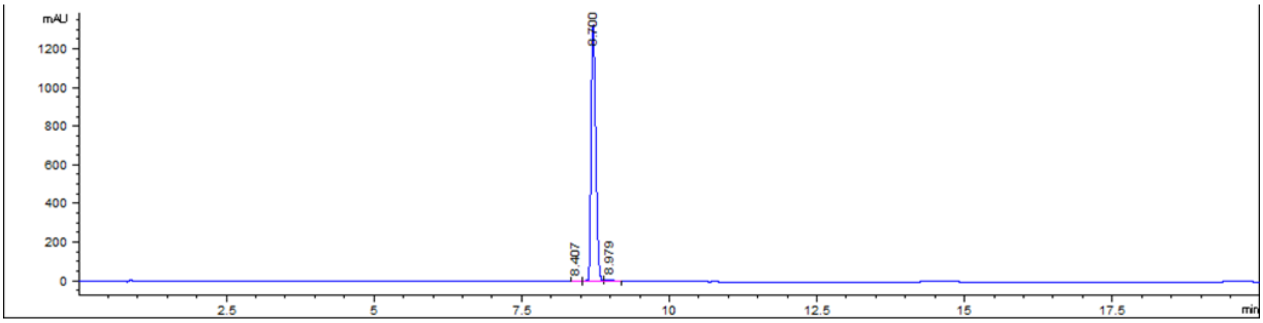

2. (rT= 8.8 min, 95%)

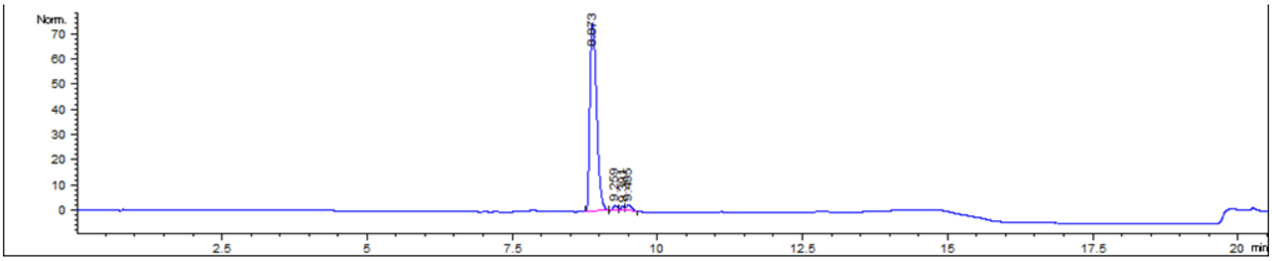

3. (rT= 8.9 min, 99%)

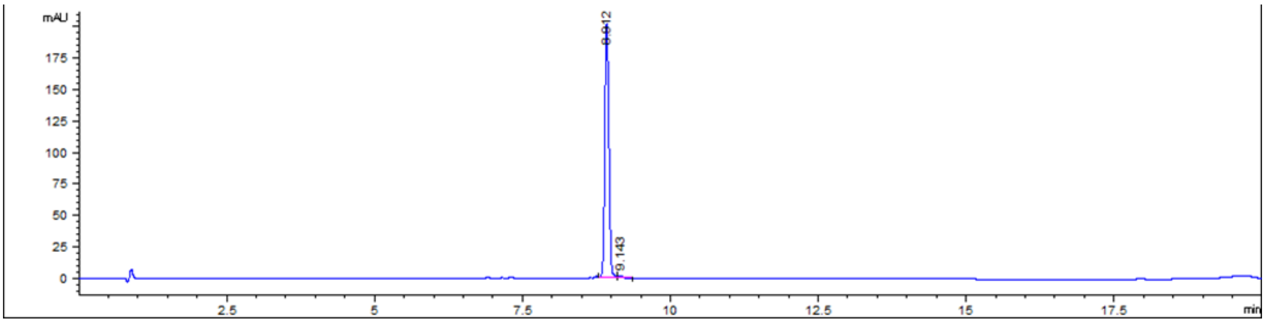

4. (rT= 9.6 min, 99%)

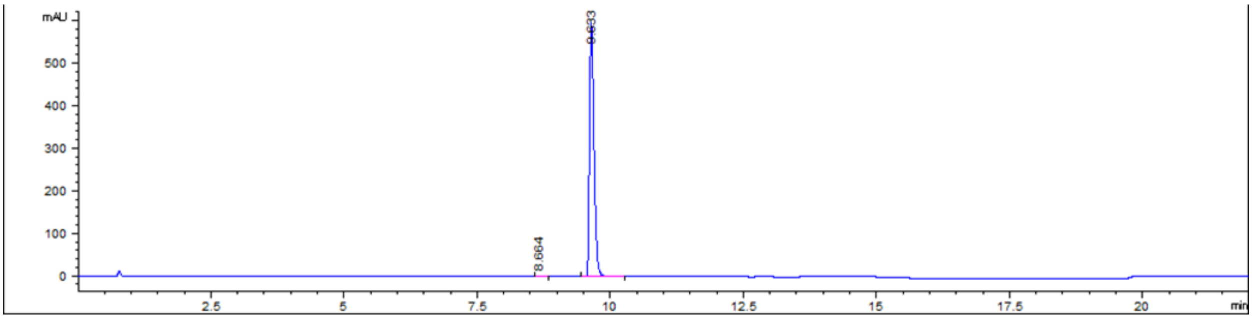

5. (rT= 10.5 min, 95%)

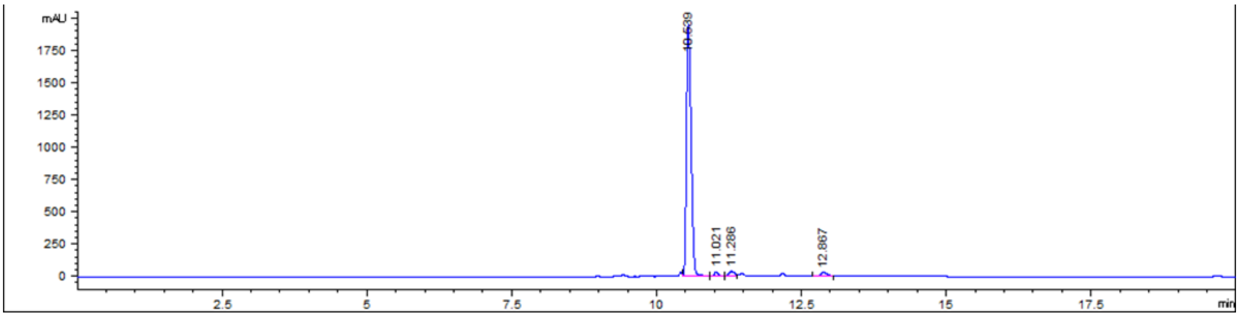

6. (rT= 10.7 min, 97%)

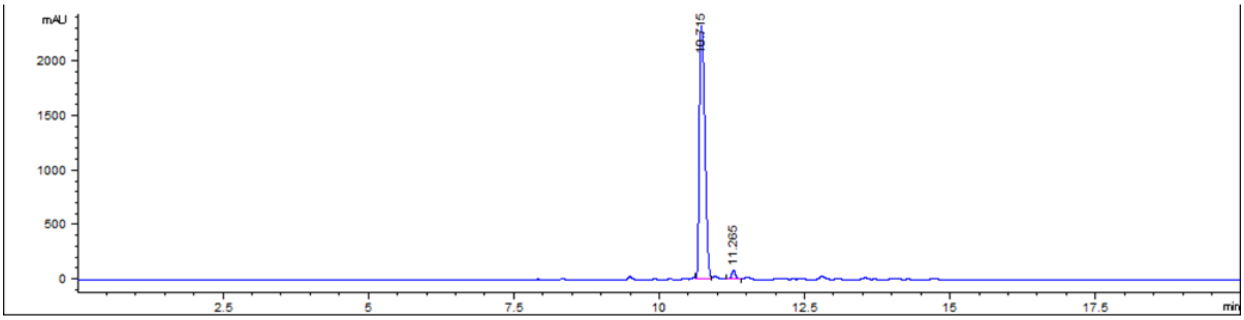

16. (rT = 9.56 min, 98%)

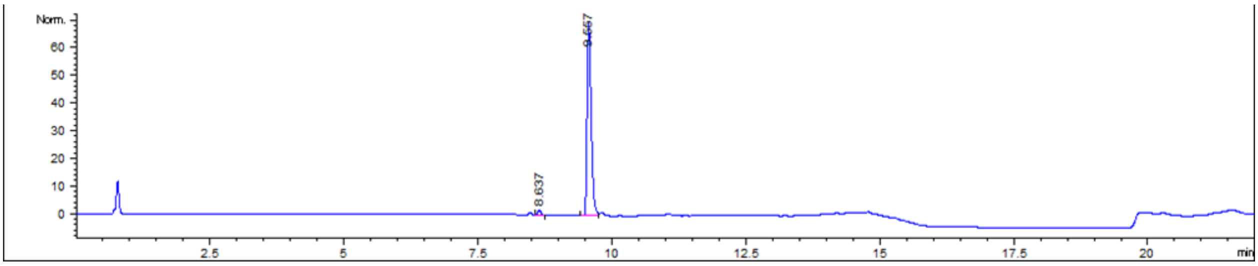

17. (rT= 10.5 min, 95%)

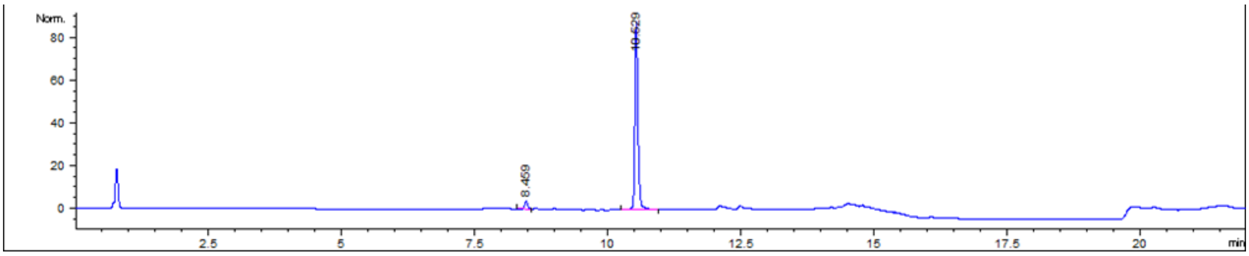

19. (rT = 9.7 min, 95%)

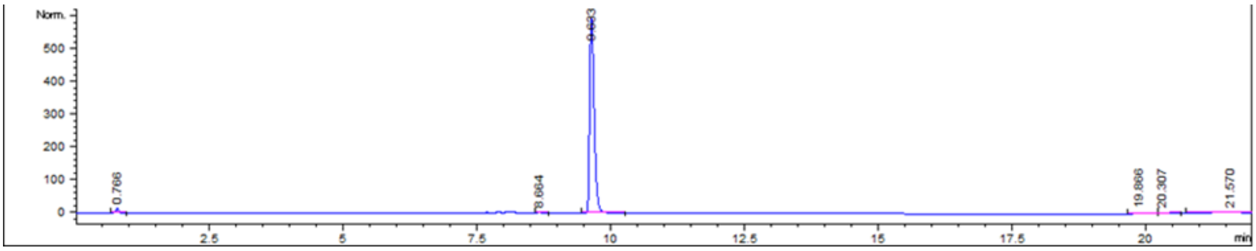

20. (rT = 10.7 min, 98%)

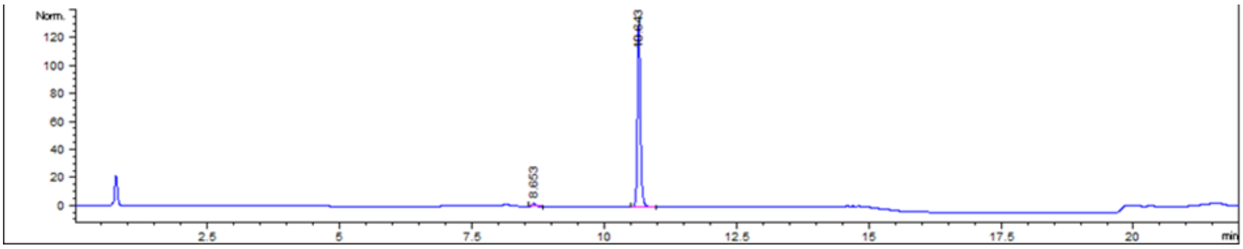

21. (rT = 12.5 min, 97%).

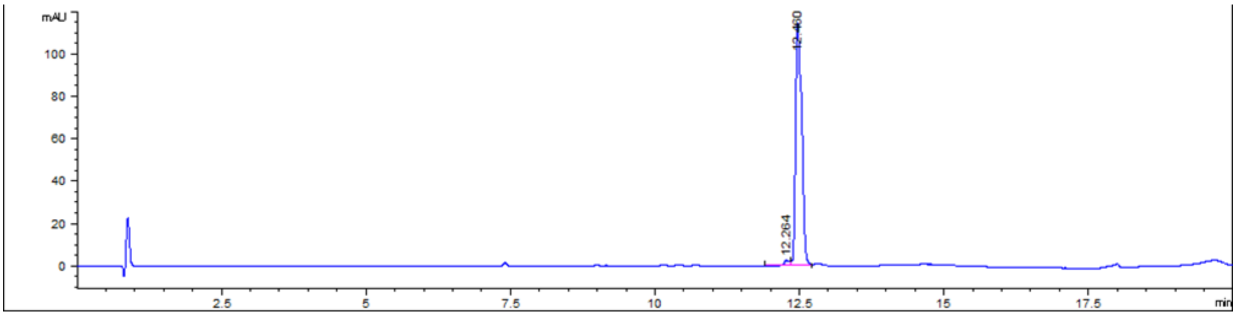

^{1}H-NMR and ^{13}C-NMR Characterizations

1. ^{1}H-NMR (300 MHz, $CDCl_3$)

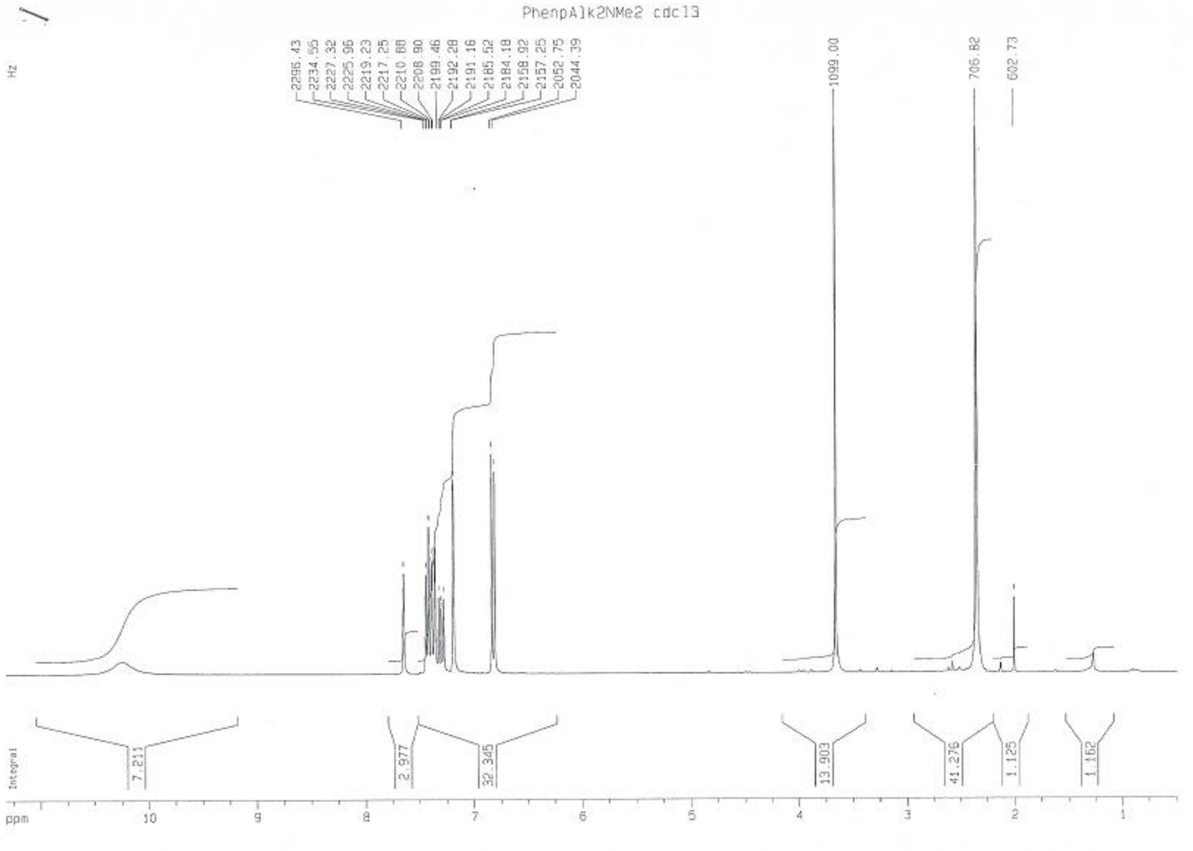

1. ^{13}C-NMR (75 MHz, $CDCl_3$)

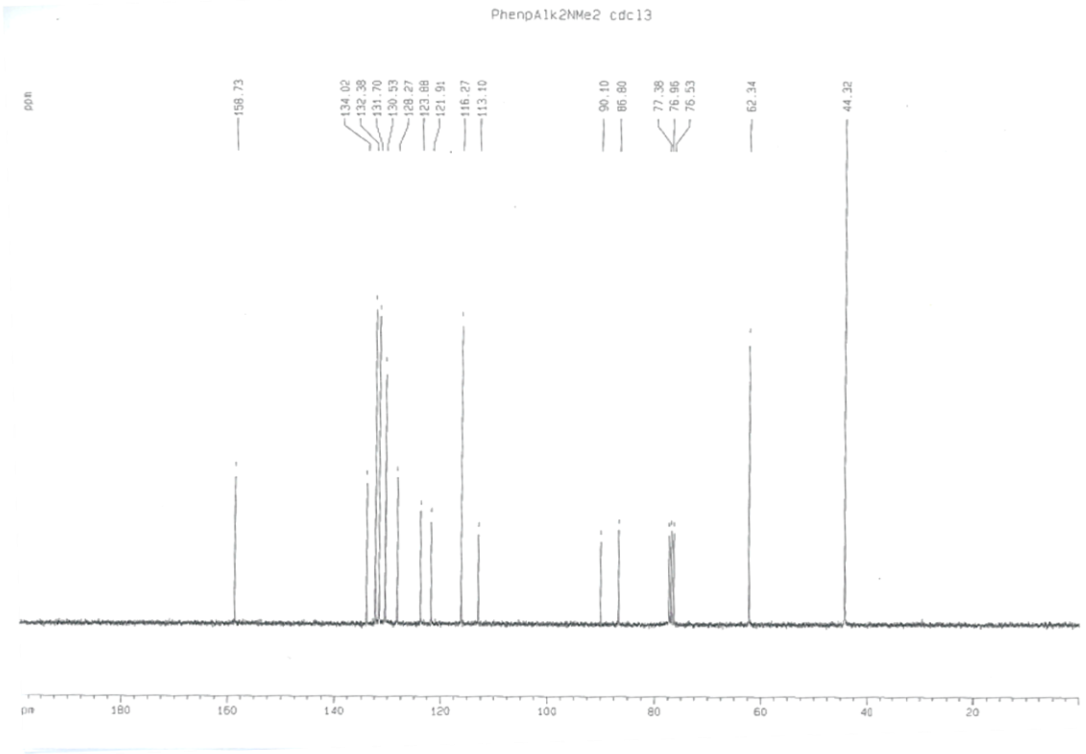

2. ^{1}H-NMR (300 MHz, CD_3OD)

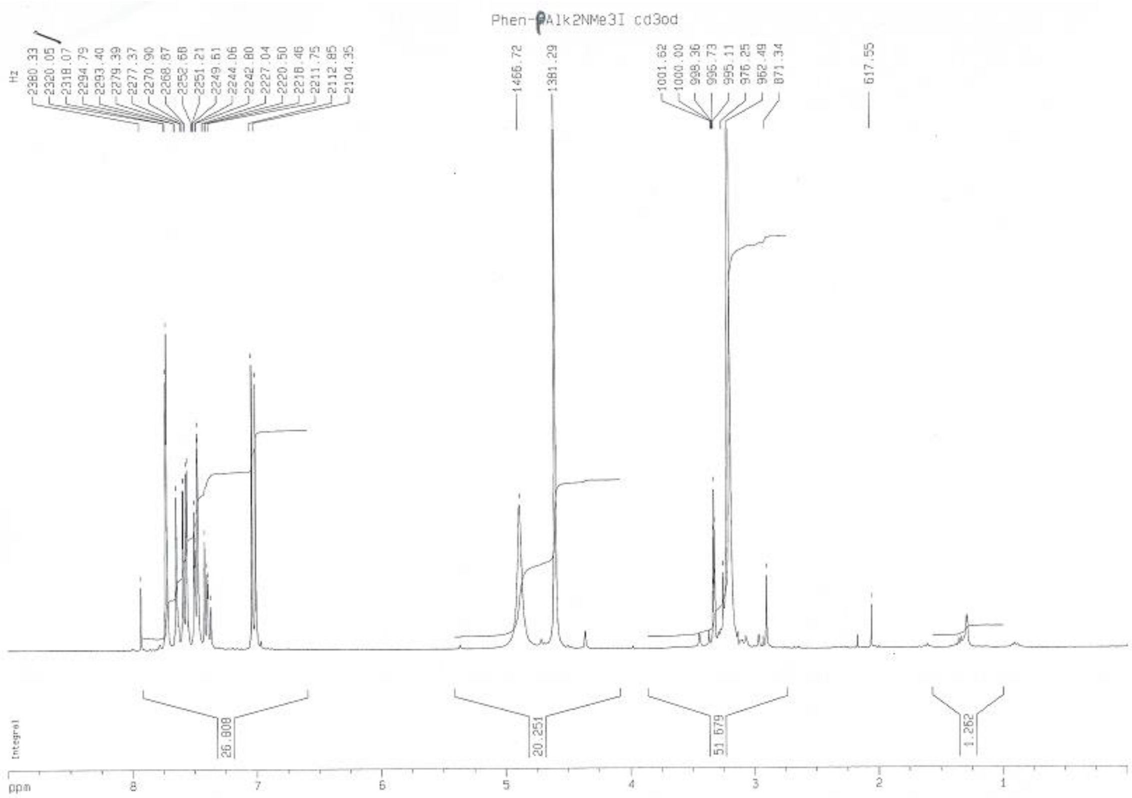

2. ^{13}C-NMR (75 MHz, CD_3OD)

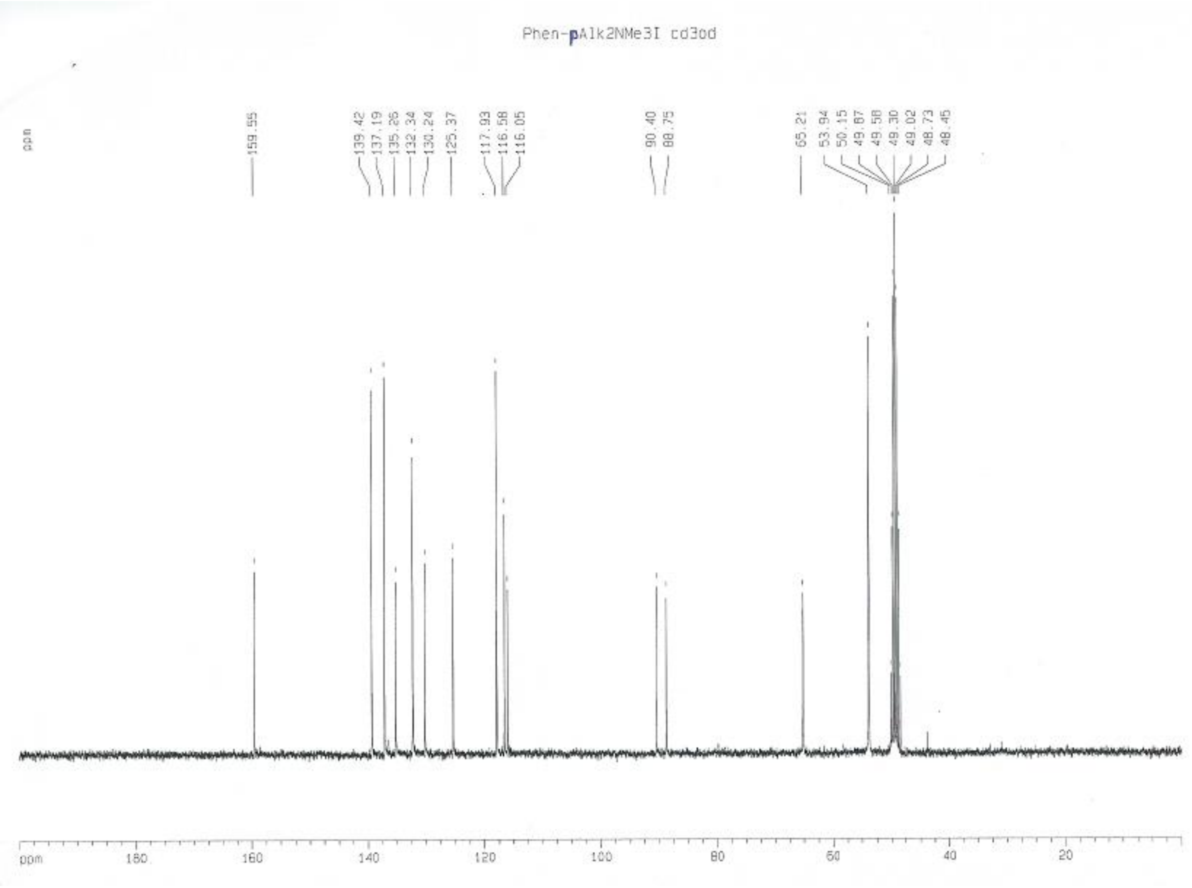

3. ^{1}H-NMR (300 MHz, $CDCl_3$)

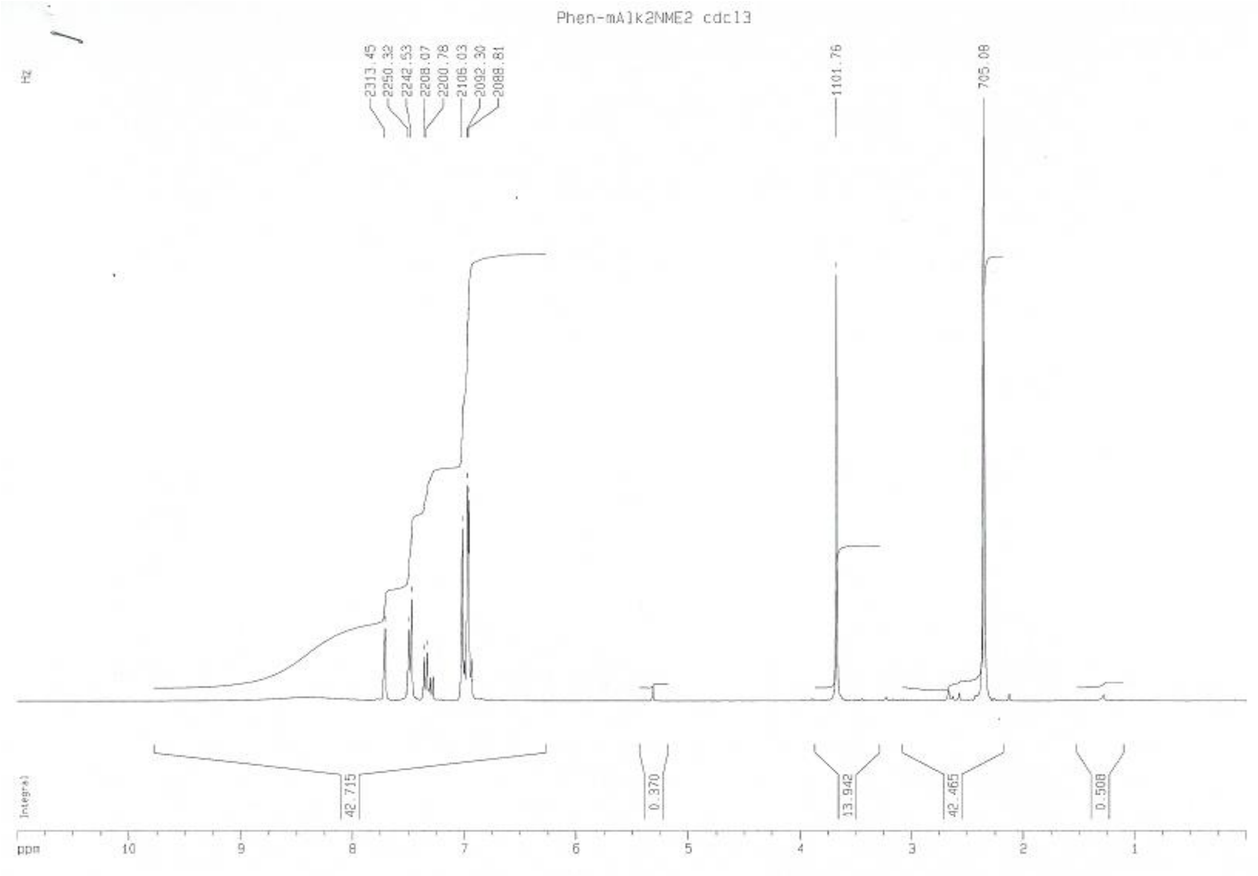

3. ^{13}C-NMR (75 MHz, $CDCl_3$)

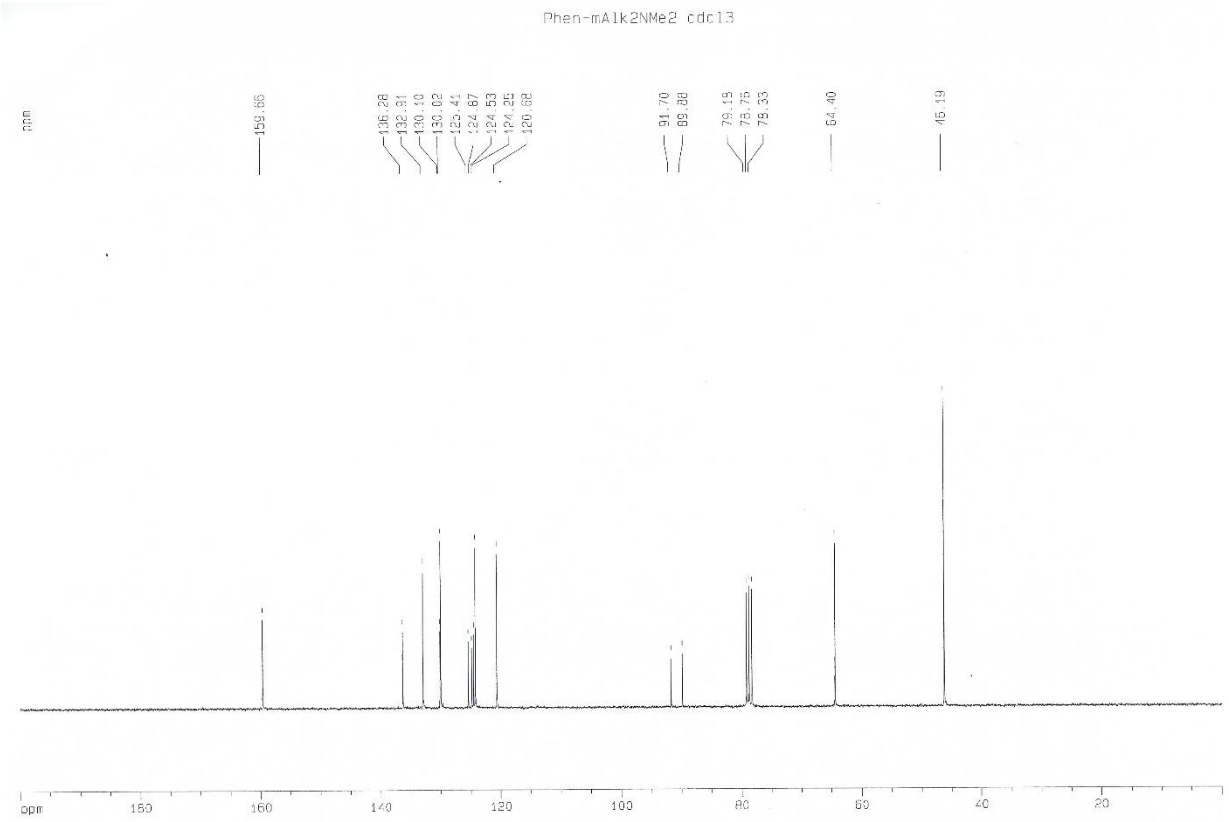

4. ^{1}H-NMR (300 MHz, CD_3OD)

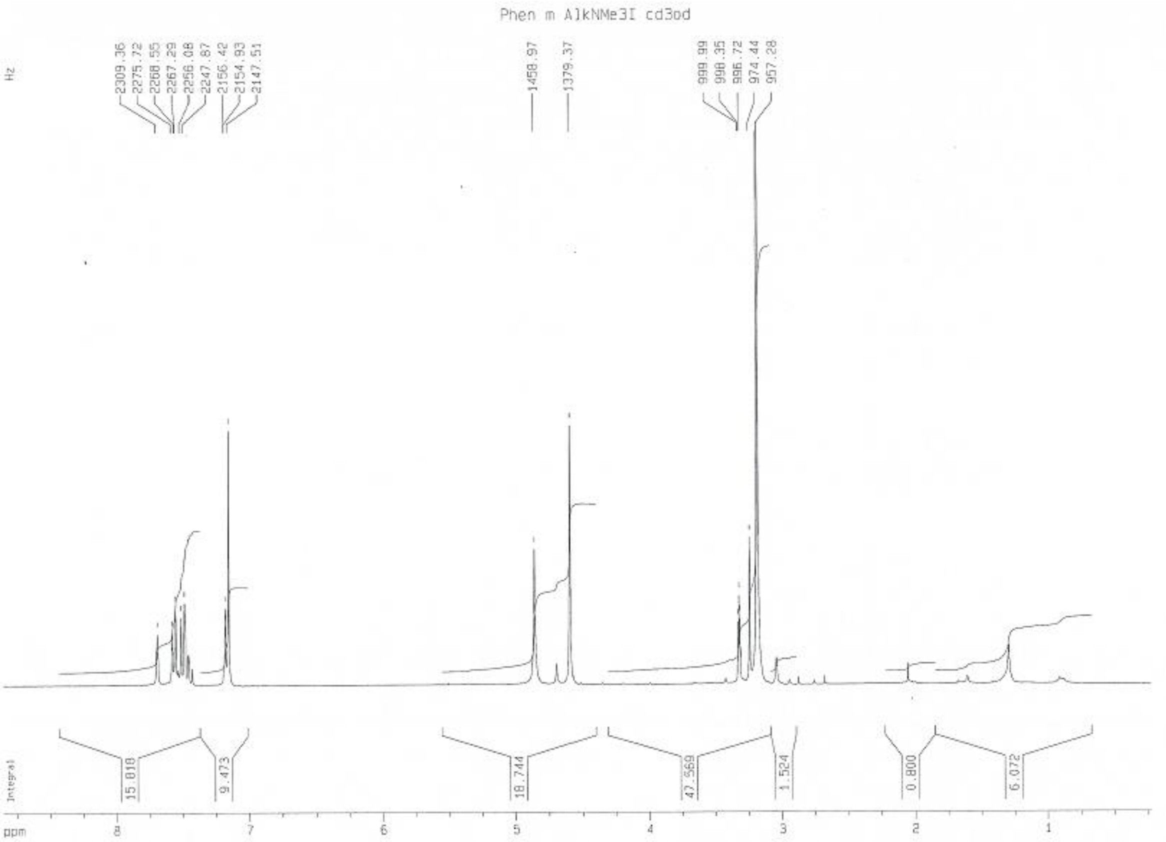

4. ^{13}C-NMR (75 MHz, CD_3OD)

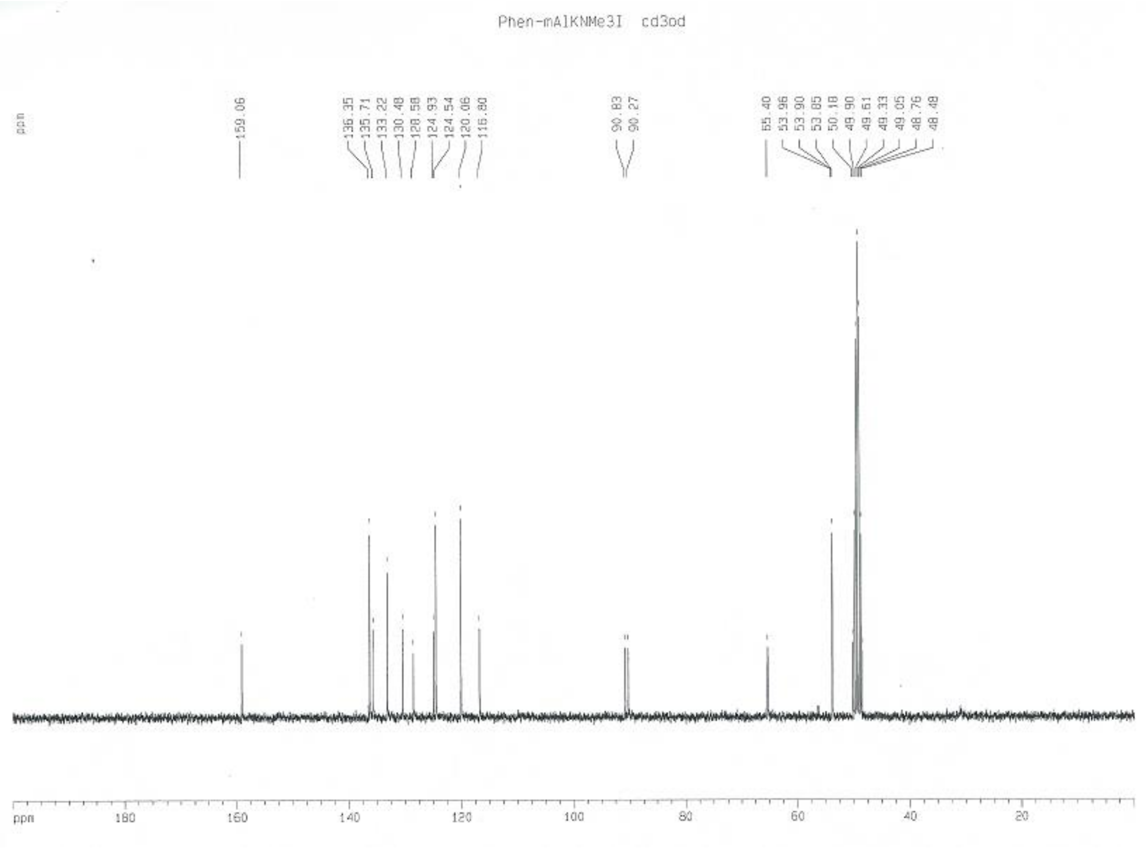

5. ^{1}H-NMR (300 MHz, DMSO-d_6)

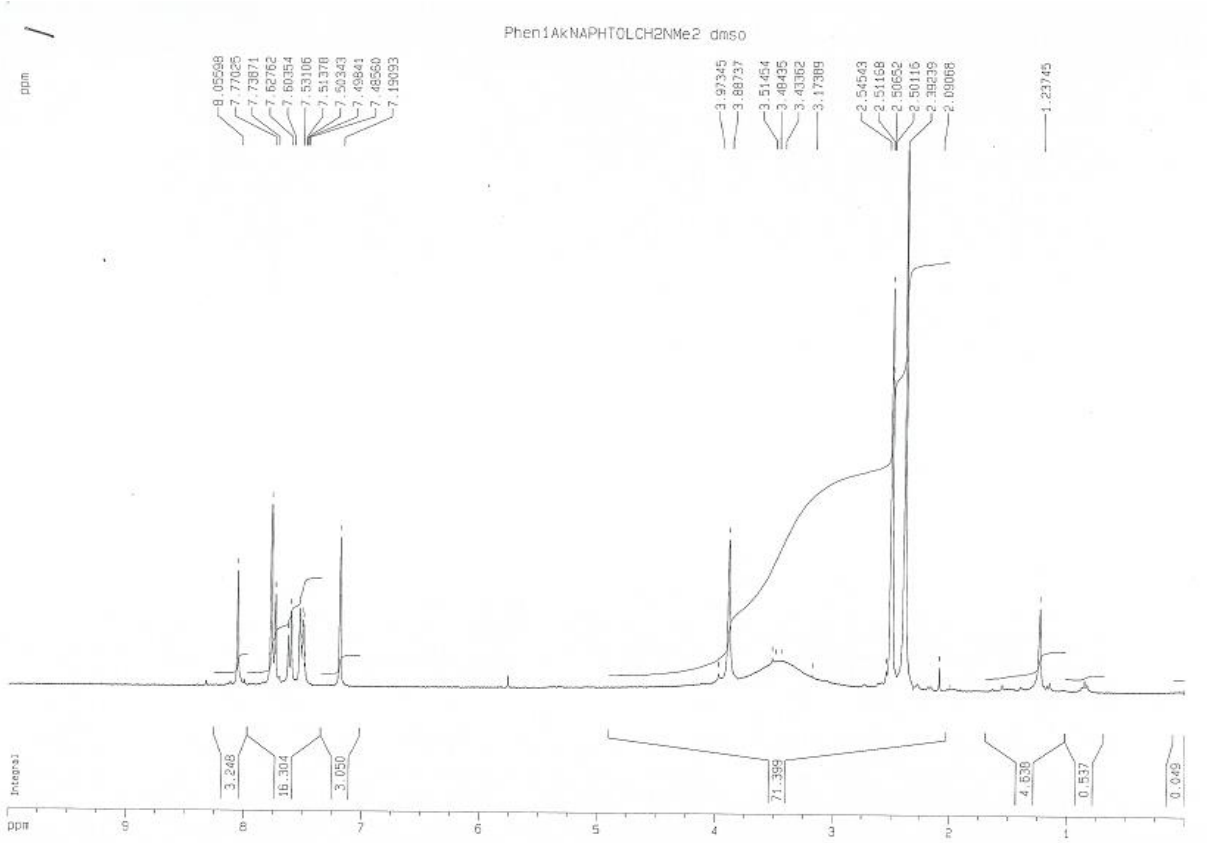

5. ^{13}C-NMR (75 MHz, DMSO-d_6)

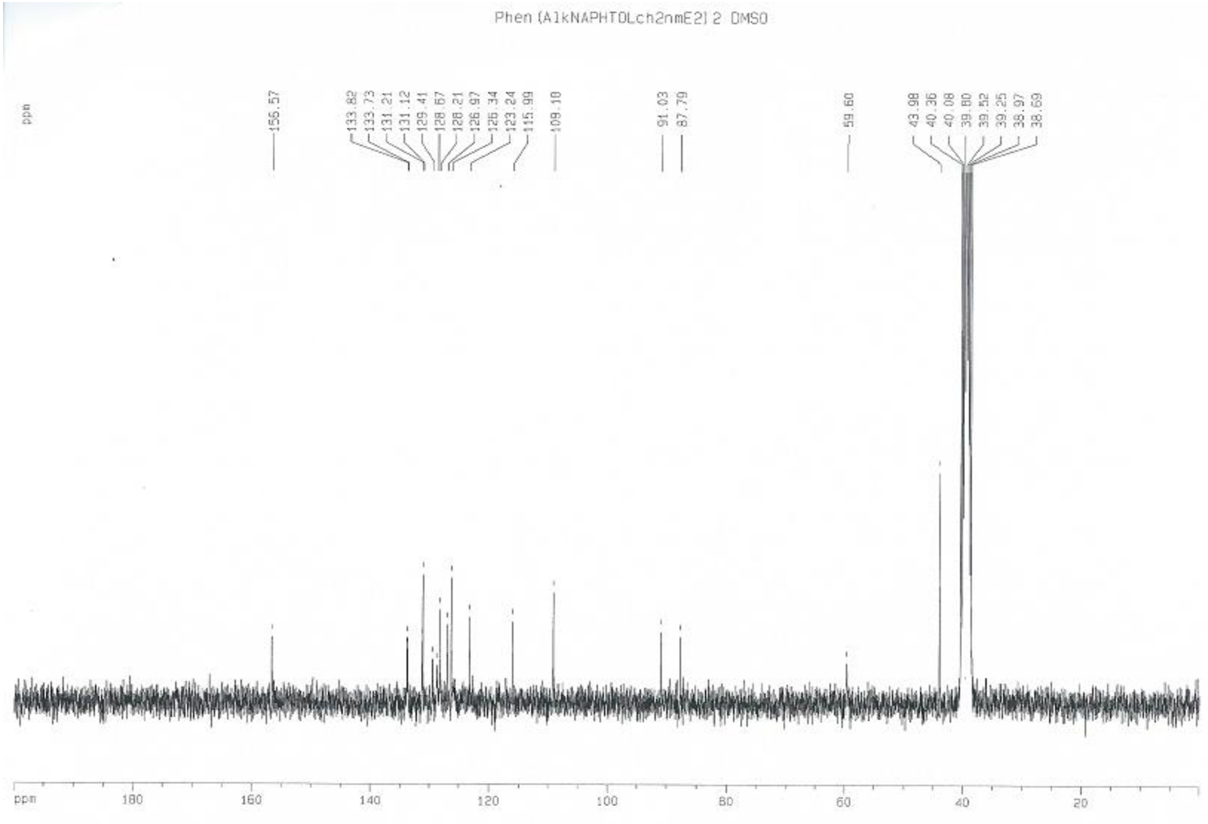

6. ^{1}H-NMR (300 MHz, CD_3OD)

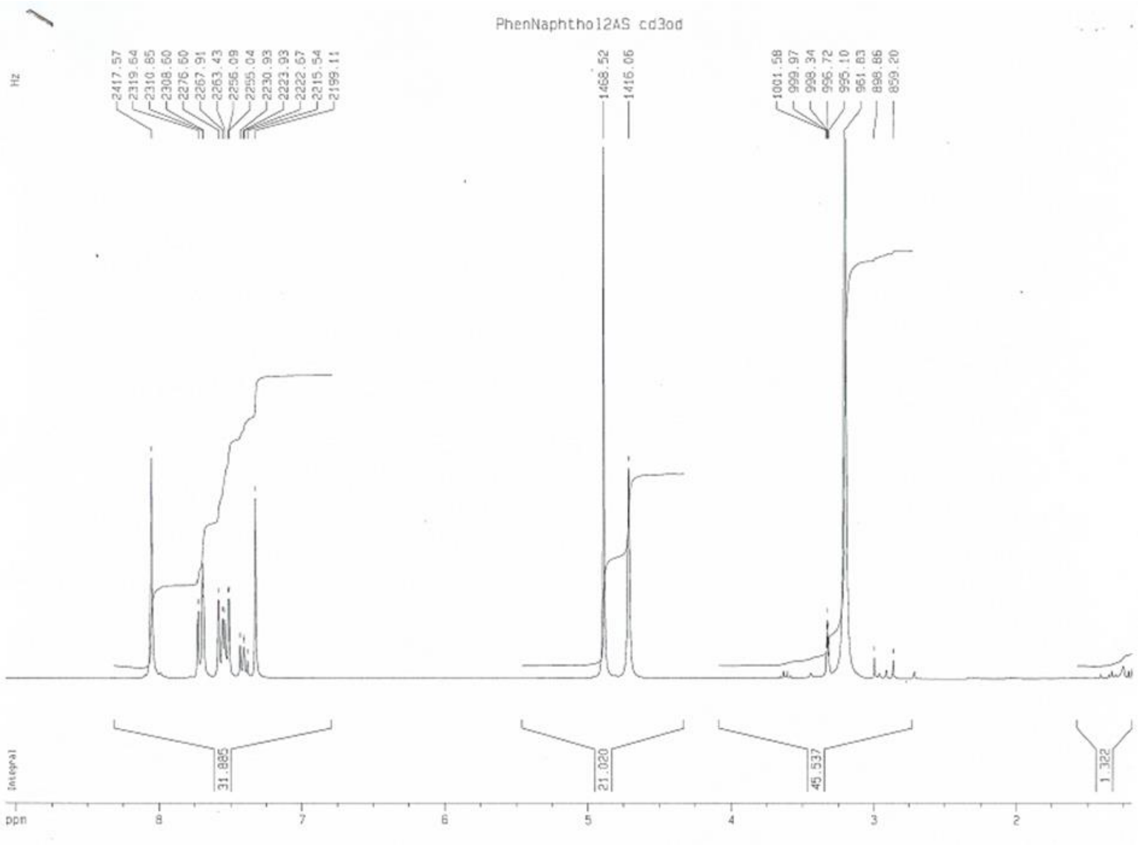

6. ^{13}C-NMR (75 MHz, CD_3OD)

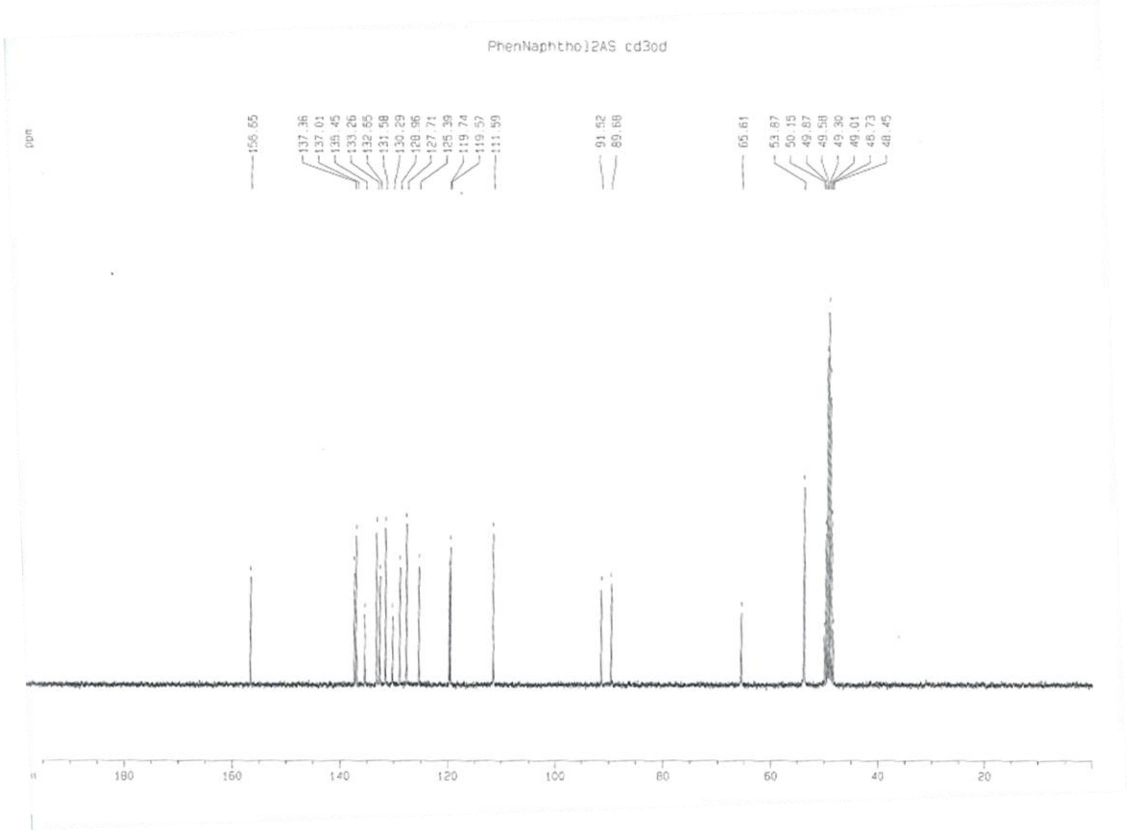

10. ^{1}H-NMR (300 MHz, DMSO-d$_6$)

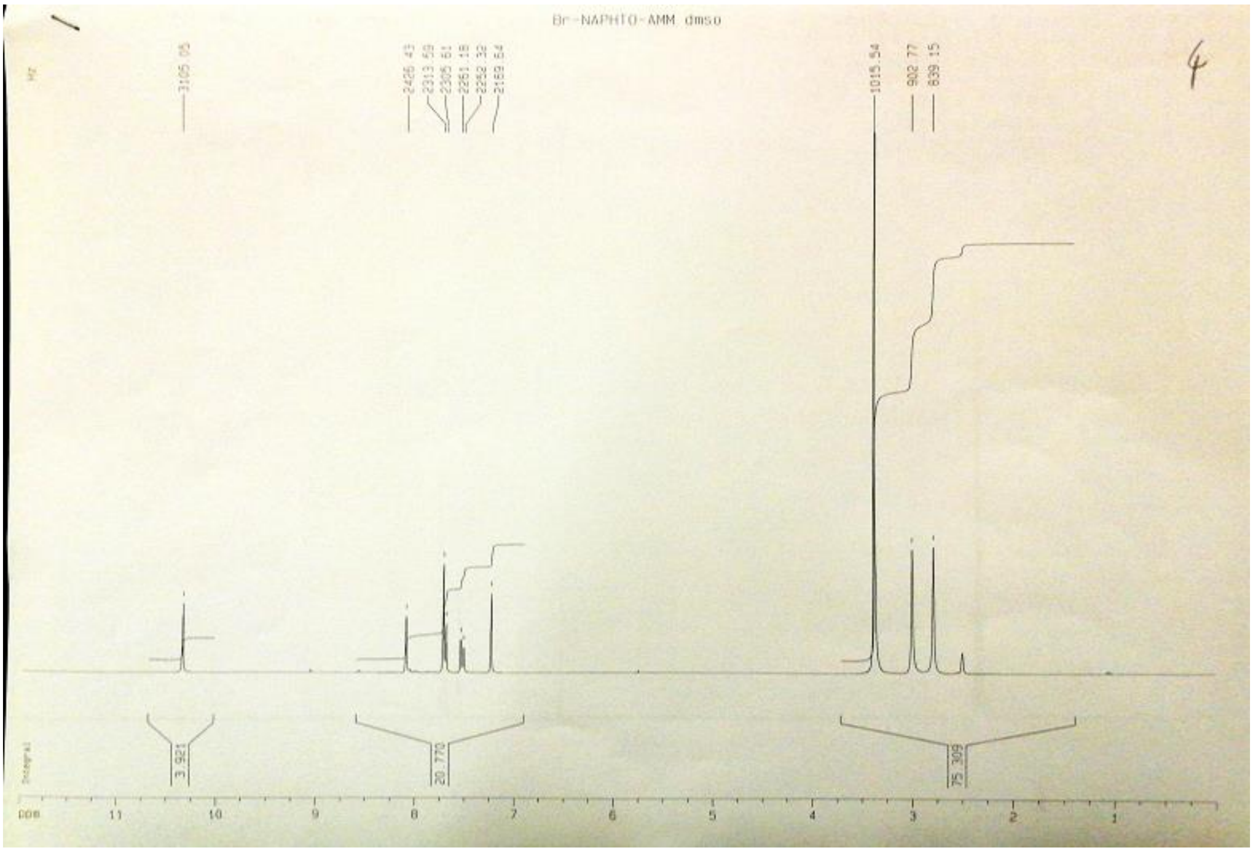

10. ^{13}C-NMR (75 MHz, DMSO-d$_6$)

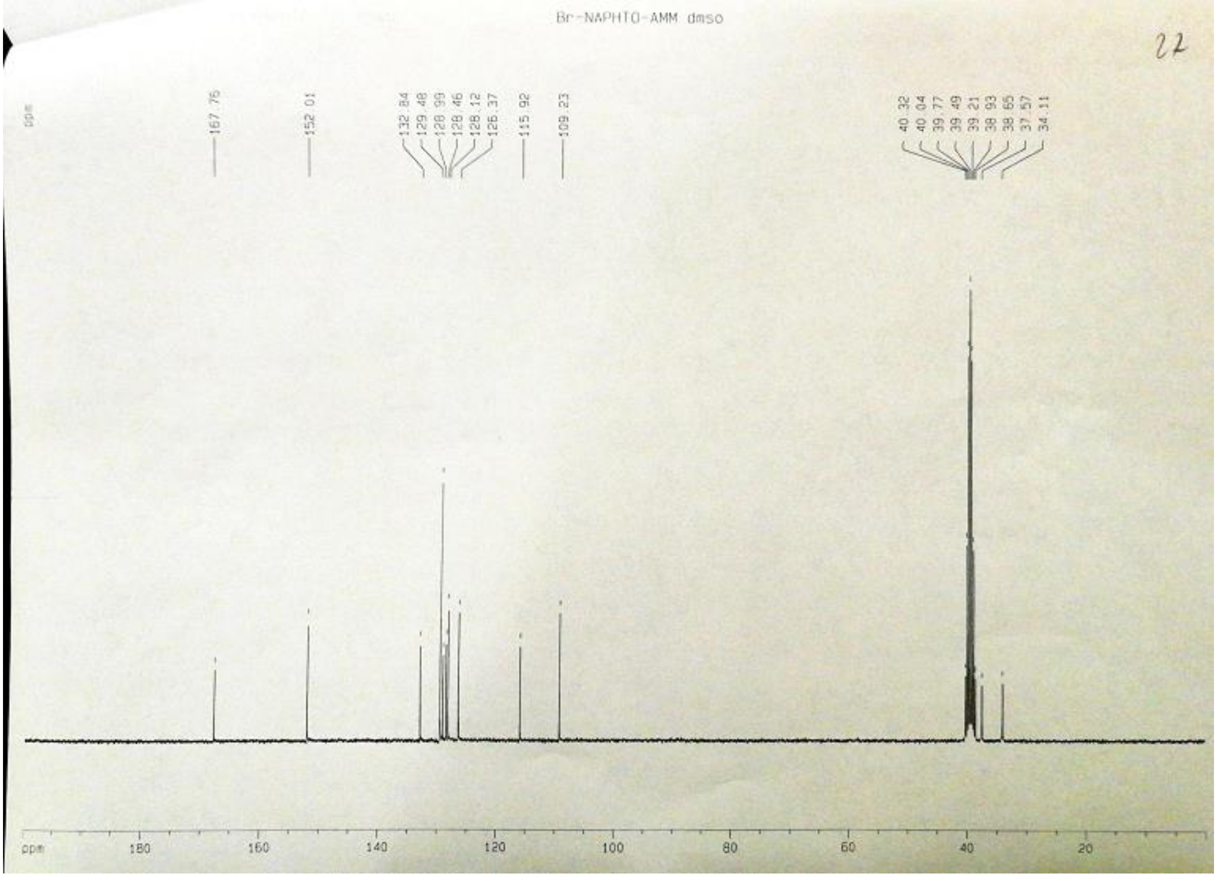

11. ^{1}H-NMR (300 MHz, DMSO-d$_6$)

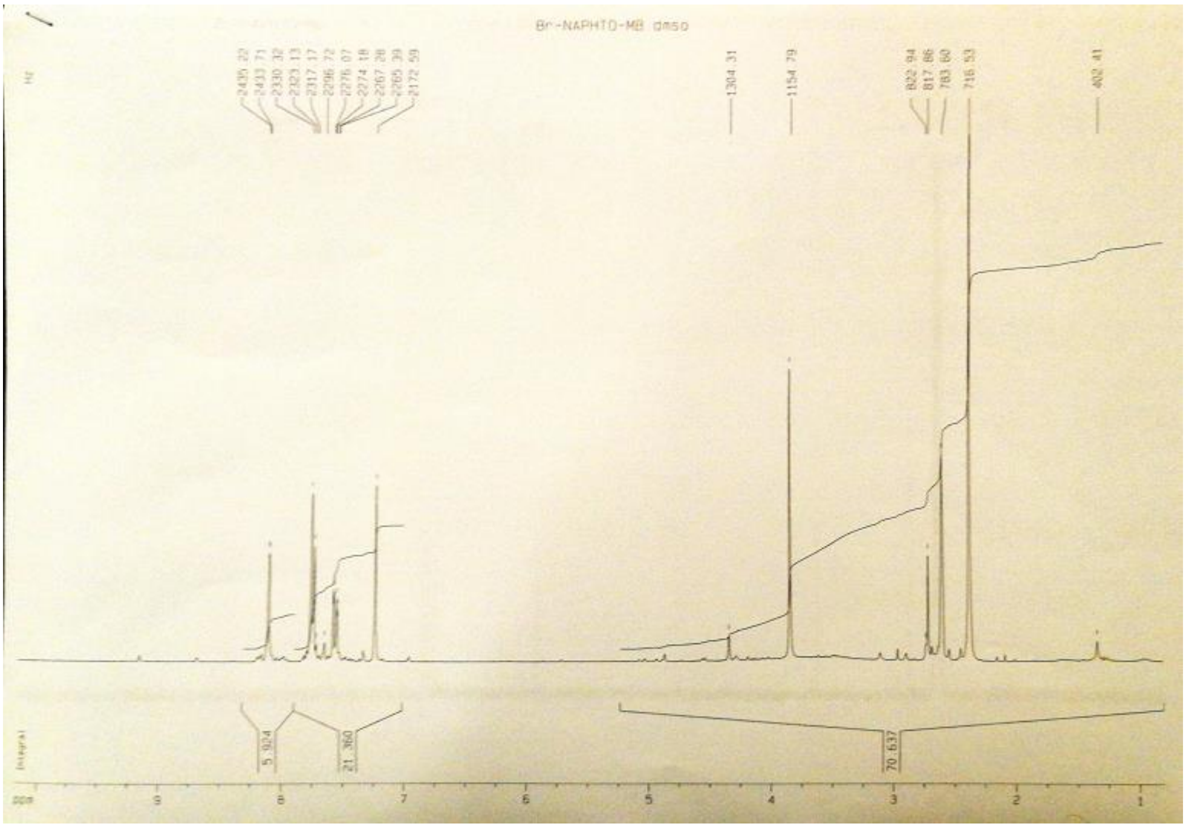

11. ^{13}C-NMR (75 MHz, DMSO-d$_6$)

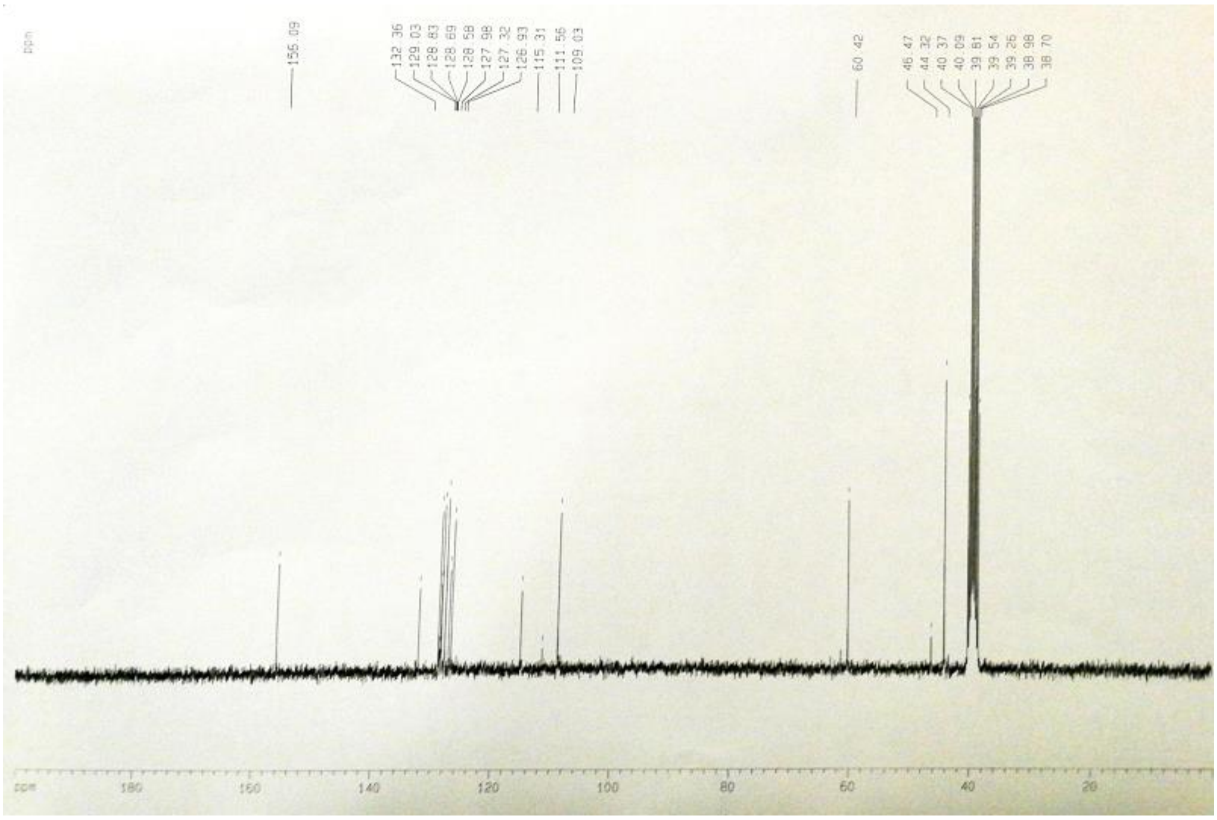

12. ^{1}H-NMR (300 MHz, $CDCl_3$)

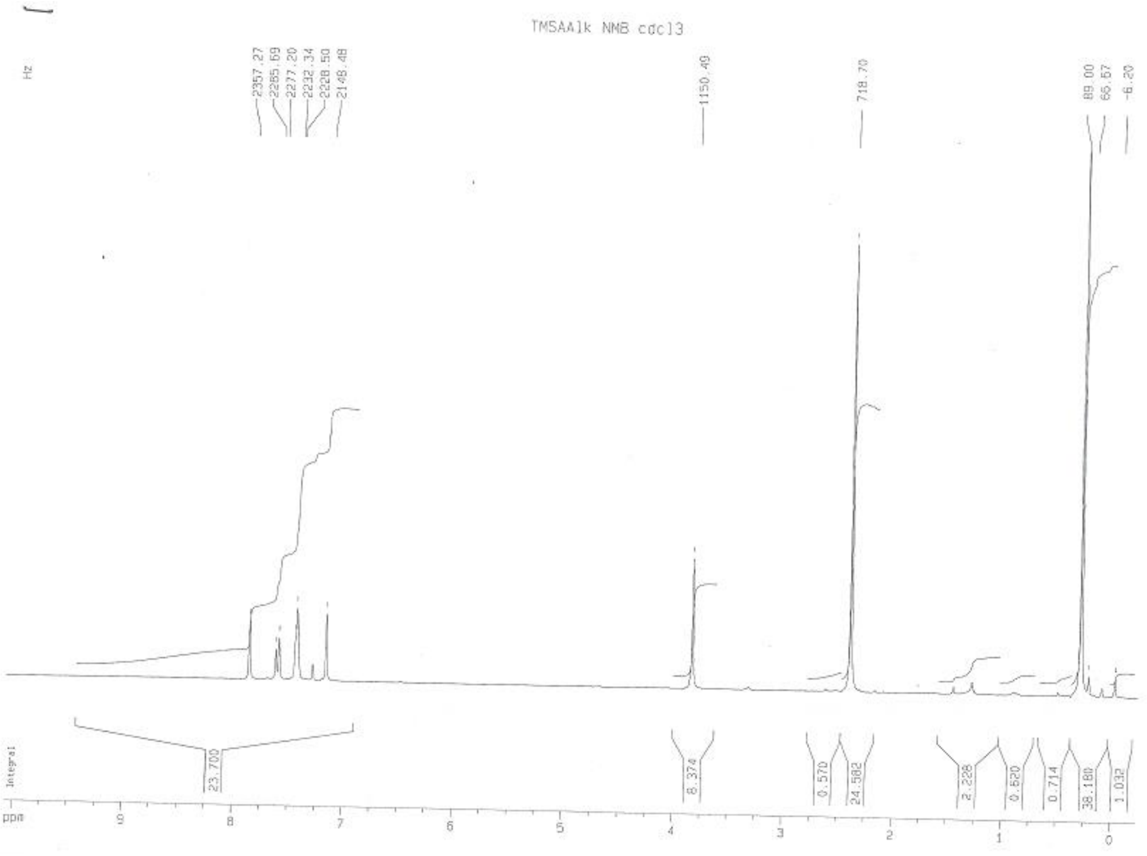

12. ^{13}C-NMR (300 MHz, $CDCl_3$)

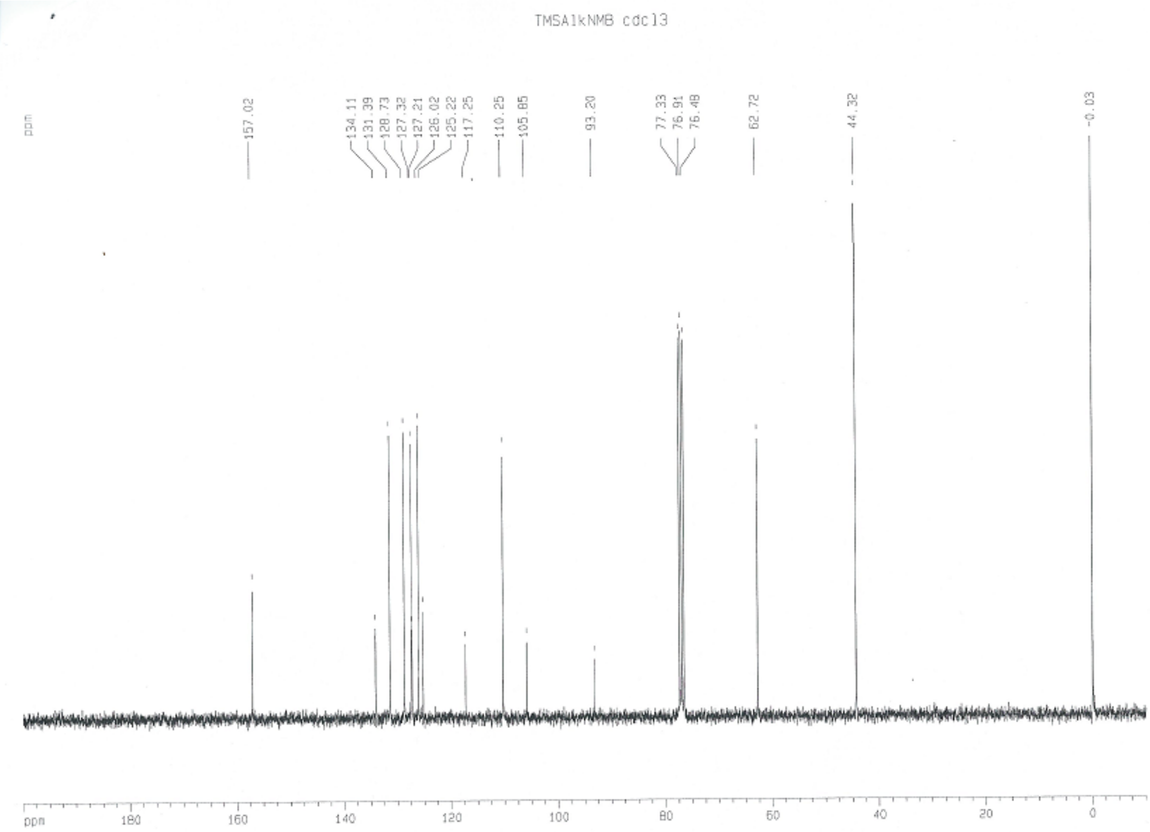

13. ^{1}H-NMR (300 MHz, $CDCl_3$)

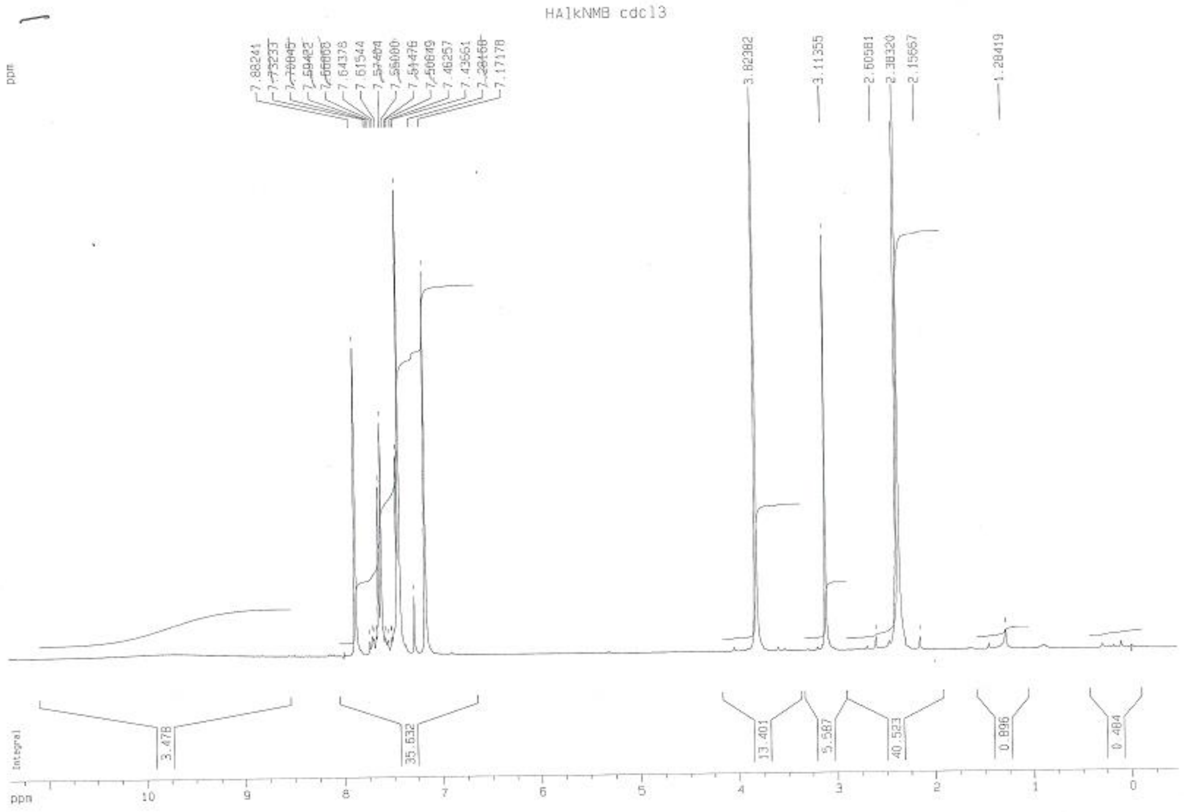

13. ^{13}C-NMR (300 MHz, $CDCl_3$)

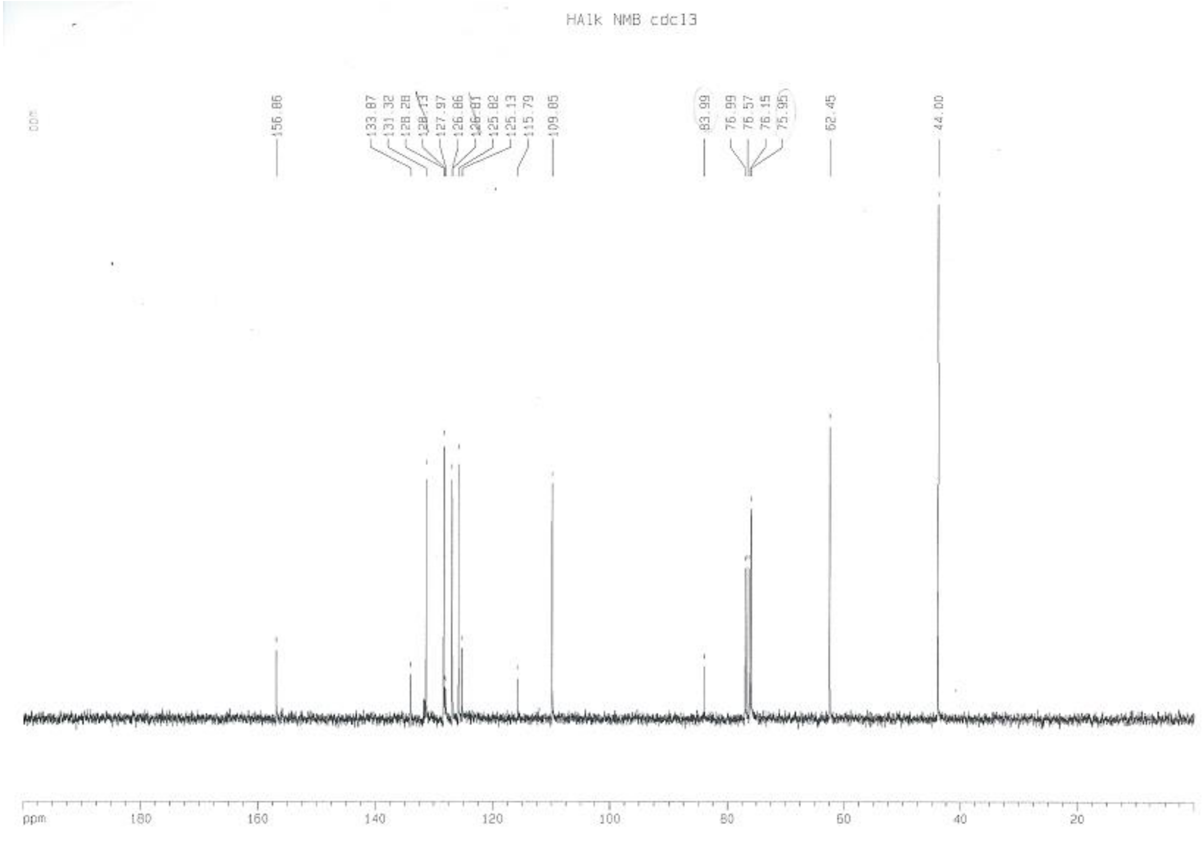

16. ^{1}H-NMR (300 MHz, DMSO-d$_6$)

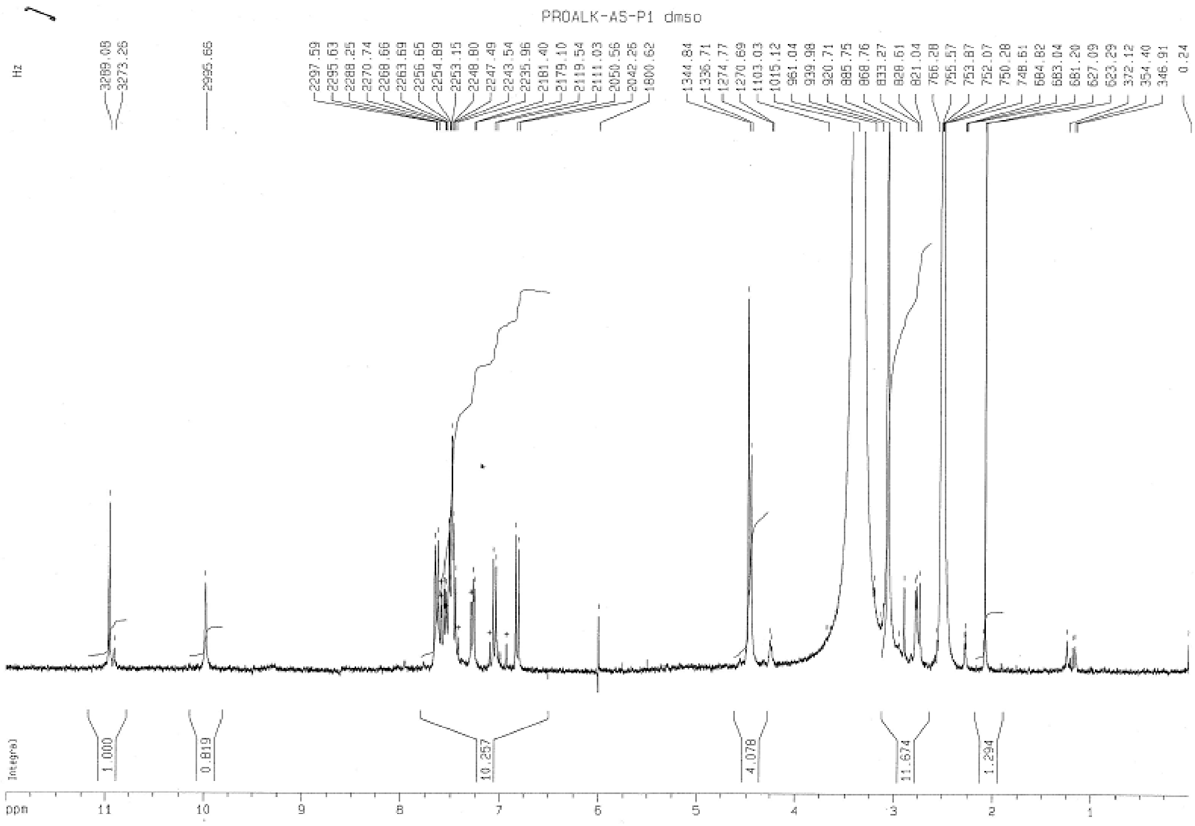

16. ^{13}C-NMR (300 MHz, DMSO-d$_6$)

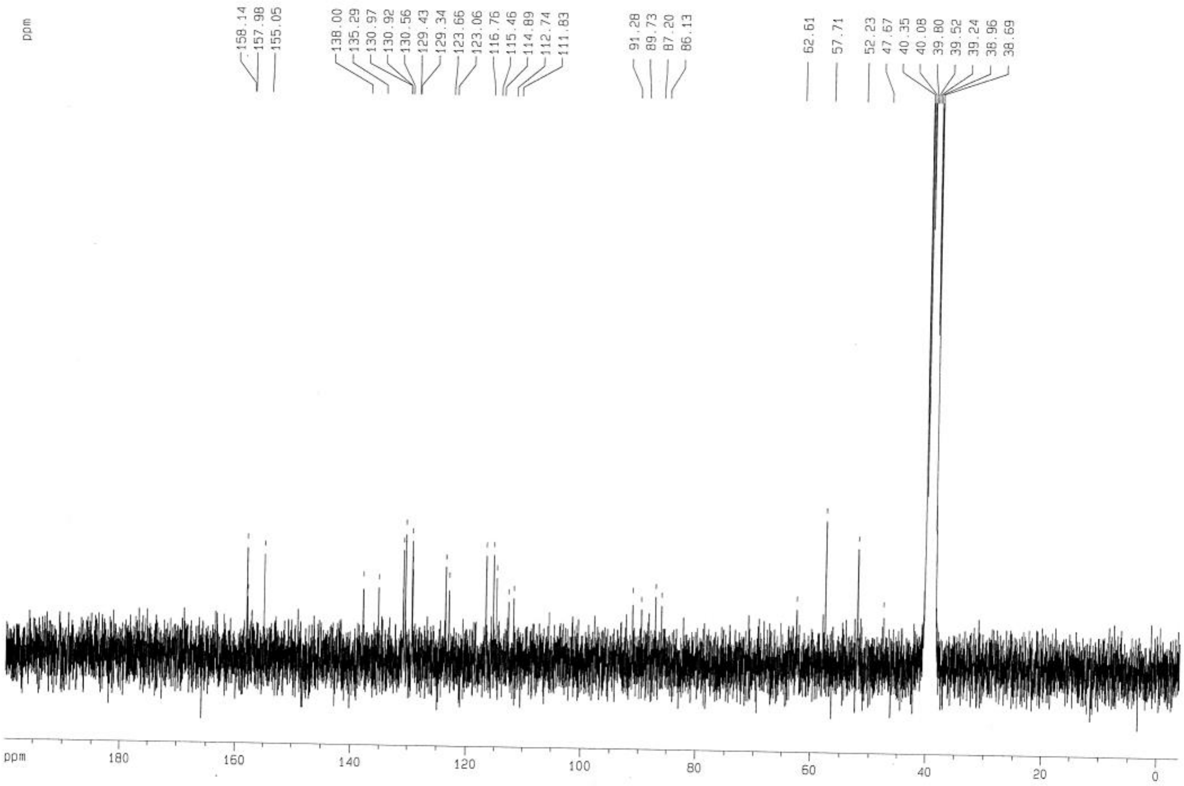

17. ^{1}H-NMR (300 MHz, DMSO-d_6)

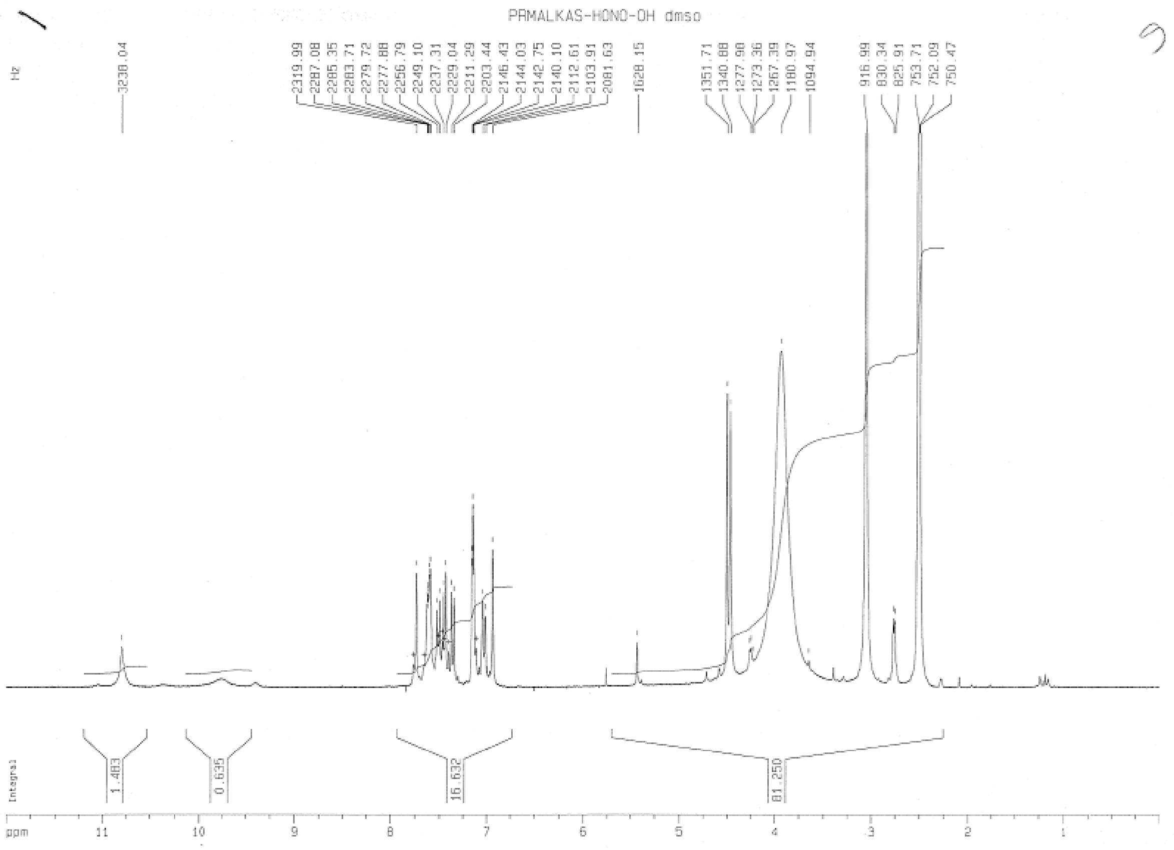

17. ^{13}C-NMR (75 MHz, DMSO-d_6)

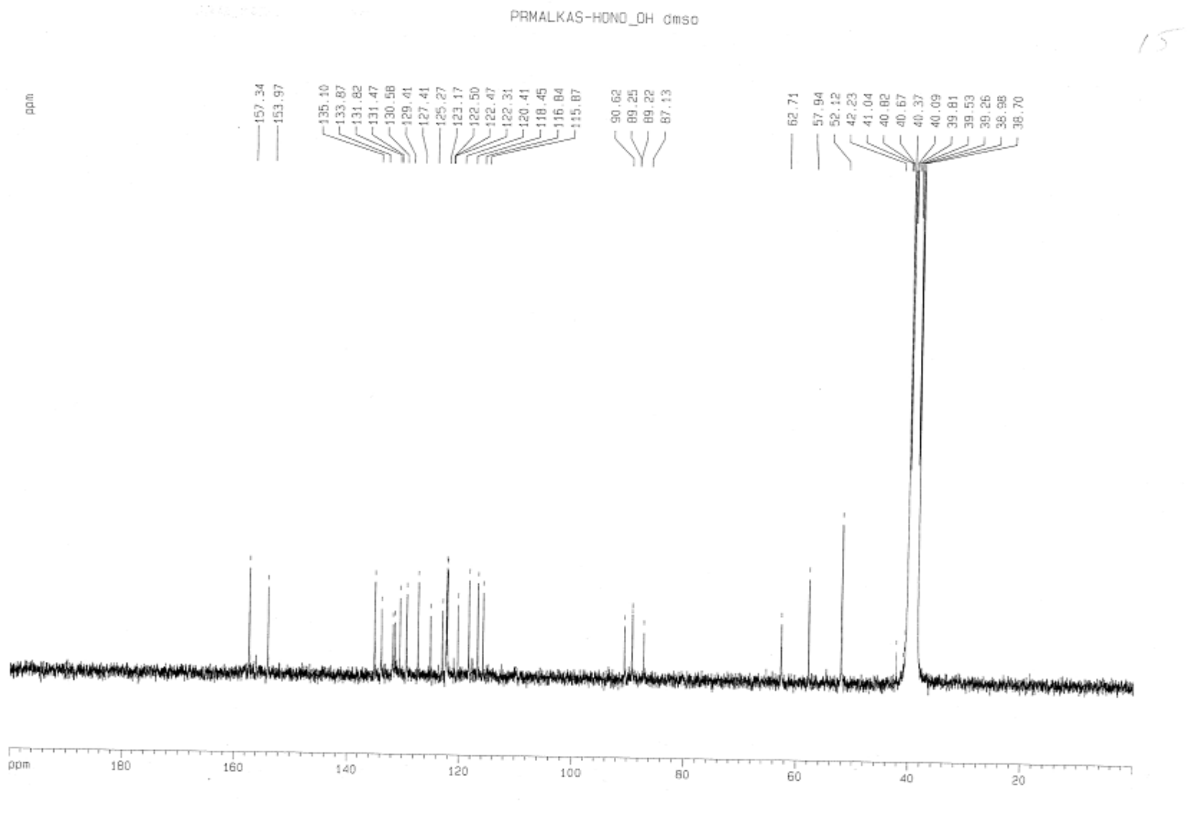

19. ^{1}H-NMR (300 MHz, DMSO-d$_6$)

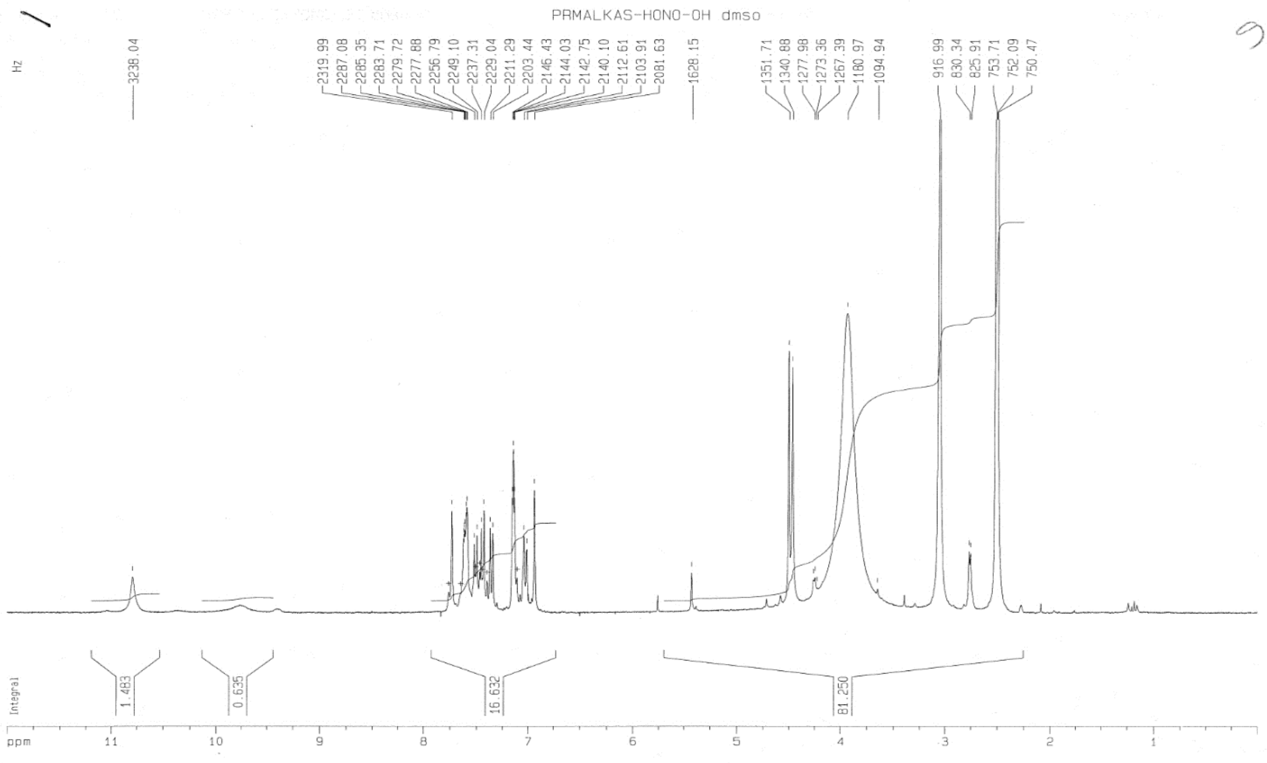

19. ^{13}C-NMR (75 MHz, DMSO-d$_6$)

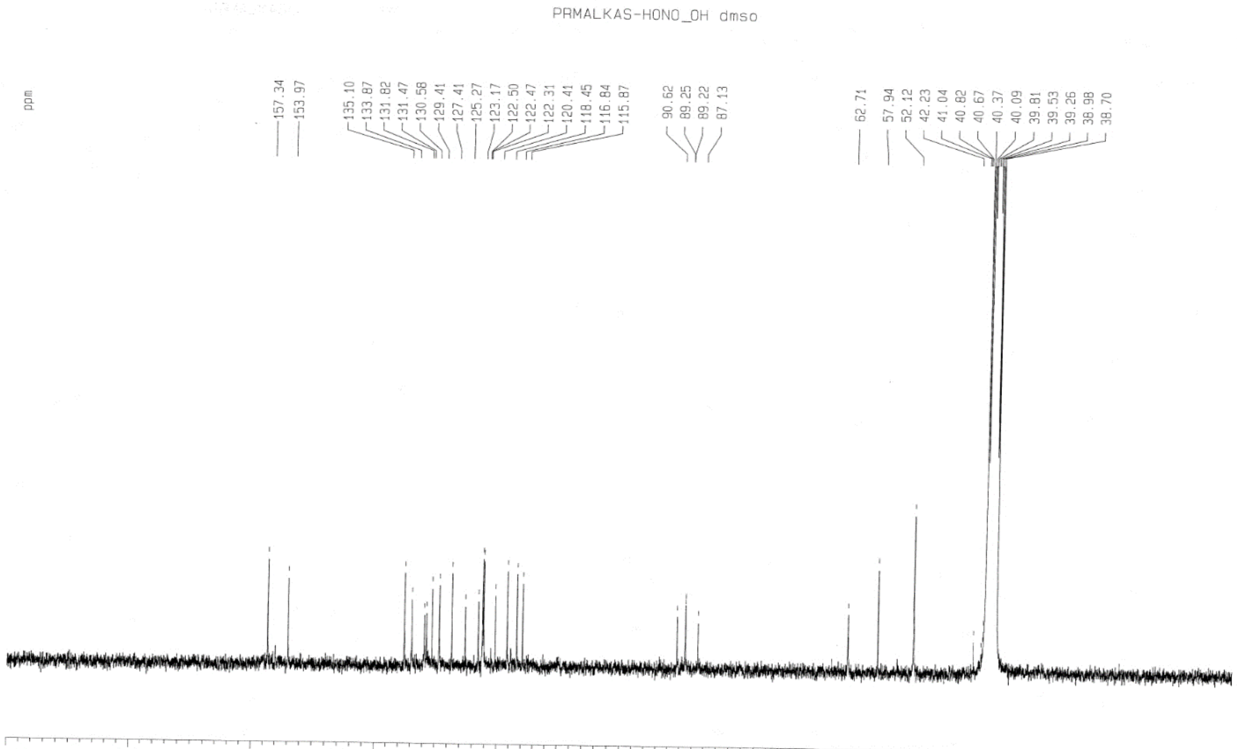

20. ^{1}H-NMR (300 MHz, DMSO-d$_6$)

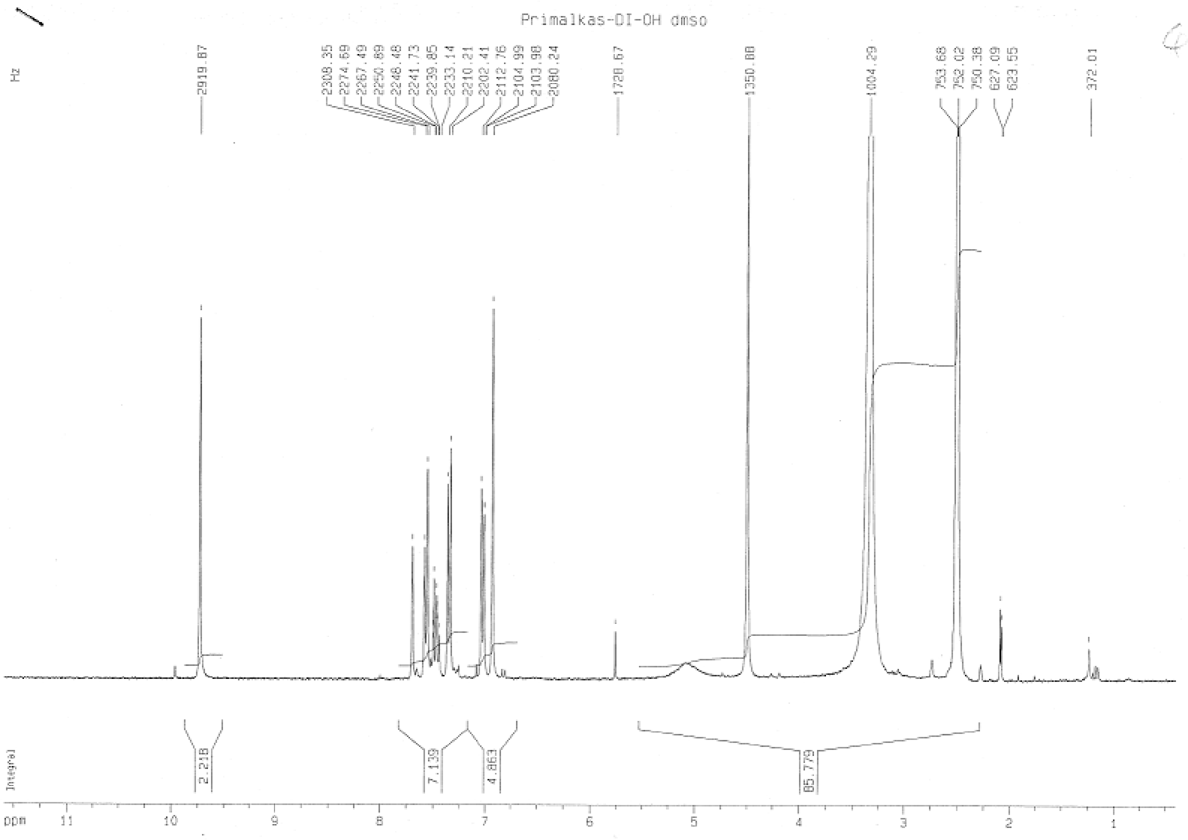

20. ^{13}C-NMR (75 MHz, DMSO-d$_6$)

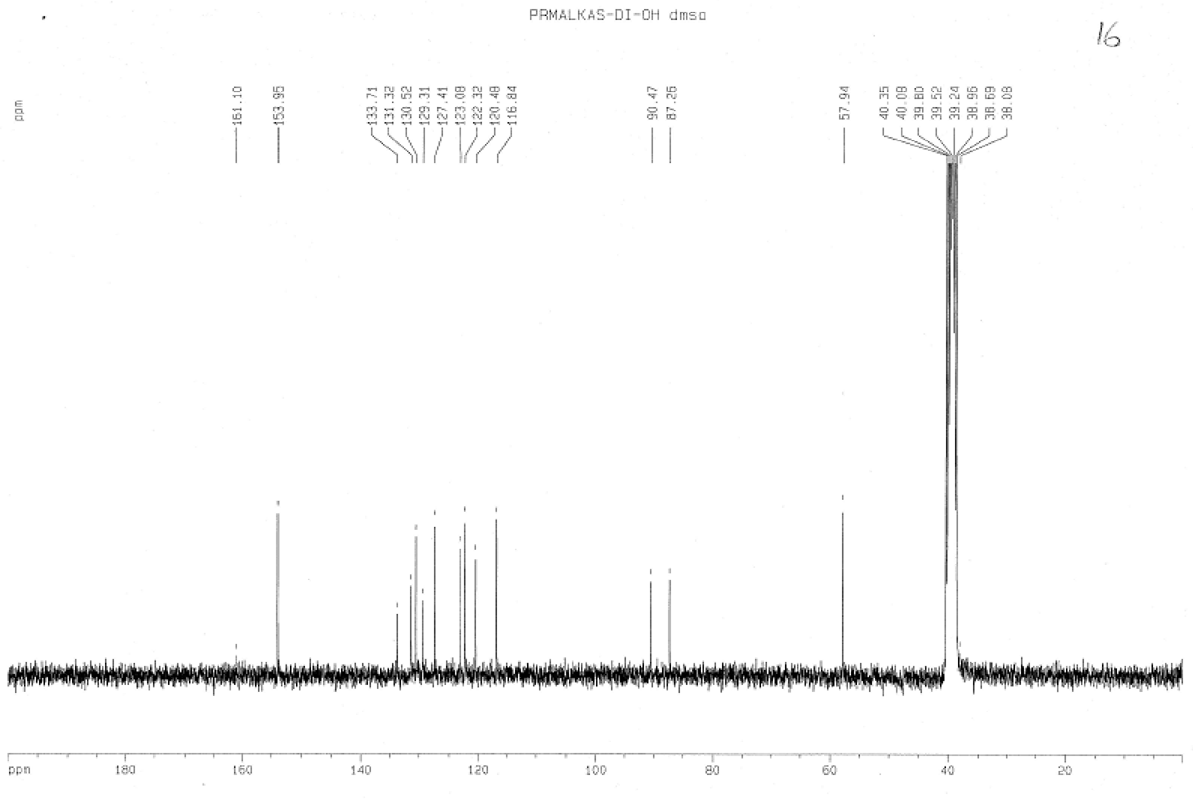

21. ^{1}H-NMR (300 MHz, DMSO-d_6)

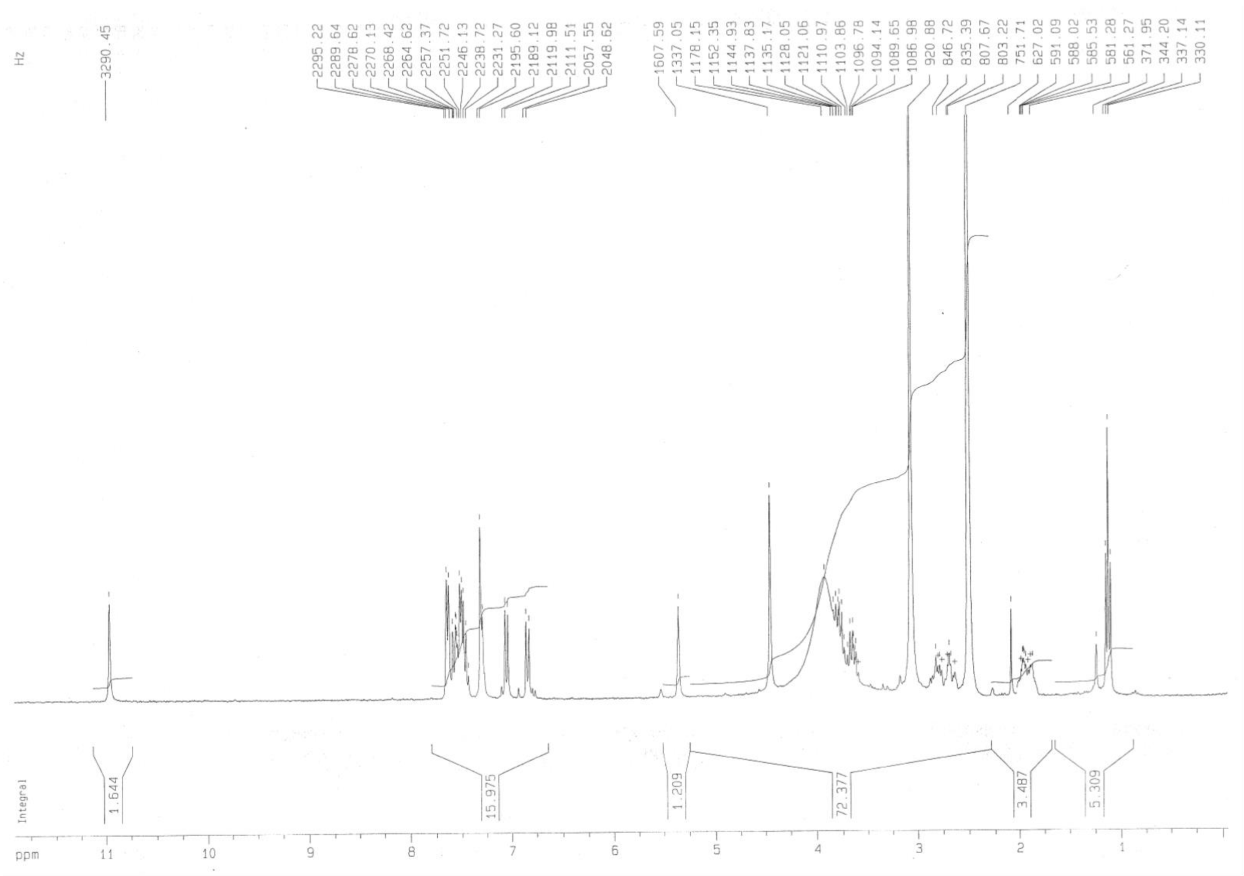

21. ^{13}C-NMR (75 MHz, DMSO-d_6)

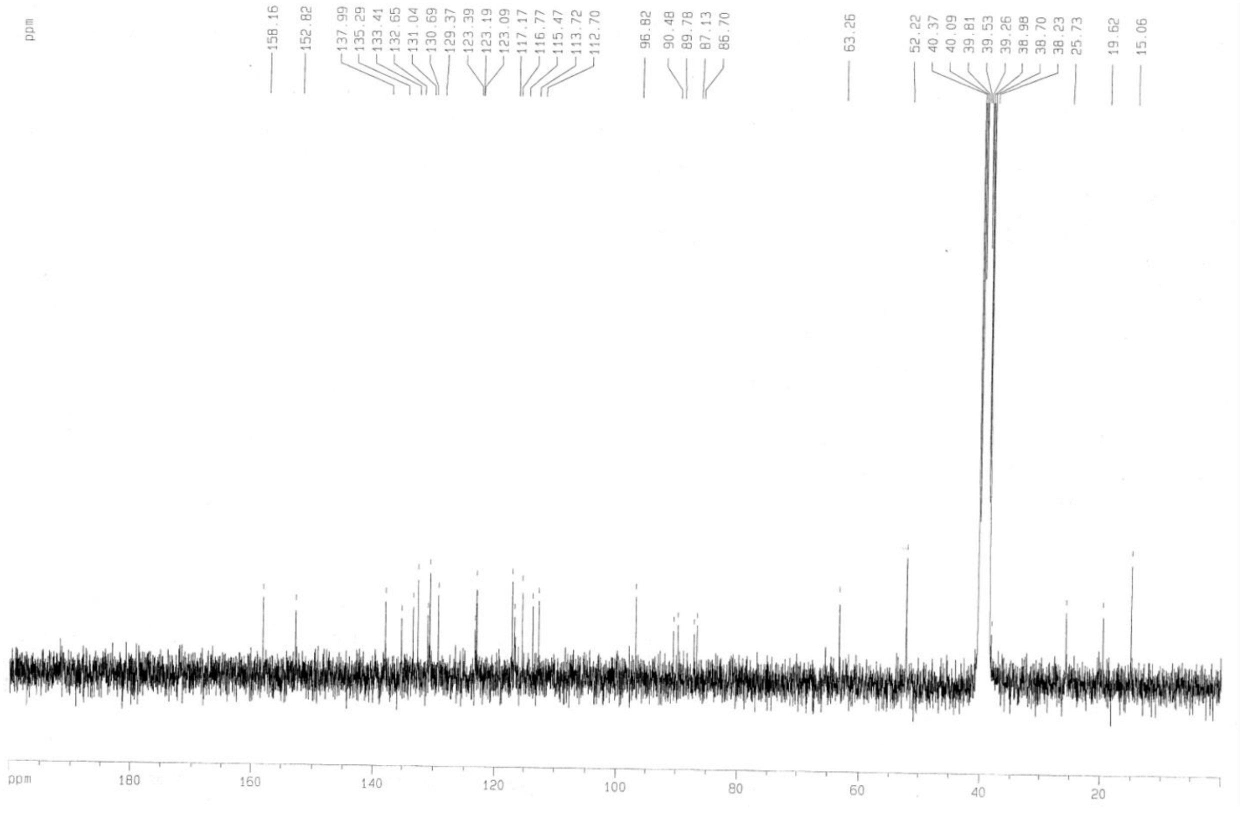

ESI-MS Data

15 (ESI-MS, Positive mode): mass calcd. for **15** $[M+H]^+$ requires m/z = 398.48, found m/z = 398.19.

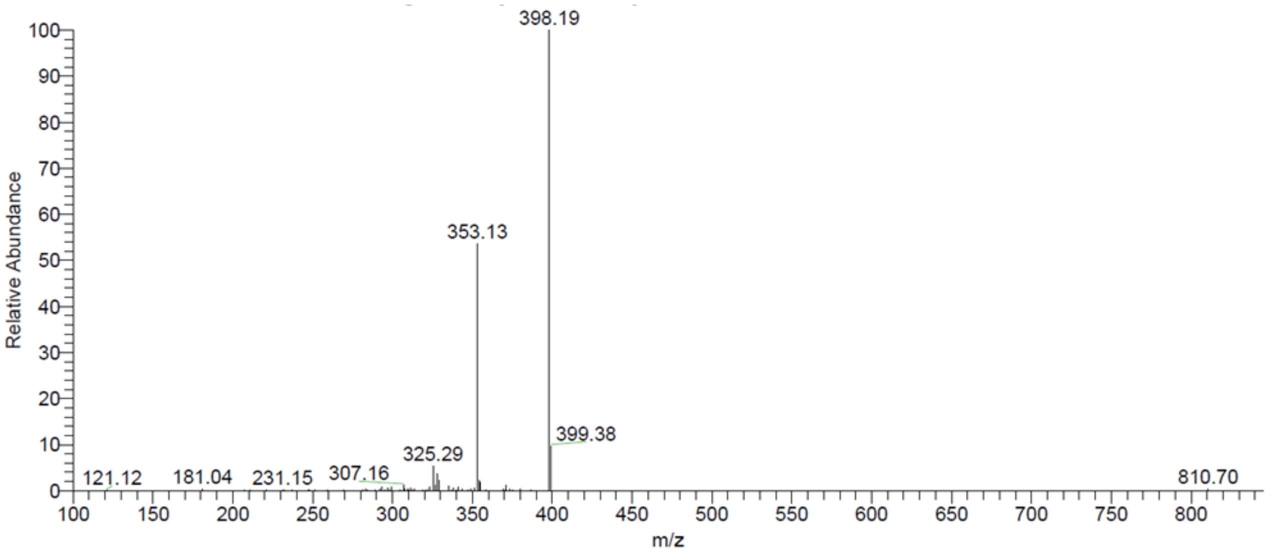

16 (ESI-MS, Positive mode): mass calcd. for **16** $[M+H]^+$ requires m/z = 412.51, found m/z = 412.25.

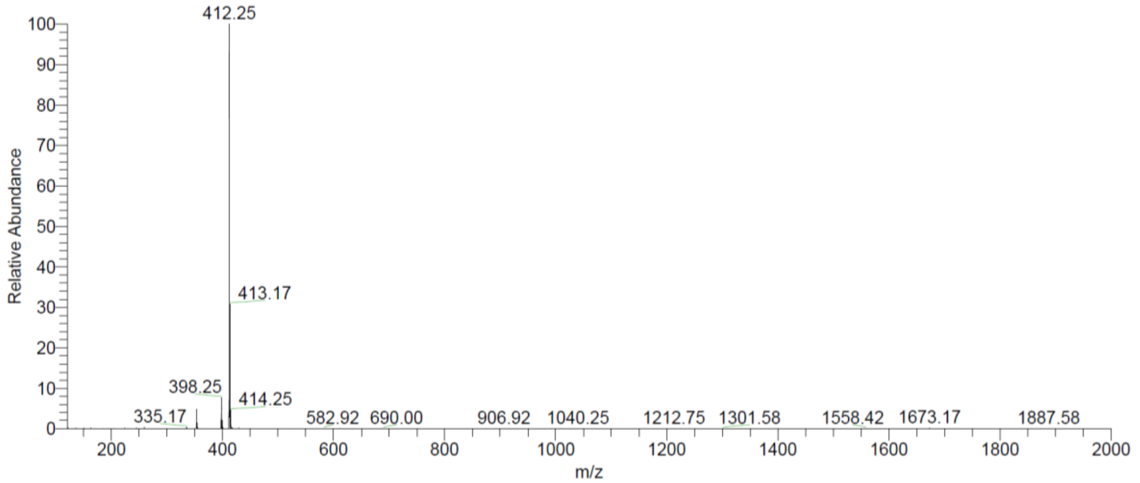

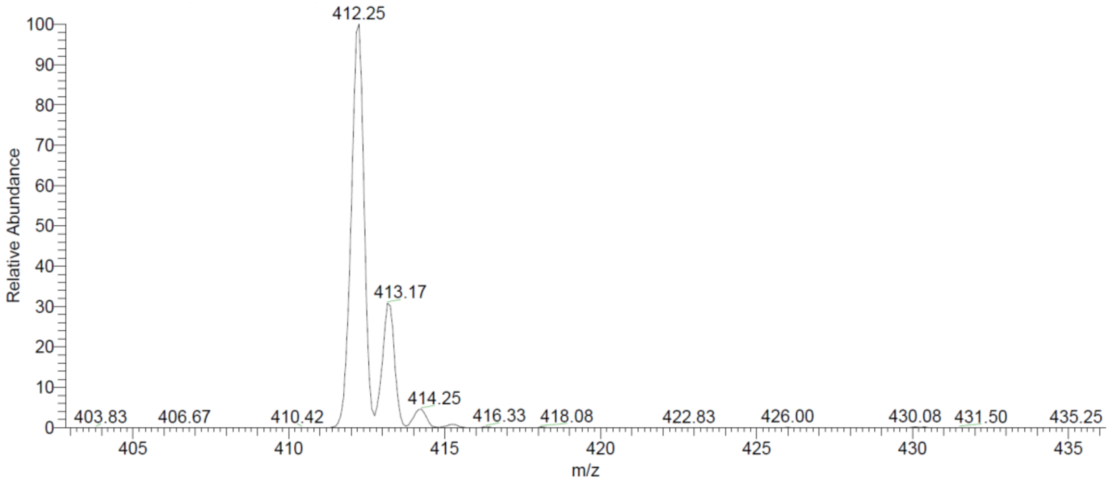

17 (ESI-MS, Negative mode): mass calcd. for **17** $[M-H]^-$ requires m/z = 369.4, found m/z = 369.18.

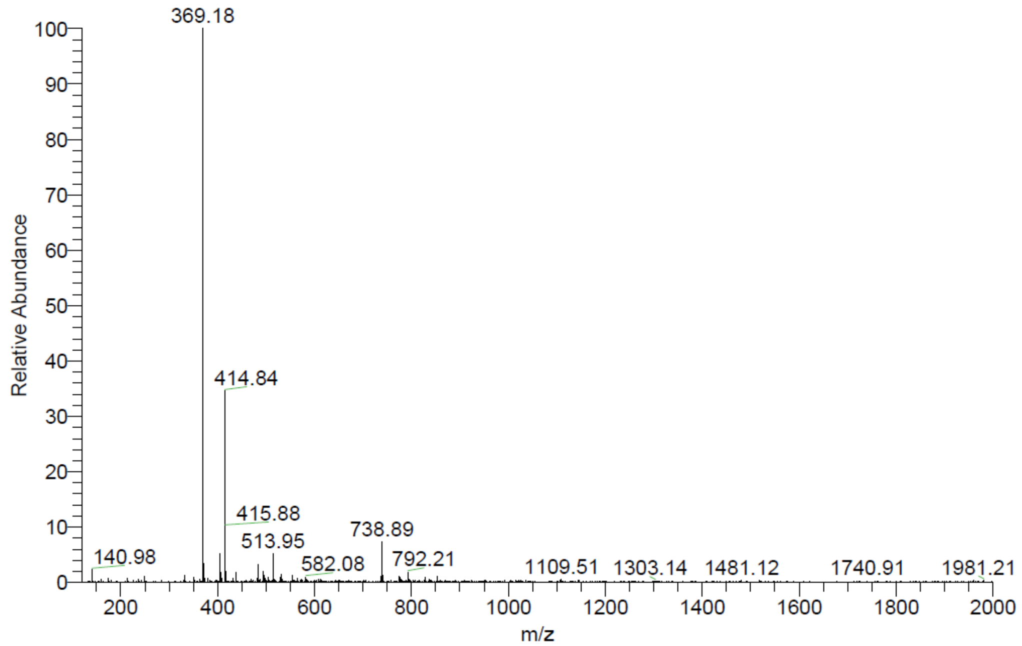

18. (ESI-MS, Positive mode): mass calcd. for **18** $[M+H]^+$ requires m/z = 398.48, found m/z = 398.19

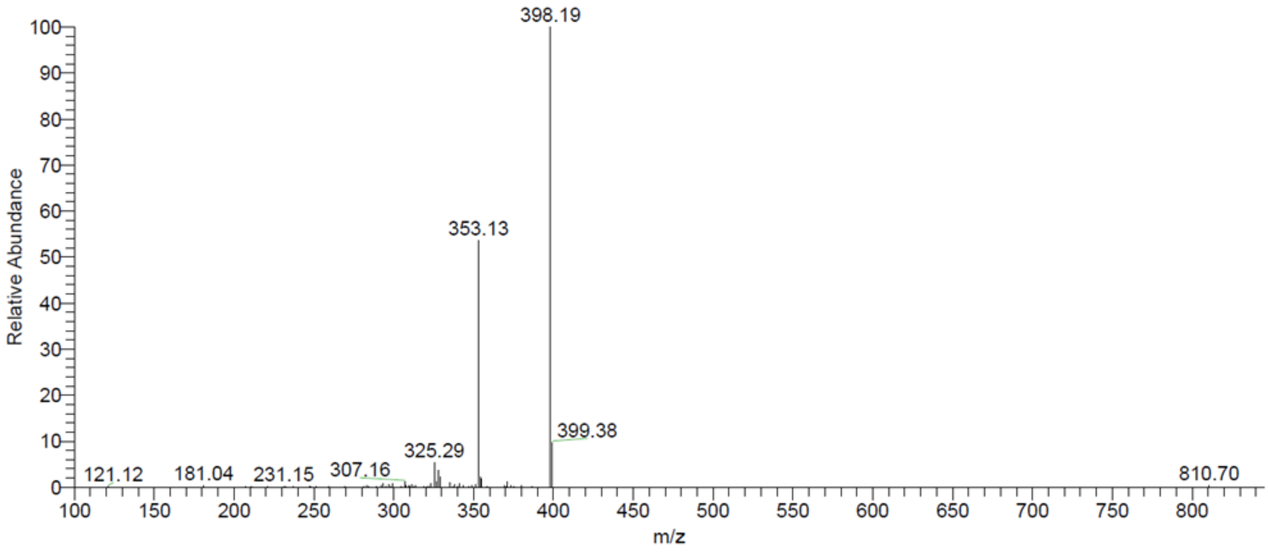

19. (ESI-MS, Positive mode): mass calcd. for **19** [M+H]$^+$ requires m/z = 412.51, found m/z = 412.25.

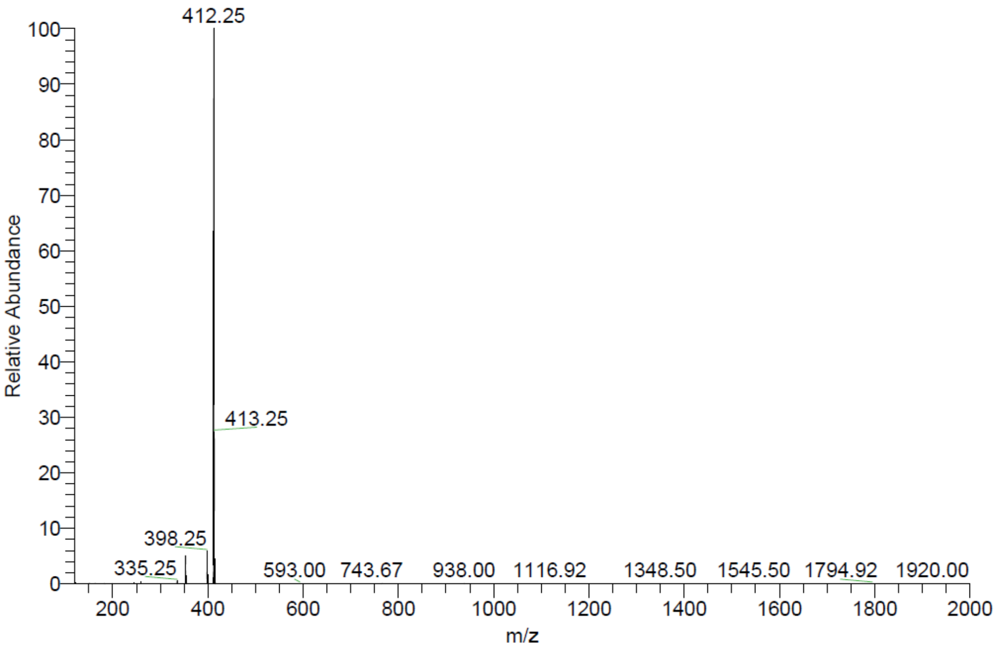

20. (ESI-MS, Negative mode): mass calcd. for **20** [M-H]$^-$ requires m/z = 369.4, found m/z = 369.25

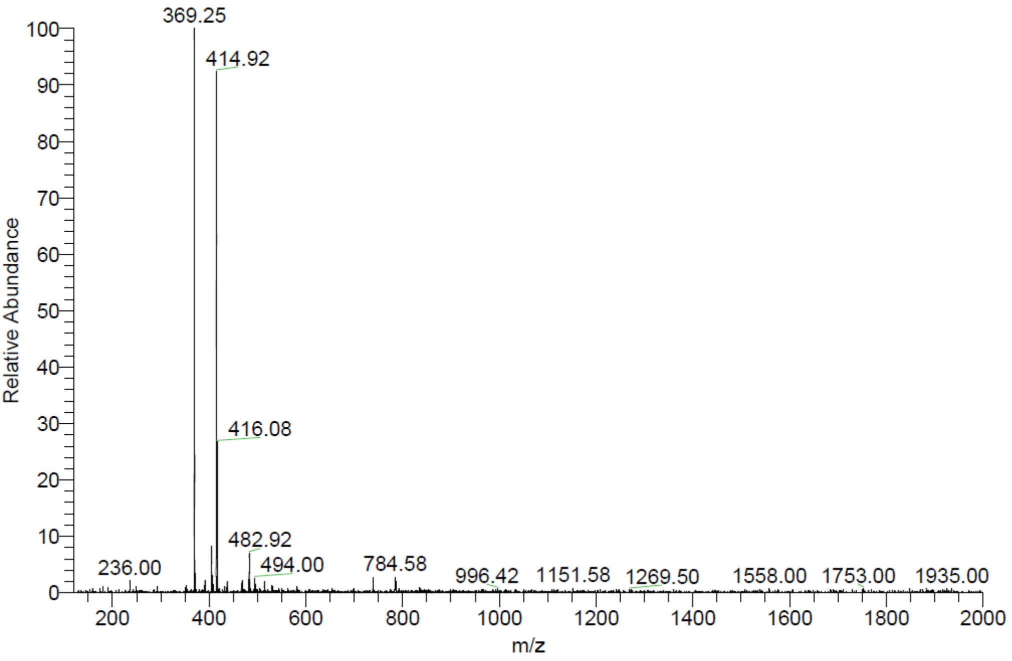

21. (ESI-MS, Positive mode): mass calcd. for **21** $[M+H]^+$ requires m/z = 466.24, found m/z = 466.33.

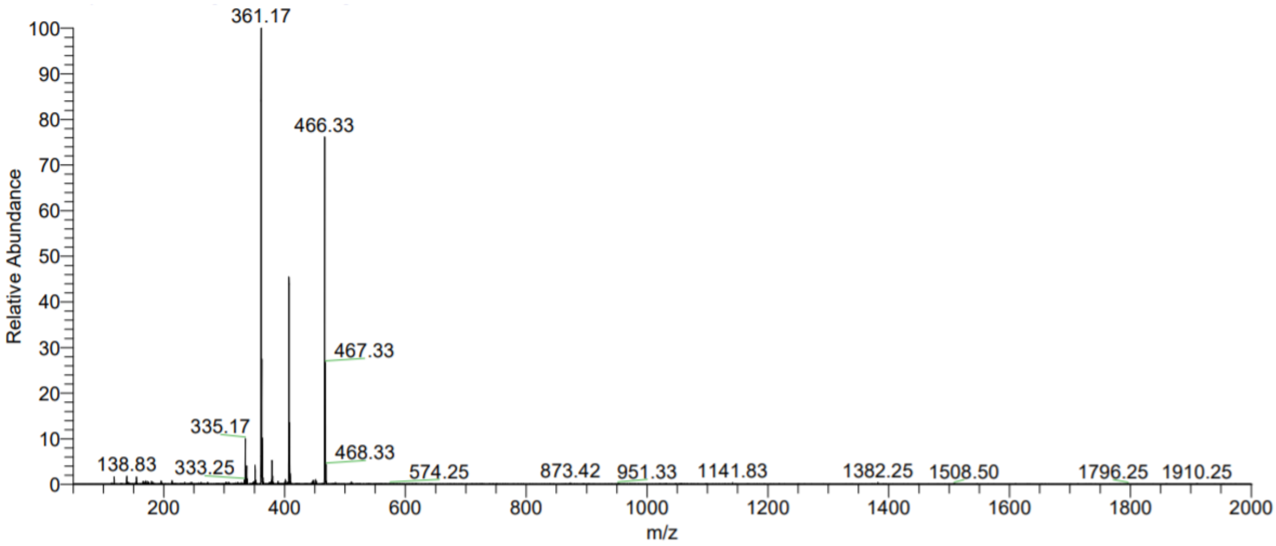

Supplement: Supplementary file 1 — Supporting Information [file CHEM-28-0-s001.pdf]
